# Psychedelics and the Human Receptorome

**DOI:** 10.1371/journal.pone.0009019

**Published:** 2010-02-02

**Authors:** Thomas S. Ray

**Affiliations:** Department of Zoology, University of Oklahoma, Norman, Oklahoma, United States of America; INSERM U862, France

## Abstract

We currently understand the mental effects of psychedelics to be caused by agonism or partial agonism of 5-HT_2A_ (and possibly 5-HT_2C_) receptors, and we understand that psychedelic drugs, especially phenylalkylamines, are fairly selective for these two receptors. This manuscript is a reference work on the receptor affinity pharmacology of psychedelic drugs. New data is presented on the affinity of twenty-five psychedelic drugs at fifty-one receptors, transporters, and ion channels, assayed by the National Institute of Mental Health – Psychoactive Drug Screening Program (NIMH-PDSP). In addition, comparable data gathered from the literature on ten additional drugs is also presented (mostly assayed by the NIMH-PDSP). A new method is introduced for normalizing affinity (K_i_) data that factors out potency so that the multi-receptor affinity profiles of different drugs can be directly compared and contrasted. The method is then used to compare the thirty-five drugs in graphical and tabular form. It is shown that psychedelic drugs, especially phenylalkylamines, are not as selective as generally believed, interacting with forty-two of forty-nine broadly assayed sites. The thirty-five drugs of the study have very diverse patterns of interaction with different classes of receptors, emphasizing eighteen different receptors. This diversity of receptor interaction may underlie the qualitative diversity of these drugs. It should be possible to use this diverse set of drugs as probes into the roles played by the various receptor systems in the human mind.

## Introduction

We currently understand the mental effects of psychedelics to be caused by agonism or partial agonism of 5-HT_2A_ (and possibly 5-HT_2C_) receptors (serotonin-2A and serotonin-2C receptors) [1]. This understanding was first developed in the 1980s [2]–[4] and has since been confirmed by a large body of evidence, as reviewed recently by Nichols [1]. However, many authors have commented that other receptors may also play a role [1], [3], [5]–[9]. In this post-genome era of high-throughput assays, it is time to take a broader view, move beyond the common-denominator approach [6], and begin to explore the role of other receptors in shaping the mental effects of psychedelics, especially the qualitative differences among them.

**Figure 1:**
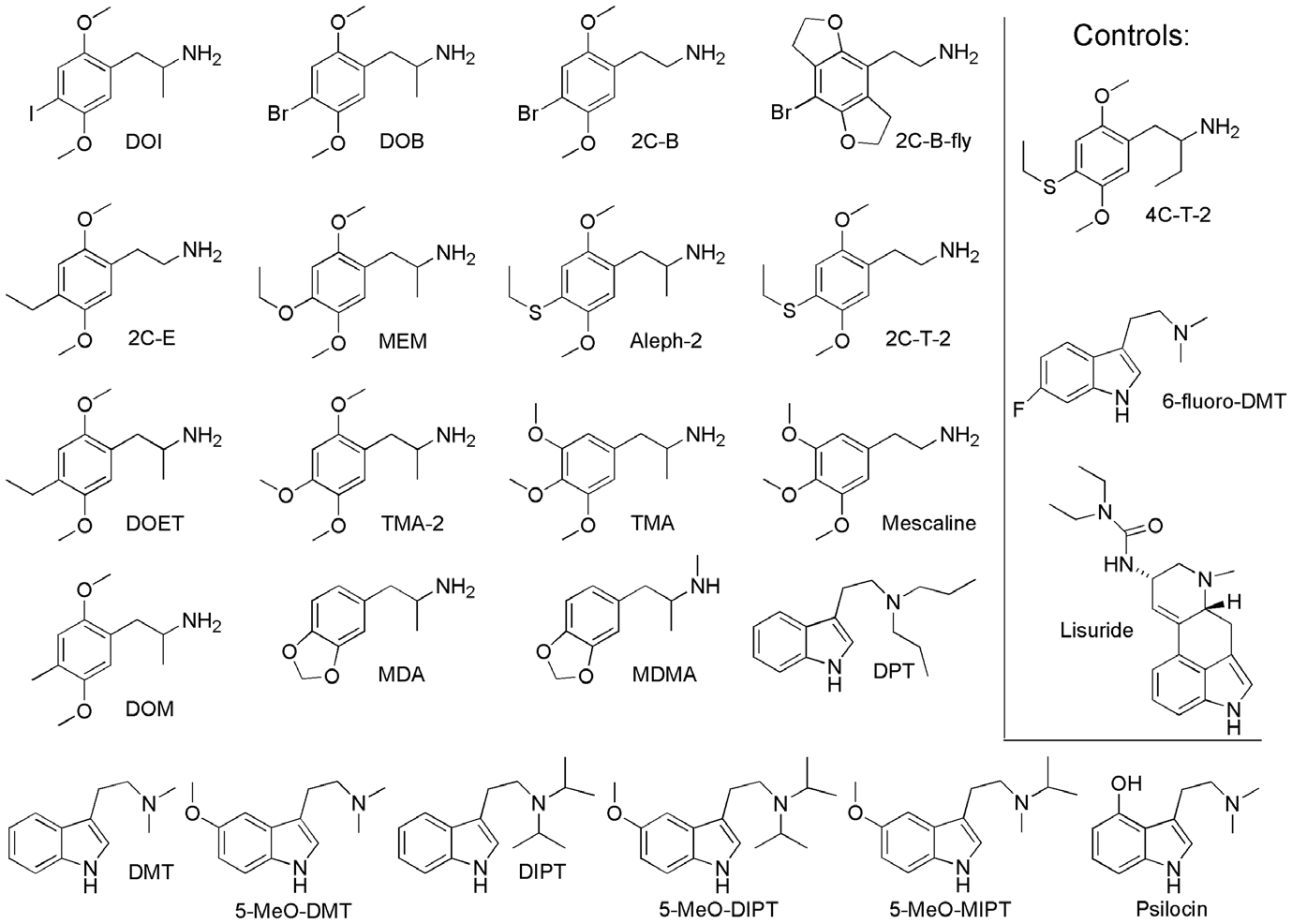
Twenty-five drugs assayed for this study by the NIMH-PDPS. Twenty-five drugs assayed for this study by the NIMH-PDPS against fifty-one receptors, transporters and ion-channels. The twenty-five drugs include sixteen phenylalkylamines, eight tryptamines, and one ergoline. The three control drugs on the right include one representative from each structural class, and are believed to be non-psychedelic.

**Figure 2:**
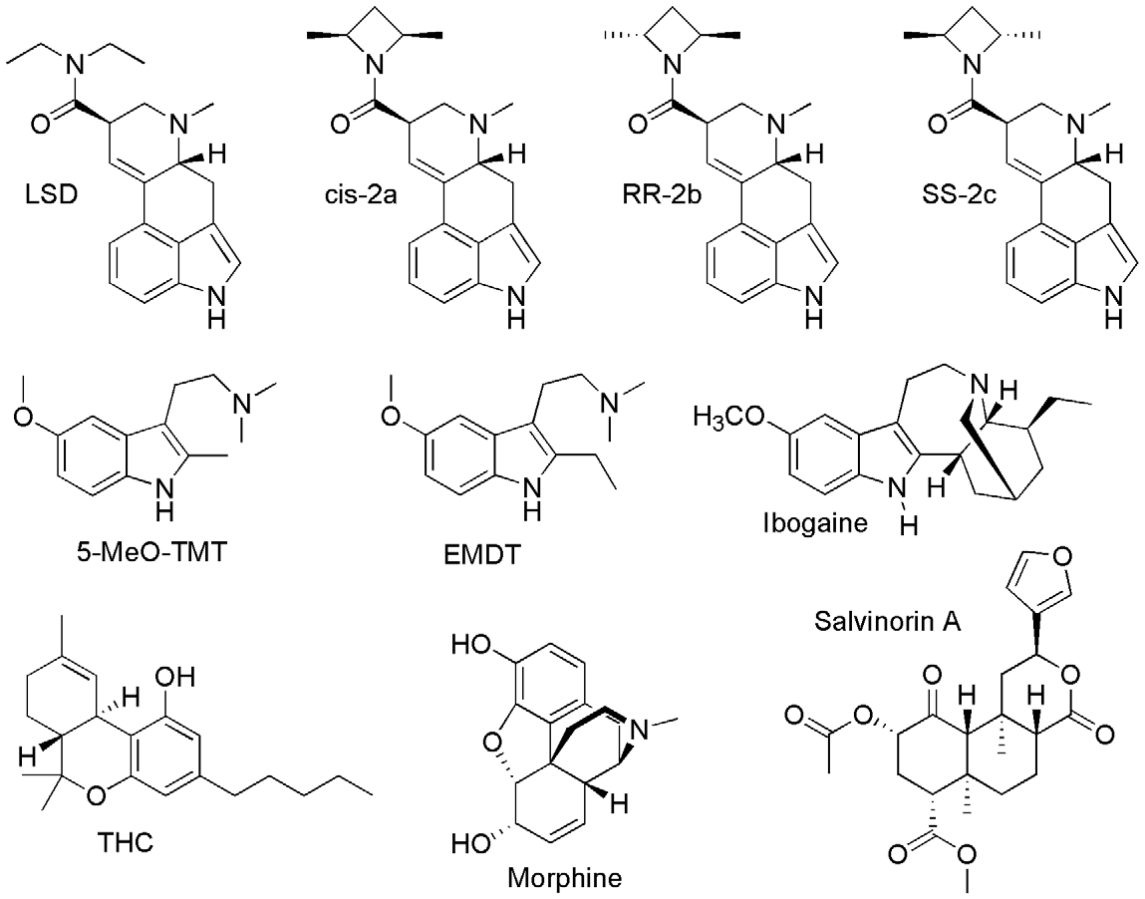
Ten drugs whose receptor profiles were collected from the literature. Ten drugs whose receptor profiles were collected from the literature. All but ibogaine, THC, and morphine were assayed by the NIMH-PDSP.

The objective of this paper is to present the receptor binding profiles of the thirty-five drugs (Fig. 1, Fig. 2) of this study in such a way that they can be easily compared in both their similarities and their differences. This is intended to serve as a reference work on the multi-receptor affinity pharmacology of psychedelic drugs. The tables and figures are the heart of this manuscript. Some of them have been included as “supporting information,” because they exceed the size limits of standard tables and figures. However, this supporting information is no less central to the manuscript than the standard tables and figures.

## Methods

### Data from Literature

Data on receptor interactions of ten compounds (Fig. 2) has been collected from the literature. The four ergolines (LSD, cis-2a, RR-2b, and SS-2c) were assayed by NIMH-PDSP against forty-three receptors, transporters and ion channels [10]. Salvinorin A was assayed by NIMH-PDSP against thirty receptors and transporters [11]. EMDT and 5-MeO-TMT were assayed by NIMH-PDSP against forty receptors, transporters and ion channels [12].

Receptor data for ibogaine (Table S1), morphine (Table 1) and THC (Table 2) was collected from a variety of sources. While ibogaine has been assayed at a wide variety of receptors, morphine and THC have not, so their data should be used with caution. Although morphine is not considered to be a psychedelic, and ibogaine, THC, and salvinorin A are not considered to be “classical hallucinogens,” these four compounds are included because they provide insights into additional receptor systems (salvinorin A – κ (kappa opioid receptor), ibogaine – σ (sigma receptor) and κ, THC – CB (cannabinoid receptor), morphine – μ (mu opioid receptor)). These additional compounds could also be thought of as active controls, as compared to the three presumably inactive controls of Fig. 1.

**Table 1:** Receptor affinity data for morphine.

Receptors	Hot Ligand	Source	Tissue	Ki(nM)	IC50(nM)	Reference
KOR	3H-U69,593	GUINEA PIG	ILEUM	217±49		PDSP; [17]
	3H-U69,593	Human	cloned	134±22		PDSP; [18]
	[Dmt]DALDA	Human	cloned	4.4±1.7		[19]
	DMAGO	Human	cloned	213±28		[19]
	[3H]U69593	rat	Brain	113±9		PDSP; [20]
				**50**		**average human**
MOR	3H-DAMGO	GUINEA PIG	ILEUM	1±0.04		PDSP; [17]
	3H-Diprenorphine	Human	cloned	2.06±0.48		PDSP; [18]
	3H-Dmt-DALDA	mouse	Brain	5.64±0.24		PDSP; [19]
	[Dmt]DALDA	human	cloned	0.172±0.026		[19]
	DMAGO	human	cloned	1.180±0.120		[19]
	[3H]DAMGO	rat	Brain	6.55±0.74		PDSP; [20]
			HEK-μ cells	2.2±0.5		[21]
	[3H]DAMGO	human	BE(2)-C memberanes	1.02±0.15		PDSP; [22]
		bovine	adrenals	1.86		[23]
				**0.81**		**average human**
DOR	3H-DSLET	MOUSE	vas deferens	32.6±3.7		PDSP; [17]
	3H-Naltrindole	Human	cloned	>10,000		PDSP; [18]
	[Dmt]DALDA	human	cloned	1670±40		[19]
	DMAGO	human	cloned	1430±20		[19]
	[3H][Ile5,6]deltorphin II	rat	Brain	217±19		PDSP; [20]
				278±49		[24]
	[3H]DPDPE	human	BE(2)-C memberanes	>100		PDSP; [22]
	[3H]enkephalin	rat	memberane		69.1±3.2	[25]
		bovine	adrenals	147.32		[23]
				**1545**		**average human**

Receptor affinity data for morphine collected from the literature. The columns identify the receptor, the radioligand used in determining affinity, the source species from which the receptor was used, the tissue from which the receptor was used, the K_i_ value in nanomoles or the IC50 (the molar concentration of an unlabeled agonist or antagonist that inhibits the binding of a radioligand by 50%, [26]) value in nanomoles, and the literature reference from which the data was obtained.

**Table 2:** Receptor affinity data for THC.

Receptors	hot ligand	source	tissue	Ki(nM)	Ref
CB1	3H-BAY 38-7271	HUMAN	CORTICAL MEMBRANES	13.7	PDSP; [27]
	3H-BAY 38-7271	HUMAN	CLONED	15.3	PDSP; [28]
	3H-CP-55940	HUMAN	CLONED	5.05	PDSP; [29]
				**10.19**	**average**
CB2	3H-BAY 38-7271	HUMAN	CLONED	25.06	PDSP; [28]
	3H-BAY 38-7271	HUMAN	CLONED	22.9	PDSP; [27]
	3H-CP-55940	HUMAN	CLONED	44.9	[30]
	3H-CP-55940	HUMAN	CLONED	3.13	PDSP; [29]
				**16.85**	**average**
sigma	3H-3-PPP,(+)	RAT	BRAIN	>100,000	PDSP; [31]

Receptor affinity data for THC collected from the literature. The columns identify the receptor, the radioligand used in determining affinity, the source species from which the receptor was used, the tissue from which the receptor was used, the K_i_ value in nanomoles, and the literature reference from which the data was obtained.

### New PDSP Binding Assays

For this study, the NIMH-PDSP (http://pdsp.med.unc.edu/) has assayed sixteen phenylalkylamines, eight tryptamines and one ergoline (twenty-two psychedelics and three controls, Fig. 1) against a panel of fifty-one receptors, transporters, and ion channels. The methodology has been described previously by Glennon et al. [12]. Each compound is initially assayed at 10 µM against each receptor, transporter or ion channel (primary assay). Those that induce >50% inhibition (“hit”) are then assayed at 1, 10, 100, 1,000, and 10,000 nM to determine K_i_ values (secondary assay). Each K_i_ value (equilibrium dissociation constant, concentration at which 50% of the hot ligand is displaced by the test ligand) is calculated from at least three replicated assays. Details of how individual assays were conducted can be found at the NIMH-PDSP web site: http://pdsp.med.unc.edu/pdspw/binding.php.

Table S2 shows raw K_i_ data for the current study combined with data collected from the literature for the ten additional compounds; a total of thirty-five drugs and sixty-seven receptors, transporters and ion channels which were assayed. The table has been divided into three sections.

The first section displays forty-two sites at which most compounds were assayed and at least one “hit” (K_i_ <10,000 nm) was found: 5ht1a (5-HT_1A_, serotonin-1A receptor), 5ht1b (5-HT_1B_, serotonin-1B receptor), 5ht1d (5-HT_1D_, serotonin-1D receptor), 5ht1e (5-HT_1E_, serotonin-1E receptor), 5ht2a (5-HT_2A_, serotonin-2A receptor), 5ht2b (5-HT_2B_, serotonin-2B receptor), 5ht2c (5-HT_2C_, serotonin-2C receptor), 5ht5a (5-HT_5A_, serotonin-5A receptor), 5ht6 (5-HT_6_, serotonin-6 receptor), 5ht7 (5-HT_7_, serotonin-7 receptor), D1 (D_1_, dopamine-1 receptor), D2 (D_2_, dopamine-2 receptor), D3 (D_3_, dopamine-3 receptor), D4 (D_4_, dopamine-4 receptor), D5 (D_5_, dopamine-5 receptor), Alpha1A (α_1A_, alpha-1A adrenergic receptor), Alpha1B (α_1B_, alpha-1B adrenergic receptor), Alpha2A (α_2A_, alpha-2A adrenergic receptor), Alpha2B (α_2B_, alpha-2B adrenergic receptor), Alpha2C (α_2C_, alpha-2C adrenergic receptor), Beta1 (β_1_, beta-1 adrenergic receptor), Beta2 (β_2_, beta-2 adrenergic receptor), SERT (serotonin transporter), DAT (dopamine transporter), NET (nor epinephrine transporter), Imidazoline1 (I_1_, imidazoline-1 receptor), Sigma1 (σ_1_, sigma-1 receptor), Sigma2 (σ_2_, sigma-2 receptor), DOR (delta opioid receptor), KOR (κ, kappa opioid receptor), MOR (μ, mu opioid receptor), M1 (M_1_, muscarinic-1 acetylcholine receptor), M2 (M_2_, muscarinic-2 acetylcholine receptor), M3 (M_3_, muscarinic-3 acetylcholine receptor), M4 (M_4_, muscarinic-4 acetylcholine receptor), M5 (M_5_, muscarinic-5 acetylcholine receptor), H1 (H_1_, histamine-1 receptor), H2 (H_2_, histamine-2 receptor), CB1 (CB_1_, cannabinoid-1 receptor), CB2 (CB_2_, cannabinoid-2 receptor), Ca+Channel (calcium+ ion channel), NMDA/MK801 (N-methyl D-aspartate glutamate receptor).

The second section displays seven sites at which most compounds were assayed, but at which there were no hits: 5ht3 (serotonin-3 receptor), H3 (histamine-3 receptor), H4 (histamine-4 receptor), V1 (vasopressin-1 receptor), V2 (vasopressin-2 receptor), V3 (vasopressin-3 receptor), GabaA (GABA-A receptor).

The third section displays the remaining eighteen sites, at which only a few compounds were assayed, and no hits were found: GabaB (GABA-B receptor), mGluR1a (mGluR1a metabotropic glutamate receptor), mGluR2 (mGluR2 metabotropic glutamate receptor), mGluR4 (mGluR4 metabotropic glutamate receptor), mGluR5 (mGluR5 metabotropic glutamate receptor), mGluR6 (mGluR6 metabotropic glutamate receptor), mGluR8 (mGluR8 metabotropic glutamate receptor), A2B2 (nicotinic a2/b2 acetylcholine receptor), A2B4 (nicotinic a2/b4 acetylcholine receptor), A3B2 (nicotinic a3/b2 acetylcholine receptor), A3B4 (nicotinic a3/b4 acetylcholine receptor), A4B2 (nicotinic a4/b2 acetylcholine receptor), A4B2** (nicotinic a4/b2** acetylcholine receptor), A4B4 (nicotinic a4/b4 acetylcholine receptor), BZP (a1) (GABA-BZP a1 receptor), EP3 (prostaglandin-3 receptor), MDR 1 (multidrug resistant p-Glycoprotein), PCP (PCP glutamate receptor).

### Activity Assays

For the twenty-five compounds of Fig. 1, the NIMH-PDSP also performed activity assays at 5-HT_2A_ and 5-HT_2C_. The E_max_ values (maximal activity) are relative to 5-HT (serotonin), measuring Ca++ mobilization. Ca++ flux assays were performed using a FLIPRTETRA. The activity assays were conducted with cell lines which have very high receptor expression levels (e.g. plenty of ‘spare receptors’). Under such conditions partial agonists will have considerable agonist activity. The data represent the mean ± variance of computer-derived estimates from single experiments done in quadruplicate. Thus, the four observations are averaged and a single estimate with error is provided (Table S3).

### Sources

The following compounds (Fig. 1) were used in the study:

- 2C-B, 4-Bromo-2,5-dimethoxyphenethylamine
- 2C-B-fly, 1-(8-Bromo-2,3,6,7-tetrahydrobenzo[1,2-b;4,5-b′]difuran-4-yl)2-aminoethane
- 2C-E, 4-Ethyl-2,5-dimethoxyphenethylamine
- 2C-T-2, 4-Ethylthio-2,5-dimethoxyphenethylamine
- ALEPH-2, (±)-4-Ethylthio-2,5-dimethoxyamphetamine
- 4C-T-2, 4-Ethylthio-2,5-dimethoxyphenylbutylamine
- MEM, (±)-2,5-Dimethoxy-4-ethoxyamphetamine
- TMA-2: (±)-2,4,5-Trimethoxamphetamine
- TMA: (±)-3,4,5-Trimethoxamphetamine
- mescaline: 3,4,5-Trimethoxyphenethylamine
- DOB: (±)-2,5-Dimethoxy-4-bromoamphetamine
- DOI: (±)-2,5-Dimethoxy-4-iodoamphetamine
- DOM: (±)-2,5-Dimethoxy-4-methylamphetamine
- DOET: (±)-2,5-Dimethoxy-4-ethylamphetamine
- MDA: (±)-3,4-Methylenedioxyamphetamine
- MDMA: (±)-3,4-Methylenedioxymethamphetamine
- DMT: N,N-Dimethyltryptamine
- 5-MeO-DMT: 5-Methoxy-N,N-dimethyltryptamine
- DPT: N,N-Dipropyltryptamine
- 5-MeO-MIPT: 5-Methoxy-N-methyl-N-isopropyltryptamine
- DIPT: N,N-Diisopropyltryptamine
- 5-MeO-DIPT: 5-Methoxy-N,N-diisopropyltryptamine
- 6-fluoro-DMT: 6-Fluoro-N,N-dimethyltryptamine
- psilocin: 4-Hydroxy-N,N-dimethyltryptamine
- lisuride

5-MeO-DMT, and DOI were purchased from Sigma. DOB, DOET, mescaline, TMA, MDA, MDMA, and psilocin were provided as gifts by the National Institute on Drug Abuse Drug Supply Program. 2C-B, 2C-B-fly, MEM, 4C-T-2, 5-MeO-MIPT, 6-fluoro-DMT, TMA-2, and lisuride were provided as gifts by Dave Nichols. DMT and DOM were provided as gifts by Richard Glennon. 2C-E, 2C-T-2, Aleph-2, DIPT, 5-MeO-DIPT, and DPT were provided as gifts by Alexander Shulgin.

### Normalization

The raw K_i_ values are distributed over several orders of magnitude, thus a log transformation is a good first step in the analysis. In addition, higher affinities produce lower K_i_ values, thus it is valuable to calculate: pK_i_ = −log_10_(K_i_). Higher affinities have higher pK_i_ values, and each unit of pK_i_ value corresponds to one order of magnitude of K_i_ value. Table S4 presents the raw data transformed into pK_i_ values. Generally, the highest K_i_ value generated by NIMH-PDSP is 10,000, which produces a pK_i_ value of −4 (although a value of 10,450 was reported for 5-MeO-TMT). For non-PDSP data gathered from the literature, some K_i_ values greater than 10,000 are reported (i.e. 12,500, 14,142, 22,486, 39,409 and 70,000 for ibogaine).

When the primary assay did not produce >50% inhibition, the K_i_ value is treated as >10,000. When the primary assay hit, but the secondary assay was not performed, the K_i_ value is also treated as >10,000. If a secondary assay produced a K_i_ value significantly greater than 10,000, it is usually also reported as >10,000. The lowest K_i_ value in the data set of this study is 0.3 (lisuride at 5-HT_1A_) and the highest value is 70,000 (ibogaine at D_3_), thus collectively, the data in this study cover nearly six orders of magnitude of K_i_ values. However, ignoring values reported as >10,000, the K_i_ values for a single drug in this study never exceed four orders of magnitude in range.

The goal of the normalization used in this study is to factor out potency, in order to allow easy comparison of the multi-receptor affinity profiles of different drugs. The normalization will adjust the highest pK_i_ value for each drug to a value of 4, and set all K_i_ values reported as >10,000 to a value of zero. K_i_ values actually measured as greater than 10,000 are not set to zero (i.e. 5-MeO-TMT and ibogaine). We will call this normalized value npK_i_. Let the maximum pK_i_ value for each drug be called pK_iMax_. For each individual drug:

- If K_i_ treated as >10,000, then npK_i_ = 0
- npK_i_ = 4+pK_i_−pK_iMax_

With this normalization:

- higher affinities have higher values
- affinities too low to be measured will be reported as zero
- for each drug, the highest affinity will be set to a value of 4
- each unit of npK_i_ value represents one order of magnitude of K_i_ value
- potency is factored out so that drugs of different potencies can be directly compared

This normalization effectively factors out the absolute potency of each drug, and allows us to focus on the relative affinities of each drug at each receptor.

### Perceptibility

**Figure 3:**
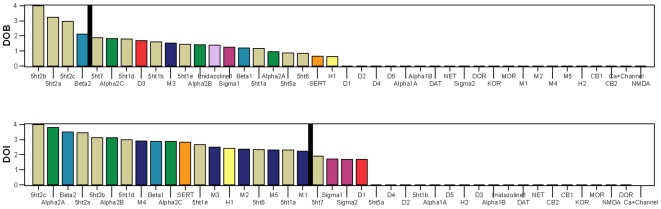
Receptor affinity profiles of DOB and DOI, ordered by decreasing affinity. The vertical axis is normalized pK_i_ (npK_i_). Horizontal axis is a list of forty-two receptors, arranged in order of decreasing affinity for each individual drug. Colors correspond to classes of receptors, and are the same as used in Fig. S1. The black vertical bars represent a 100-fold drop in affinity relative to the receptor with the highest affinity. As a rule of thumb, this is presumed to be the limit of perceptible receptor interaction. Receptors to the right of the black bar should be imperceptible, while receptors to the left of the black bar should be perceptible, increasingly so the further left they are.

It will also be seen that many psychedelic drugs interact with a large number of receptors. Fig. 3 shows the ranked distributions of npK_i_ values for DOB and DOI, and the same data is listed below in numerical form (0.00 means K_i_ >10,000, ND means the data is not available):

**DOB:** 4.00 5ht2b, 3.23 5ht2a, 2.97 5ht2c, 2.11 Beta2, 1.89 5ht7, 1.82 Alpha2C, 1.79 5ht1d, 1.68 D3, 1.62 5ht1b, 1.53 M3, 1.44 5ht1e, 1.41 Alpha2B, 1.39 Imidazoline1, 1.25 Sigma1, 1.21 Beta1, 1.18 5ht1a, 0.96 Alpha2A, 0.87 5ht5a, 0.85 5ht6, 0.66 SERT, 0.63 H1; **0.00:** D5, D2, D4, NET, D1, Alpha1B, Sigma2, DOR, KOR, MOR, M1, M2, DAT, M4, M5, Alpha1A, H2, CB2, CB1, Ca+Channel, NMDA

**DOI:** 4.00 5ht2c, 3.79 Alpha2A, 3.52 Beta2, 3.44 5ht2a, 3.13 Alpha2B, 3.13 5ht2b, 3.00 5ht1d, 2.90 M4, 2.89 Beta1, 2.88 Alpha2C, 2.83 SERT, 2.66 5ht1e, 2.51 M3, 2.42 H1, 2.36 M2, 2.34 5ht6, 2.32 M5, 2.31 5ht1a, 2.23 M1, 1.90 5ht7, 1.73 Sigma1, 1.70 Sigma2, 1.67 D1; **0.00:** 5ht1b, DAT, Imidazoline1, NET, 5ht5a, DOR, KOR, MOR, Alpha1B, D2, D3, D4, D5, Alpha1A, H2, CB2, CB1, NMDA; **ND:** Ca+Channel

For potent compounds like DOB and DOI, it is possible to measure K_i_ values over nearly a full four orders of magnitude range of affinity. However, not all of these affinities are able to produce perceptible mental effects. As a rule of thumb, 100-fold affinity is considered truly selective. Thus, receptors with npK_i_ values below about 2.0 should not have perceptible mental effects. In Fig. 3, a black vertical bar represent a 100-fold drop in affinity relative to the receptor with the highest affinity, and divides those npK_i_ values greater than 2.0 (on the left) from those 2.0 or less (on the right). This is presumed to be the limit of perceptible receptor interaction. Receptors to the right of the black bar should be imperceptible, while receptors to the left of the black bar should be perceptible, increasingly so the further left they are. In spite of the long tail of affinities, DOB is effectively selective for the three 5-HT_2_ (serotonin-2) receptors (Beta2 falls at the approximate limit of perceptibility), while DOI by contrast has nineteen receptors in the presumed perceptible range, although they should not all be equally perceptible.

### Breadth

An index of the breadth (or inverse of selectivity), B, of the binding profiles of the individual drugs or receptors can be constructed by summing the forty-two npK_i_ values for each drug, or the thirty-five npK_i_ values for each receptor. If a drug were absolutely selective, binding at only one receptor (e.g. salvinorin A), it would have the minimal B value of 4, regardless of the absolute affinity of the drug for its one receptor. If a drug bound with equal affinity to all forty-two receptors, it would have the maximum B value of 4×42 = 168, regardless of its absolute receptor affinities.

It is not clear that a simple sum of npK_i_ values is the best index of breadth. In this method, four receptors with K_i_ values of 1,000 collectively carry the same weight as one receptor with a K_i_ value of 1. This may not be a realistic equivalence. Thus we will include three measures of breadth:
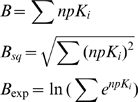
B_sq_ and B_exp_ give greater weight to higher affinity (lower K_i_) values. Regression analysis of receptor affinity vs. potency in humans suggests that B_sq_ is the most meaningful breadth statistic. Table S5 and Table S6 present the raw K_i_ data converted into npK_i_ values, for both the individual receptors, and groups of receptors summed using the B_sq_ statistic.

### Proportional Breadth

In addition to looking at the breadth of interaction of individual drugs with multiple receptors, it may be of value to look at an individual drug's interaction with one receptor or group of receptors, as a proportion of the drug's total interaction with all receptors.

In order to compute the proportion for and individual receptor or a group of receptors, we divide the sum of squares of npK_i_ values for the group of receptors, by the sum of squares of npK_i_ values for all receptors:
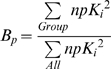
For example, to compute this proportion for “5-HT” receptors, we divide the squares of the values in the “5-HT” column of Table S5 (for LSD, 11.13^2^ = 123.9), by the squares of the values in the center column (“B_sq_”) of Table 3 (for LSD, 13.12^2^ = 172.1); 123.9/172.1 = 0.719 for LSD. We will call this proportion B_p_. The proportional breadth data is displayed in Table S7 and Table S8.

**Table 3:** Thirty-five drugs arranged in order of decreasing breadth, increasing selectivity.

B	Drug	B_sq_	Drug	B_exp_	Drug
67.5	6-F-DMT	14.19	6-F-DMT	6.22	6-F-DMT
63.5	DPT	13.34	DMT	6.16	DMT
61.6	DOI	13.30	DPT	6.12	LSD
56.4	LSD	13.21	DOI	6.02	DPT
54.3	DMT	13.12	LSD	6.02	DOI
50.5	lisuride	11.88	lisuride	5.93	TMA
45.3	2C-E	11.61	2C-E	5.85	lisuride
45.2	cis-2a	11.55	TMA	5.81	2C-E
45.2	5-MeO-MIPT	11.37	2C-B	5.77	2C-B
43.9	2C-B	11.29	cis-2a	5.75	cis-2a
42.1	psilocin	11.00	5-MeO-MIPT	5.70	5-MeO-MIPT
40.5	2C-T-2	10.71	psilocin	5.60	psilocin
38.1	TMA	10.21	DIPT	5.52	DIPT
37.6	RR-2b	9.85	5-MeO-DIPT	5.49	5-MeO-DIPT
34.9	DIPT	9.80	RR-2b	5.44	4C-T-2
34.7	5-MeO-DMT	9.65	2C-T-2	5.44	RR-2b
34.5	DOB	9.64	4C-T-2	5.44	MDMA
33.4	SS-2c	9.50	MDMA	5.41	DOET
33.3	DOET	9.35	ibogaine	5.38	mescaline
32.5	2C-B-fly	9.32	DOET	5.36	2C-T-2
32.1	5-MeO-DIPT	9.00	5-MeO-DMT	5.36	5-MeO-DMT
31.2	ibogaine	8.85	SS-2c	5.35	ibogaine
31.1	4C-T-2	8.67	2C-B-fly	5.29	2C-B-fly
28.2	MDMA	8.67	mescaline	5.24	5-MeO-TMT
28.1	DOM	8.44	DOB	5.22	SS-2c
27.0	Aleph-2	8.41	5-MeO-TMT	5.16	DOB
22.9	5-MeO-TMT	8.29	DOM	5.10	DOM
21.1	mescaline	7.89	MDA	5.07	MDA
20.4	MDA	7.30	Aleph-2	4.94	Aleph-2
18.4	EMDT	7.22	EMDT	4.86	EMDT
13.0	TMA-2	6.60	TMA-2	4.78	TMA-2
10.3	MEM	5.50	THC	4.59	THC
7.8	THC	5.40	MEM	4.37	MEM
6.9	morphine	4.63	morphine	4.19	morphine
4.0	salvinorin A	4.00	salvinorin A	4.00	salvinorin A

The thirty-five drugs are arranged in order of decreasing breadth and increasing selectivity, based on the breadth indices B, B_sq_, and B_exp_. Although the three indices provide different orderings, the orderings are quite similar at the two extremes of the table (greatest and least breadth) where most of the attention is likely to be focused. The drugs with the broadest receptor interactions (least selective) are found at the tops of the columns, and the drugs with the narrowest receptor interactions (most selective) are found at the bottoms of the columns.

### Truncated Receptor Profiles

The NIMH-PDSP generally does not measure K_i_ values greater than 10,000 nm, because at those concentrations, there is a great deal of non-specific binding which invalidates the measurement of receptor affinity. This creates a problem for drugs whose best-hit has a K_i_ value of greater that 100 nm (TMA, mescaline, TMA-2, DIPT, MDMA, 5-MeO-DIPT, ibogaine). For these drugs, the range of K_i_ values that can be measured by the NIMH-PDSP is less than the 100-fold presumed perceptible range, and therefore, the lowest measurable npK_i_ value is greater than the presumed limit of perceptibility at 2.00. Table 4 shows for each drug, the lowest K_i_ value measured (K_i_Min) which is the best-hit, the best-hit receptor (K_i_MinR), the theoretically lowest measurable npK_i_ value (npK_i_Lim), the lowest actually measured npK_i_ value (npK_i_Min), and the receptor where the lowest npK_i_ value was actually measured (npK_i_MinR). Drugs that have both a K_i_Min value of greater that 100 nm and an npK_i_Min value greater than 2.00 have truncated receptor affinity profiles.

**Table 4:** Truncated receptor profiles for thirty-five drugs.

Drug	KiMin	KiMinR	npKiLim	npKiMin	npKiMinR
mescaline	745.3	Alpha2C	2.87	2.92	Alpha2A
TMA	476.6	5ht2b	2.68	2.98	Alpha2C
MDMA	219.7	Imidazoline1	2.34	2.43	M4
ibogaine	206	Sigma2	2.31	1.47	D3
TMA-2	154.4	5ht2b	2.19	2.58	5ht2c
5-MeO-DIPT	132.4	5ht1a	2.12	2.15	Sigma1
DIPT	120.5	5ht1a	2.08	2.51	5ht1d
MDA	91	5ht2b	1.96	2.15	5ht2c
DMT	87.5	5ht7	1.94	2.23	Sigma1
MEM	64.5	5ht2b	1.81	1.95	5ht7
5-MeO-TMT	60	5ht6	1.78	1.76	5ht5a
4C-T-2	58.1	5ht2b	1.76	1.77	5ht1e
DOI	45.8	5ht2c	1.66	1.67	D1
DPT	31.8	5ht1a	1.50	1.54	D2
6-F-DMT	25.6	5ht6	1.41	1.56	Sigma2
2C-E	25.1	5ht2b	1.40	1.88	D2
EMDT	16	5ht6	1.20	1.54	5ht5a
DOET	14.4	5ht1a	1.16	1.17	Sigma1
2C-B	13.5	5ht2b	1.13	1.28	D3
5-MeO-MIPT	12.3	5ht1a	1.09	1.28	SERT
DOM	11.7	5ht2b	1.07	1.16	5ht6
THC	10.19	CB1	1.01	3.78	CB2
2C-T-2	6	5ht2b	0.78	0.81	Beta1
psilocin	4.7	5ht2b	0.67	1.03	Alpha2C
salvinorin A	4.3	KOR	0.63	4	KOR
LSD	3.9	5ht1b	0.59	0.65	Alpha1B
DOB	3.9	5ht2b	0.59	0.63	H1
cis-2a	2.3	5ht1a	0.36	0.7	Beta2
RR-2b	2	5ht1b	0.30	0.59	Alpha1A
5-MeO-DMT	1.9	5ht1a	0.28	0.69	5ht2b
Aleph-2	1.6	5ht2b	0.20	0.3	M5
2C-B-fly	0.9	5ht2b	−0.05	0.12	D2
morphine	0.81	MOR	−0.09	0.72	DOR
SS-2c	0.4	5ht1a	−0.40	0.2	H1
lisuride	0.3	5ht1a	−0.52	1.08	Alpha1B

Table 4 shows for each drug, the lowest K_i_ value measured (K_i_Min) which is the best-hit, the best-hit receptor (K_i_MinR), the theoretically lowest measurable npK_i_ value (npK_i_Lim), the lowest actually measured npK_i_ value (npK_i_Min), and the receptor where the lowest npK_i_ value was actually measured (npK_i_MinR). Drugs that have both a K_i_Min value of greater that 100 nm and an npK_i_Min value greater than 2.00, have truncated receptor affinity profiles.

Seven drugs have best-hit K_i_ values of greater that 100 nm: TMA, mescaline, TMA-2, DIPT, MDMA, 5-MeO-DIPT, ibogaine. For these seven drugs, the perceptible receptor profile is truncated due to the methodological limitations of the NIMH-PDSP, to the extent to which the npK_i_Min value is greater than 2.00. Note that for ibogaine, whose best hit is 206 nm, the npK_i_Min value is 1.47, indicating that the receptor profile is not fully truncated, because several K_i_ values above 10,000 nm were gathered from the literature (however, ibogaine has not received a full receptorome screening, and thus its receptor profile must be considered incomplete for other reasons).

Six drugs have both a K_i_Min value of greater that 100 nm and an npK_i_Min value greater than 2.00. For 5-MeO-DIPT, the npK_i_Min value (2.15) is close to the presumed perceptibility limit, thus we can consider its receptor profile to be complete. For TMA-2, DIPT, and MDMA, the npK_i_Min values (2.58, 2.51, 2.43 respectively) fall in the weak region of the presumed perceptibility range. Although these three receptor profiles are truncated, the missing data may be of little consequence. For TMA and mescaline, the npK_i_Min values (2.98. 2.92 respectively) fall in the moderate region of the presumed perceptibility range. We must consider these two truncated receptor profiles to be truly incomplete, with potential consequences for our interpretations of the properties of these two drugs. The receptor profiles of some other drugs are incomplete due to holes in the NIMH-PDPS data set. Morphine and THC have not been broadly assayed, and must also be considered to be incomplete.

## Results

### Normalized Affinity Data

Fig. S1 shows the simplest view of the normalized affinity data. The drugs in Fig. S1 are ordered to correspond roughly to similarity of structure and receptor affinity profiles. Colors correspond to classes of receptors. It can be seen at a glance that most, but not all of the drugs interact strongly with the serotonin receptors (beige), certain drugs interact strongly with the dopamine receptors (red), others with the adrenergic receptors (green), yet others with the histamine receptors (yellow), etc.

### Breadth

**Figure 4:**
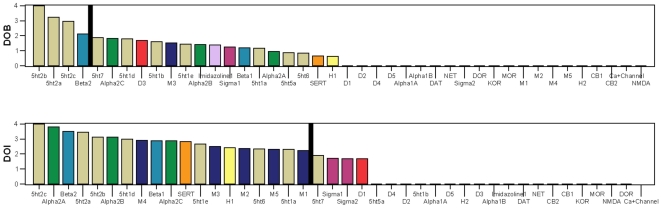
Thirty-five drugs arranged in order of decreasing breadth, increasing selectivity. The thirty-five drugs are arranged in order of decreasing breadth and increasing selectivity, based on the breadth indices B, B_sq_, and B_exp_. Although the three indices provide different orderings, the orderings are quite similar at the two extremes of the table (greatest and least breadth) where most of the attention is likely to be focused. The drugs with the broadest receptor interactions (least selective) are found at the left of the figure, and the drugs with the narrowest receptor interactions (most selective) are found at the right of the figure.

In Table 3, the thirty-five drugs are arranged in order of decreasing breadth and increasing selectivity, based on the three breadth indices B, B_sq_, and B_exp_. Although the three indices provide different orderings, the orderings are quite similar at the two extremes of the table (greatest and least breadth) where most of the attention is likely to be focused. The drugs with the broadest receptor interactions (least selective) are found at the tops of the columns, and the drugs with the narrowest receptor interactions (most selective) are found at the bottoms of the columns. Regression analysis suggests that B_sq_ is the best statistic for combining receptors, therefore the B_sq_ statistic will be used in most of the breadth analyses to follow. The B, B_sq_ and B_exp_ data of Table 3 is presented graphically in Fig. 4.

### Profiles of Drugs

The relative breadth or selectivity of the thirty-five drugs is nicely visualized in Fig. S2, in which for each drug, the bars representing forty-two receptors are arranged in order of decreasing size. The drugs are arranged in order of decreasing breadth, based on the B_sq_ values of Table 3 and Fig. 4. Drugs at the top of the figure have the broadest receptor interactions (least selective), while drugs at the bottom of the figure have the narrowest receptor interactions (most selective). Colors correspond to classes of receptors, and are the same as used in Fig. S1. Scanning Fig. S2 from top to bottom shows the variation in breadth of receptor interactions between drugs. It can also be seen that some distributions are relatively convex (e.g. DMT & LSD), while others are relatively concave (e.g. DOB & 5-MeO-DMT). Convexity tends to increase breadth, while concavity tends to decrease breadth.

It is also useful to present the npK_i_ data of Fig. S2 in numerical format. In the listing below, the npK_i_ values for each drug are arranged in decreasing order. A value of 0.00 means that the K_i_ value was measured as >10,000 nm. “ND” indicates that the data is not available. The 5-HT_2A_ and 5-HT_2C_ receptors are also highlighted in bold font for easier location. npK_i_ values below about 2.0 should be imperceptible, while values above about 2.0 should be perceptible, and the higher the npK_i_ value, the more perceptible a receptor should be.

**6-F-DMT:** 4.00 5ht6, 3.93 5ht2b, 3.80 5ht7, 3.74 H1, 3.66 5ht1d, 3.25 SERT, 3.24 Alpha2C, 3.17 Alpha1A, 3.07 5ht1b, 2.99 Alpha2B, 2.81 5ht1a, 2.74 5ht1e, 2.67 D1, 2.62 D2, **2.58 5ht2c**, **2.47 5ht2a**, 2.47 D3, 2.45 Imidazoline1, 2.44 H2, 2.43 5ht5a, 2.25 D4, 1.61 D5, 1.57 Sigma1, 1.56 Sigma2; **0.00:** DAT, Beta1, NET, Alpha2A, CB2, CB1, Ca+Channel, Beta2, M2, M3, M4, M5, M1, Alpha1B, NMDA; **ND:** DOR, MOR, KOR

**DMT:** 4.00 5ht7, 3.97 5ht1d, 3.91 5ht2b, 3.53 Alpha2B, 3.53 Alpha2C, 3.51 D1, **3.42 5ht2c**, 3.28 5ht1e, 3.25 5ht6, 3.16 5ht5a, 3.13 Imidazoline1, 2.95 Alpha1B, 2.75 Alpha2A, 2.70 Alpha1A, **2.58 5ht2a**, 2.37 SERT, 2.23 Sigma1; **0.00:** 5ht1a, D4, D5, Beta1, D2, D3, DAT, NET, 5ht1b, Beta2, Sigma2, CB2, KOR, Ca+Channel, M1, M2, M3, M4, M5, H2, CB1; **ND:** H1, DOR, MOR, NMDA

**DPT:** 4.00 5ht1a, 3.88 5ht2b, 3.41 H1, 3.31 SERT, 3.05 5ht7, 2.97 Imidazoline1, 2.97 Alpha2B, 2.90 Sigma1, 2.86 Alpha1B, 2.84 Alpha2A, 2.79 Alpha2C, 2.71 5ht1d, 2.57 5ht1b, 2.56 Alpha1A, 2.37 D3, 2.33 DAT, **2.31 5ht2c**, 2.20 D4, 2.13 5ht1e, **2.09 5ht2a**, 2.04 Sigma2, 1.86 5ht5a, 1.85 5ht6, 1.54 D2; **0.00:** D1, D5, NET, Beta1, DOR, KOR, MOR, Beta2, M2, M3, M4, M5, M1, H2, CB2, CB1, Ca+Channel, NMDA

**DOI: 4.00 5ht2c**, 3.79 Alpha2A, 3.52 Beta2, **3.44 5ht2a**, 3.13 Alpha2B, 3.13 5ht2b, 3.00 5ht1d, 2.90 M4, 2.89 Beta1, 2.88 Alpha2C, 2.83 SERT, 2.66 5ht1e, 2.51 M3, 2.42 H1, 2.36 M2, 2.34 5ht6, 2.32 M5, 2.31 5ht1a, 2.23 M1, 1.90 5ht7, 1.73 Sigma1, 1.70 Sigma2, 1.67 D1; **0.00:** 5ht1b, DAT, Imidazoline1, NET, 5ht5a, DOR, KOR, MOR, Alpha1B, D2, D3, D4, D5, Alpha1A, H2, CB2, CB1, NMDA; **ND:** Ca+Channel

**LSD:** 4.00 5ht1b, 3.77 5ht7, 3.75 5ht6, 3.73 5ht1a, 3.70 5ht1d, 3.64 5ht5a, **3.54 5ht2a**, 3.16 D3, 3.11 5ht2b, **3.11 5ht2c**, 2.93 Alpha2A, 2.62 5ht1e, 2.55 D2, 2.39 D4, 2.34 D1, 2.05 D5, 1.54 Alpha1A, 1.40 H1, 1.39 Beta1, 1.05 Beta2, 0.65 Alpha1B; **0.00:** KOR, DOR, DAT, SERT, MOR, NET; **ND:** Sigma2, Alpha2B, Alpha2C, Imidazoline1, M1, M2, M3, M4, M5, Sigma1, H2, CB2, CB1, Ca+Channel, NMDA

**lisuride:** 4.00 5ht1a, 3.88 Alpha2C, 3.78 Alpha2B, 3.22 Alpha2A, 3.01 5ht2b, 2.99 5ht5a, 2.90 D4, **2.74 5ht2a**, 2.65 D2, 2.64 5ht7, 2.61 5ht6, 2.56 Beta1, 2.27 5ht1b, 2.09 Alpha1A, 1.93 Beta2, 1.83 5ht1e, 1.59 D5, 1.42 H2, 1.34 D3, 1.08 Alpha1B; **0.00:** 5ht1d, **5ht2c**, D1, DAT, NET, Imidazoline1, M1, SERT, CB2, KOR, MOR, M3, M2, M5, M4, CB1, Ca+Channel; **ND:** Sigma1, H1, Sigma2, DOR, NMDA

**2C-E:** 4.00 5ht2b, **3.76 5ht2a**, 3.54 5ht1d, 3.44 Alpha2C, **3.38 5ht2c**, 3.00 5ht1b, 2.91 Alpha2B, 2.91 5ht1a, 2.77 5ht7, 2.71 Alpha2A, 2.60 5ht1e, 2.27 D3, 2.16 M5, 1.99 M3, 1.93 5ht6, 1.88 D2; **0.00:** D1, Alpha1A, Alpha1B, 5ht5a, Beta1, M1, SERT, D4, NET, Imidazoline1, H1, Sigma2, DOR, KOR, MOR, NMDA, M2, DAT, M4, D5, CB2, H2, Ca+Channel, CB1; **ND:** Sigma1, Beta2

**TMA:** 4.00 5ht2b, 3.95 Sigma2, 3.95 Sigma1, 3.80 5ht7, 3.45 5ht1a, 3.36 Alpha2A, 3.22 5ht1b, 3.20 5ht1d, 3.15 5ht1e, **3.02 5ht2c**, 2.98 Alpha2C; **0.00: 5ht2a**, 5ht6, D4, D1, Alpha1A, D2, D3, Alpha2B, D5, Beta1, Beta2, SERT, DAT, NET, Imidazoline1, Alpha1B, 5ht5a, DOR, KOR, MOR, NMDA, M2, CB2, CB1, M5, H1, H2; **ND:** M3, M4, Ca+Channel, M1

**2C-B:** 4.00 5ht2b, 3.71 5ht1d, **3.69 5ht2a**, **3.18 5ht2c**, 3.12 Alpha2C, 3.11 5ht1b, 3.05 5ht1e, 2.81 5ht7, 2.75 5ht1a, 2.64 Alpha2A, 2.63 5ht6, 2.31 Alpha2B, 2.22 M3, 1.80 Imidazoline1, 1.60 D2, 1.28 D3; **0.00:** D1, 5ht5a, Alpha1B, D5, NMDA, M1, SERT, D4, NET, Alpha1A, Sigma1, Sigma2, DOR, KOR, MOR, H1, M2, DAT, M4, M5, CB2, H2, CB1; **ND:** Beta2, Ca+Channel, Beta1

**cis-2a:** 4.00 5ht1a, 3.79 5ht1b, 3.46 D3, 3.30 5ht7, 3.25 5ht6, 3.15 5ht5a, **2.89 5ht2a**, 2.72 5ht2b, 2.67 D2, 2.49 D4, **2.34 5ht2c**, 2.07 5ht1e, 2.00 D1, 1.76 Beta1, 1.66 H1, 1.64 Alpha1A, 1.36 D5, 0.70 Beta2; **0.00:** DOR, SERT, MOR, KOR, NET, DAT, Alpha1B; **ND:** 5ht1d, Alpha2A, Sigma2, Alpha2B, Alpha2C, Imidazoline1, M1, M2, M3, M4, M5, Sigma1, H2, CB2, CB1, Ca+Channel, NMDA

**5-MeO-MIPT:** 4.00 5ht1a, 3.79 5ht7, 3.74 5ht1d, 3.32 5ht2b, 2.98 5ht6, 2.85 Alpha2A, 2.61 5ht1b, **2.44 5ht2a**, 2.29 Alpha2C, 2.15 Imidazoline1, 2.13 Sigma2, 2.11 5ht5a, 1.86 Alpha2B, **1.75 5ht2c**, 1.70 D3, 1.55 5ht1e, 1.41 H1, 1.29 D4, 1.28 SERT; **0.00:** D2, Alpha1B, D5, D1, Beta2, NET, DAT, Sigma1, Beta1, DOR, KOR, MOR, M1, M2, M3, M4, M5, Alpha1A, H2, CB2, NMDA, Ca+Channel; **ND:** CB1

**Psilocin:** 4.00 5ht2b, 3.40 5ht1d, 3.37 D1, 3.03 5ht1e, 2.88 5ht1a, 2.83 5ht5a, 2.82 5ht7, 2.82 5ht6, 2.67 D3, **2.52 5ht2c**, 2.19 5ht1b, **2.14 5ht2a**, 1.77 Imidazoline1, 1.74 SERT, 1.57 Alpha2B, 1.36 Alpha2A, 1.03 Alpha2C; **0.00:** D2, Alpha1B, D5, D4, Beta2, Beta1, DAT, NET, Alpha1A, Sigma1, Sigma2, DOR, KOR, MOR, M1, M2, M3, M4, Ca+Channel, H1, H2, CB2, CB1; **ND:** M5, NMDA

**DIPT:** 4.00 5ht1a, 3.53 Imidazoline1, 3.48 5ht2b, 2.98 SERT, 2.83 Sigma1, 2.68 Alpha2C, 2.65 Sigma2, 2.62 Alpha2B, 2.56 D3, 2.55 5ht7, 2.53 H1, 2.51 5ht1d; **0.00: 5ht2a**, D4, 5ht5a, D1, D2, Alpha2A, 5ht6, D5, Beta1, Beta2, **5ht2c**, DAT, NET, 5ht1b, Alpha1B, 5ht1e, DOR, KOR, MOR, M1, M2, M3, M4, M5, Alpha1A, H2, CB2, CB1, Ca+Channel, NMDA

**5-MeO-DIPT:** 4.00 5ht1a, 3.91 5ht2b, 3.24 Imidazoline1, 3.03 5ht7, 2.89 5ht1d, 2.72 SERT, 2.66 Alpha2C, 2.64 Sigma2, 2.41 5ht1b, 2.40 Alpha2B, 2.15 Sigma1; **0.00:** 5ht5a, **5ht2a**, D4, D3, D1, D2, Alpha2A, 5ht6, D5, Beta1, Beta2, **5ht2c**, DAT, NET, Alpha1A, Alpha1B, 5ht1e, DOR, KOR, MOR, M1, M2, M3, M4, M5, H1, H2, CB2, CB1, Ca+Channel, NMDA

**RR-2b:** 4.00 5ht1b, 3.59 5ht1a, 3.20 5ht5a, 3.05 5ht1d, 2.81 5ht7, 2.52 5ht6, 2.40 D3, 2.16 5ht1e, 2.08 D4, 1.88 D2, 1.81 5ht2b, 1.77 D1, **1.63 5ht2c**, 1.53 D5, **1.46 5ht2a**, 1.14 H1, 0.59 Alpha1A; **0.00:** SERT, DOR, KOR, MOR, Alpha1B, NET, DAT; **ND:** Alpha2A, Imidazoline1, Beta2, Sigma2, Alpha2B, Alpha2C, Beta1, M1, M2, M3, M4, M5, Sigma1, H2, CB2, CB1, Ca+Channel, NMDA

**2C-T-2:** 4.00 5ht2b, **3.18 5ht2a**, **3.05 5ht2c**, 2.84 5ht1d, 2.56 Alpha2C, 2.20 5ht1a, 2.16 5ht1e, 1.94 M3, 1.92 Alpha2A, 1.84 5ht1b, 1.79 Alpha2B, 1.79 5ht7, 1.70 Beta2, 1.64 5ht6, 1.60 M5, 1.51 D3, 1.46 Imidazoline1, 1.33 D2, 1.19 Sigma1, 0.81 Beta1; **0.00:** D1, 5ht5a, SERT, D4, NET, Alpha1A, Alpha1B, Sigma2, DOR, KOR, MOR, M1, M2, DAT, M4, D5, H1, H2, CB2, CB1, Ca+Channel, NMDA

**4C-T-2:** 4.00 5ht2b, 3.67 Beta2, **3.33 5ht2a**, **3.09 5ht2c**, 3.05 Sigma1, 2.79 Imidazoline1, 2.66 D3, 2.56 5ht5a, 2.18 5ht7, 2.04 5ht1a, 1.77 5ht1e; **0.00:** 5ht6, D1, D4, D5, Alpha1A, Alpha1B, Alpha2A, Alpha2B, Alpha2C, Beta1, D2, SERT, DAT, NET, 5ht1b, 5ht1d, Sigma2, DOR, KOR, MOR, M1, M2, M3, M4, M5, H1, H2, CB2, CB1, Ca+Channel, NMDA

**MDMA:** 4.00 Imidazoline1, 3.64 5ht2b, 3.26 Ca+Channel, 3.21 Alpha2C, 3.09 Alpha2B, 3.07 M3, 2.94 Alpha2A, 2.54 M5, 2.43 M4; **0.00: 5ht2c**, 5ht1d, D2, 5ht1e, 5ht1a, **5ht2a**, Alpha1A, Alpha1B, 5ht5a, 5ht6, 5ht7, D1, Beta2, SERT, DAT, NET, 5ht1b, H1, H2, D3, KOR, Beta1, M1, M2, D5, D4, CB1, NMDA, MOR; **ND:** DOR, Sigma2, CB2, Sigma1

**Ibogaine:** 4.00 Sigma2, 3.57 SERT, 3.02 DAT, 3.01 NMDA, 2.88 KOR, 2.67 MOR, 2.55 Sigma1, 2.22 M3, **2.16 5ht2a**, 1.96 M1, 1.72 M2, 1.47 D3; **0.00:** DOR, 5ht1b, 5ht1d, 5ht1a, H1, **5ht2c**, D2, D1, Beta1; **ND:** Alpha2C, D5, D4, Alpha2B, Imidazoline1, NET, Alpha2A, 5ht5a, 5ht6, 5ht7, Alpha1B, 5ht1e, 5ht2b, M4, M5, Alpha1A, H2, CB2, CB1, Ca+Channel, Beta2

**DOET:** 4.00 5ht1a, **3.72 5ht2a**, 3.70 5ht2b, **3.13 5ht2c**, 2.40 Alpha2B, 2.07 5ht7, 2.05 Alpha2A, 2.00 Alpha2C, 1.82 Beta2, 1.71 5ht1b, 1.61 5ht1e, 1.40 Beta1, 1.34 5ht1d, 1.18 Sigma2, 1.17 Sigma1; **0.00:** D4, Alpha1A, D3, 5ht6, D5, D1, D2, SERT, DAT, NET, Imidazoline1, Alpha1B, 5ht5a, DOR, KOR, MOR, NMDA, M2, CB2, CB1, M5, H1, H2; **ND:** M3, M4, Ca+Channel, M1

**5-MeO-DMT:** 4.00 5ht1a, 3.69 5ht7, 3.48 5ht1d, 2.73 5ht6, 2.41 5ht1b, 2.38 D1, 1.84 5ht5a, 1.72 5ht1e, 1.58 D3, 1.57 Alpha2C, **1.55 5ht2c**, 1.00 Alpha2A, **0.98 5ht2a**, 0.97 SERT, 0.88 Imidazoline1, 0.86 Alpha2B, 0.82 NET, 0.78 D4, 0.73 D2, 0.69 5ht2b; **0.00:** Alpha1B, Beta2, Beta1, DAT, D5, Alpha1A, Sigma1, Sigma2, CB2, KOR, Ca+Channel, M1, M2, M3, M4, M5, H2, CB1; **ND:** H1, DOR, MOR, NMDA

**SS-2c:** 4.00 5ht1a, 3.22 5ht1b, 2.82 D3, 2.45 5ht7, 2.44 5ht6, **2.32 5ht2a**, 2.17 5ht5a, 2.17 5ht2b, **2.03 5ht2c**, 1.74 D2, 1.72 Beta1, 1.62 D4, 1.16 5ht1e, 1.14 D1, 1.00 D5, 0.67 Alpha1A, 0.57 Beta2, 0.20 H1; **0.00:** DOR, SERT, MOR, KOR, NET, DAT, Alpha1B; **ND:** 5ht1d, Alpha2A, Sigma2, Alpha2B, Alpha2C, Imidazoline1, M1, M2, M3, M4, M5, Sigma1, H2, CB2, CB1, Ca+Channel, NMDA

**2C-B-fly:** 4.00 5ht2b, 3.81 5ht1d, **2.93 5ht2c**, **2.89 5ht2a**, 1.91 5ht1e, 1.79 5ht1a, 1.79 Alpha2A, 1.78 5ht6, 1.69 5ht1b, 1.59 Alpha2C, 1.42 M3, 1.24 M4, 1.17 5ht7, 1.16 Alpha2B, 1.15 M1, 1.01 M5, 0.65 M2, 0.26 D1, 0.19 H1, 0.12 D2; **0.00:** Beta1, Alpha1B, 5ht5a, DAT, NET, Imidazoline1, Sigma1, Sigma2, DOR, KOR, MOR, Beta2, SERT, CB2, D4, D5, Alpha1A, H2, CB1; **ND:** D3, Ca+Channel, NMDA

**Mescaline:** 4.00 Alpha2C, 3.97 5ht2b, 3.61 5ht1a, 3.44 Imidazoline1, 3.16 5ht1e, 2.92 Alpha2A; **0.00: 5ht2a**, **5ht2c**, 5ht6, 5ht1d, D1, D2, D3, D4, D5, Alpha1A, Alpha1B, 5ht5a, Alpha2B, 5ht7, Beta1, Beta2, SERT, DAT, NET, 5ht1b, Sigma1, Sigma2, DOR, KOR, MOR, M1, M2, M3, M4, M5, H1, H2, CB2, CB1, Ca+Channel, NMDA

**DOB:** 4.00 5ht2b, **3.23 5ht2a**, **2.97 5ht2c**, 2.11 Beta2, 1.89 5ht7, 1.82 Alpha2C, 1.79 5ht1d, 1.68 D3, 1.62 5ht1b, 1.53 M3, 1.44 5ht1e, 1.41 Alpha2B, 1.39 Imidazoline1, 1.25 Sigma1, 1.21 Beta1, 1.18 5ht1a, 0.96 Alpha2A, 0.87 5ht5a, 0.85 5ht6, 0.66 SERT, 0.63 H1; **0.00:** D5, D2, D4, NET, D1, Alpha1B, Sigma2, DOR, KOR, MOR, M1, M2, DAT, M4, M5, Alpha1A, H2, CB2, CB1, Ca+Channel, NMDA

**5-MeO-TMT:** 4.00 5ht6, 3.62 5ht7, 3.48 5ht1a, 3.38 5ht1d, 2.52 5ht1e, **2.17 5ht2c**, 1.97 NET, 1.76 5ht5a; **0.00: 5ht2a**, DAT, D1, D2, D3, D4, D5, H1, H2, SERT, DOR, KOR, MOR; **ND:** Beta2, Alpha2A, 5ht2b, 5ht1b, Imidazoline1, Sigma1, Sigma2, Alpha2B, Alpha2C, Beta1, M1, M2, M3, M4, M5, Alpha1A, Alpha1B, CB2, CB1, Ca+Channel, NMDA

**DOM:** 4.00 5ht2b, 3.38 Beta2, 2.75 5ht1d, **2.36 5ht2a**, 2.30 Alpha2A, 2.13 Alpha2B, 2.10 Alpha2C, 1.87 5ht7, 1.56 Alpha1A, 1.52 5ht1e, 1.51 5ht1a, **1.47 5ht2c**, 1.16 5ht6; **0.00:** D3, 5ht1b, D1, Alpha1B, 5ht5a, D4, D5, Beta1, D2, SERT, DAT, NET, Imidazoline1, Sigma1, Sigma2, DOR, KOR, MOR, M1, M2, CB2, CB1, M5, H1, H2, NMDA; **ND:** M3, Ca+Channel, M4

**MDA:** 4.00 5ht2b, 3.60 Alpha2C, 3.12 Alpha2B, 2.74 Alpha2A, 2.41 5ht7, 2.38 5ht1a, **2.15 5ht2c**; **0.00: 5ht2a**, 5ht1e, 5ht1d, D1, D2, D3, D4, 5ht1b, Alpha1A, Alpha1B, 5ht5a, 5ht6, D5, Beta1, Beta2, SERT, DAT, NET, Imidazoline1, CB2, H2, M5, KOR, M2, CB1; **ND:** M1, M3, Sigma1, MOR, H1, Sigma2, DOR, M4, Ca+Channel, NMDA

**Aleph-2:** 4.00 5ht2b, 2.79 Beta2, **2.50 5ht2c**, **2.42 5ht2a**, 1.83 Sigma1, 1.70 Imidazoline1, 1.41 D3, 1.08 5ht7, 1.08 SERT, 1.06 Alpha2C, 1.02 5ht1d, 0.98 5ht1a, 0.92 M3, 0.90 5ht1b, 0.74 Alpha2B, 0.72 5ht6, 0.71 5ht1e, 0.44 Alpha2A, 0.37 Beta1, 0.30 M5; **0.00:** D1, D2, 5ht5a, D4, NET, Alpha1A, Alpha1B, Sigma2, DOR, KOR, MOR, M1, M2, DAT, M4, D5, H1, H2, CB2, CB1, Ca+Channel, NMDA

**EMDT:** 4.00 5ht6, 2.97 5ht1a, 2.74 5ht1d, 2.73 5ht7, 2.49 5ht1e, **1.95 5ht2c**, 1.54 5ht5a; **0.00: 5ht2a**, DAT, D5, D1, D2, D3, D4, NET, H1, H2, SERT, DOR, KOR, MOR; **ND:** Beta2, Alpha2A, 5ht2b, 5ht1b, Imidazoline1, Sigma1, Sigma2, Alpha2B, Alpha2C, Beta1, M1, M2, M3, M4, M5, Alpha1A, Alpha1B, CB2, CB1, Ca+Channel, NMDA

**TMA-2:** 4.00 5ht2b, **3.42 5ht2a**, 3.04 H1, **2.58 5ht2c**; **0.00:** 5ht1b, 5ht1a, 5ht1e, 5ht5a, 5ht6, 5ht7, D1, D2, D3, D4, D5, 5ht1d, Alpha1B, Alpha2A, Alpha2B, Alpha2C, Beta1, Beta2, SERT, DAT, NET, M5, Alpha1A, H2, M2; **ND:** KOR, DOR, M1, MOR, M3, M4, Imidazoline1, Sigma1, Sigma2, CB2, CB1, Ca+Channel, NMDA

**THC:** 4.00 CB1, 3.78 CB2; **ND: 5ht2a**, **5ht2c**, 5ht1b, 5ht1d, 5ht1e, 5ht2b, 5ht1a, 5ht7, D1, D2, D3, D4, D5, Alpha1A, Alpha1B, 5ht5a, 5ht6, Alpha2C, Beta1, Beta2, SERT, DAT, NET, Imidazoline1, Sigma1, Sigma2, DOR, KOR, MOR, M1, M2, M3, M4, M5, H1, H2, Alpha2A, Alpha2B, Ca+Channel, NMDA

**MEM:** 4.00 5ht2b, **2.21 5ht2a**, 2.10 Sigma1, 1.95 5ht7; **0.00: 5ht2c**, 5ht1a, 5ht1e, 5ht5a, 5ht6, 5ht1b, D1, D2, D3, D4, D5, Alpha1A, Alpha1B, Alpha2A, Alpha2B, Alpha2C, Beta1, Beta2, SERT, DAT, NET, Imidazoline1, 5ht1d, Sigma2, DOR, KOR, MOR, M1, M2, M3, M4, M5, H1, H2, CB2, CB1, Ca+Channel, NMDA

**Morphine:** 4.00 MOR, 2.21 KOR, 0.72 DOR; **ND: 5ht2c**, **5ht2a**, 5ht1d, 5ht1e, 5ht2b, 5ht1a, 5ht1b, D1, D2, D3, D4, D5, Alpha1A, Alpha1B, Alpha2A, Alpha2B, Alpha2C, Beta1, Beta2, SERT, DAT, NET, Imidazoline1, Sigma1, Sigma2, 5ht5a, 5ht6, 5ht7, M1, M2, M3, M4, M5, H1, H2, CB2, CB1, Ca+Channel, NMDA

**Salvinorin A:** 4.00 KOR; **0.00: 5ht2a**, 5ht2b, **5ht2c**, 5ht1b, 5ht1d, 5ht1e, 5ht5a, 5ht1a, 5ht7, D1, D2, D3, D4, D5, Alpha1A, Alpha1B, SERT, DOR, 5ht6, Beta1, Beta2, M2, DAT, M4, M5, H1, M1, M3, MOR; **ND:** Alpha2A, Alpha2C, Sigma2, Alpha2B, NET, Imidazoline1, Sigma1, H2, CB2, CB1, Ca+Channel, NMDA

### Groups of Related Receptors

In addition to looking at breadth at the full complement of forty-two receptors with which the drugs interact, we can use the B_sq_ statistic to look at the participation of selected groups of receptors (Table S5). 5-HT_6_ and 5-HT_7_ are grouped because they share a common G-protein, G_s_ [13]. The same data is also presented in Table S6, which also includes interactions with individual receptors. Tables S5 and S6 allow us to easily identify drugs with the greatest or least interaction with an individual receptor or any group of related receptors.

For example, among drugs with measurable affinity at a 5-HT_2_ receptor, 5-MeO-DMT has the weakest interaction with both the three 5-HT_2_ receptors and the two paradigmatic 5-HT_2_ receptors (5-HT_2A/C_). This can be seen in that 5-MeO-DMT has the lowest non-zero value in all four of the columns: “5-HT_2_,” “5-HT_2_max,” “5-HT_2A_, 5-HT_2C_,” and “5-HT_2A/C_max.” Meanwhile, the drugs 2C-E and DOI rise to the top of the same four columns, indicating that they have the strongest interactions with the 5-HT_2_ receptors (fourteen drugs tie for the top value in the 5-HT_2_max column because thirteen drugs have 5-HT_2B_ as their best hit and one has 5-HT_2C_ as its best hit). LSD has the strongest interaction collectively with the five dopamine receptors (D_1_, D_2_, D_3_, D_4_, D_5_), the ten assayed 5-HT receptors, and the four assayed 5-HT_1_ (serotonin-1) receptors. DMT has the strongest interaction with any single dopamine receptor (Dmax), and is the only drug to have its best hit at the 5-HT_7_ receptor (Table S6). Mescaline is the only drug to have its best hit at an adrenergic receptor (α_2C_, Table S6). Ibogaine is the only drug to have its best hit at a sigma receptor. MDMA is the only drug to have its best hit at an imidazoline receptor (Table S6). These observations likely provide clues to the qualitative diversity of these drugs.

### Proportional Breadth

There is yet another way to look at the participation of the sub-sets of receptors. It may be relevant to consider the participation of a sub-set of receptors in proportion to the participation of all receptors (Table S7). For this we use the proportional breadth statistic (B_p_) described in the methods section. The same data is also displayed in Table S8.

The proportional breadth statistic, B_p_, is strongly influenced by the degree of overall breadth of the drug (B_sq_^2^), as this determines the denominator in calculating the proportion. Therefore, we find MEM at the top of the 5-HT column because it is almost completely selective for a single receptor, 5-HT_2B_. Similarly, TMA-2 is at the top of the 5-HT_2_ columns because it is highly selective for the 5-HT_2_ receptors, and EMDT is at the top of the “5-HT_6_, 5-HT_7_” column because it is highly selective for the 5-HT_6_ receptor. Due to their high degree of selectivity, MEM, TMA-2, and EMDT all have small denominators in calculating the B_p_ statistic.

More interesting cases involve less selective drugs. For example, DMT is a very promiscuous drug, yet it falls at the top of the “α_1A_, α_1B_” column. MDA falls at the top of the “Adrenergic” and “α_2A_, α_2B_, α_2C_” columns. 5-MeO-DMT appears third from the top of the “5-HT_6_, 5-HT_7_” column and second from the top of the “5-HT_7_” column (Table S8). Ibogaine appears at the top of the “σ_1_, σ_2_” column. Although fairly selective, TMA-2's position at the top of the “H_1_, H_2_” column represents an important aspect of its pharmacology, likewise for DOM's position at the top of the “β_1_, β_2_” column.

### Profiles of Receptors

**Figure 5:**
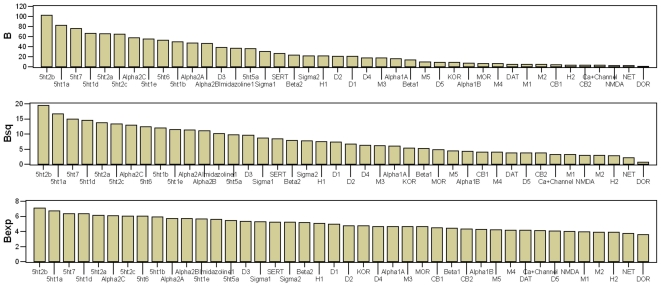
Forty-two receptors arranged in order of decreasing interaction with the full set of thirty-five drugs. The forty-two receptors are arranged in order of decreasing interaction with the full set of thirty-five drugs, based on the breadth statistics, B, B_sq_. and B_exp_. The receptors with the greatest interactions are found at the left of the figures, and the receptors with the least interactions are found at the right of the figures. The black vertical bars represent a 100-fold drop in affinity relative to the receptor with the highest affinity at each drug. As a rule of thumb, this is presumed to be the limit of perceptible receptor interaction. Drugs to the right of the black bar should have imperceptible interactions with the receptor, while drugs to the left of the black bar should have perceptible interactions with the receptor, increasingly so the further left they are.

The B statistics can also be used to look at the relative role played by the various receptors in the pharmacology of the entire set of drugs of this study. In this case, for each receptor, we sum the npK_i_ values at each receptor across each of the thirty-five drugs. In Table 5 we can see the rankings from the three B statistics. The data in Table 5 can also be represented graphically (Fig. 5). The three B statistics provide a very consistent ranking for the top seven receptors. In descending order of importance: 5-HT_2B_, 5-HT_1A_, 5-HT_7_, 5-HT_1D_, 5-HT_2A_, 5-HT_2C_, α_2C_ (with some play between the sixth and seventh positions). This set of top receptors would be a good place to look for receptors other than 5-HT_2A_ and 5-HT_2C_, which play an important role in the actions of psychedelic drugs.

**Table 5:** Forty-two receptors arranged in order of decreasing interaction with the full set of thirty-five drugs.

B	Receptor	B_sq_	Receptor	B_exp_	Receptor
102.400	5ht2b	19.479	5ht2b	7.094	5ht2b
82.564	5ht1a	16.634	5ht1a	6.709	5ht1a
75.736	5ht7	14.931	5ht7	6.348	5ht7
66.217	5ht1d	14.574	5ht1d	6.348	5ht1d
65.462	5ht2a	13.806	5ht2a	6.153	5ht2a
64.798	5ht2c	13.352	5ht2c	6.071	Alpha2C
58.020	Alpha2C	12.989	Alpha2C	6.037	5ht2c
55.055	5ht1e	12.430	5ht6	6.034	5ht6
53.236	5ht6	11.951	5ht1b	5.930	5ht1b
49.634	5ht1b	11.508	5ht1e	5.722	Alpha2A
47.526	Alpha2A	11.304	Alpha2A	5.709	Alpha2B
46.760	Alpha2B	11.138	Alpha2B	5.666	5ht1e
38.823	D3	10.097	Imidazoline1	5.608	Imidazoline1
36.702	Imidazoline1	9.741	5ht5a	5.450	5ht5a
36.079	5ht5a	9.561	D3	5.348	D3
30.487	Sigma1	8.657	Sigma1	5.271	Sigma1
26.764	SERT	8.448	SERT	5.252	SERT
23.237	Beta2	7.831	Beta2	5.248	Sigma2
21.839	Sigma2	7.808	Sigma2	5.170	Beta2
21.769	H1	7.452	H1	5.080	H1
21.302	D2	7.296	D1	4.985	D1
21.107	D1	6.697	D2	4.732	D2
17.995	D4	6.278	D4	4.732	KOR
17.821	M3	6.205	M3	4.649	D4
16.510	Alpha1A	6.043	Alpha1A	4.644	Alpha1A
14.117	Beta1	5.401	KOR	4.643	M3
9.935	M5	5.209	Beta1	4.626	MOR
9.137	D5	4.812	MOR	4.484	CB1
9.089	KOR	4.492	M5	4.425	Beta1
7.540	Alpha1B	4.297	Alpha1B	4.355	CB2
6.674	MOR	4.000	CB1	4.282	Alpha1B
6.563	M4	3.978	M4	4.243	M5
5.344	DAT	3.810	DAT	4.173	M4
5.334	M1	3.809	D5	4.154	DAT
4.727	M2	3.782	CB2	4.097	Ca+Channel
4.000	CB1	3.263	Ca+Channel	4.059	D5
3.861	H2	3.181	M1	3.995	NMDA
3.783	CB2	3.013	NMDA	3.942	M1
3.263	Ca+Channel	2.992	M2	3.914	M2
3.013	NMDA	2.824	H2	3.884	H2
2.796	NET	2.138	NET	3.749	NET
0.720	DOR	0.720	DOR	3.585	DOR

The forty-two receptors are arranged in order of decreasing interaction with the full set of thirty-five drugs, based on the breadth statistics B, B_sq_. and B_exp_. The receptors with the greatest interactions are found at the tops of the columns, and the receptors with the least interactions are found at the bottoms of the columns.

Fig. S3 is a more detailed graphical view of data presented in Table 5. The receptors are presented in order of decreasing breadth (B_sq_). The figures for each individual receptor provide information on the relative importance of each receptor at each drug, similar to that in Table S5 and Table S6. Receptors at the top of the figure have the broadest interactions with the thirty-five drugs, while receptors at the bottom of the figure have the narrowest interactions with the thirty-five drugs. The black vertical bars represent a 100-fold drop in affinity relative to the receptor with the highest affinity at each drug. As a rule of thumb, this is presumed to be the limit of perceptible receptor interaction. Drugs to the right of the black bar should have imperceptible interactions with the receptor, while drugs to the left of the black bar should have perceptible interactions with the receptor, increasingly so the further left they are. It is also useful to present the npK_i_ data of Fig. S3 in numerical format. npK_i_ values below about 2.0 should be imperceptible, while values above about 2.0 should be perceptible, and the higher the npK_i_ value, the more perceptible a receptor should be at a particular drug.

**5ht2b:** 4.00 DOB, 4.00 MDA, 4.00 Aleph-2, 4.00 2C-B-fly, 4.00 2C-B, 4.00 TMA, 4.00 psilocin, 4.00 TMA-2, 4.00 2C-E, 4.00 2C-T-2, 4.00 4C-T-2, 4.00 MEM, 4.00 DOM, 3.97 mescaline, 3.93 6-F-DMT, 3.91 5-MeO-DIPT, 3.91 DMT, 3.88 DPT, 3.70 DOET, 3.64 MDMA, 3.48 DIPT, 3.32 5-MeO-MIPT, 3.13 DOI, 3.11 LSD, 3.01 lisuride, 2.72 cis-2a, 2.17 SS-2c, 1.81 RR-2b, 0.69 5-MeO-DMT; 0.00 salvinorin A; **ND:** 5-MeO-TMT, ibogaine, EMDT, morphine, THC

**5ht1a:** 4.00 5-MeO-MIPT, 4.00 lisuride, 4.00 DOET, 4.00 SS-2c, 4.00 5-MeO-DIPT, 4.00 DIPT, 4.00 5-MeO-DMT, 4.00 cis-2a, 4.00 DPT, 3.73 LSD, 3.61 mescaline, 3.59 RR-2b, 3.48 5-MeO-TMT, 3.45 TMA, 2.97 EMDT, 2.91 2C-E, 2.88 psilocin, 2.81 6-F-DMT, 2.75 2C-B, 2.38 MDA, 2.31 DOI, 2.20 2C-T-2, 2.04 4C-T-2, 1.79 2C-B-fly, 1.51 DOM, 1.18 DOB, 0.98 Aleph-2; **0.00:** TMA-2, MEM, MDMA, DMT, ibogaine, salvinorin A; **ND:** morphine, THC

**5ht7:** 4.00 DMT, 3.80 6-F-DMT, 3.80 TMA, 3.79 5-MeO-MIPT, 3.77 LSD, 3.69 5-MeO-DMT, 3.62 5-MeO-TMT, 3.30 cis-2a, 3.05 DPT, 3.03 5-MeO-DIPT, 2.82 psilocin, 2.81 2C-B, 2.81 RR-2b, 2.77 2C-E, 2.73 EMDT, 2.64 lisuride, 2.55 DIPT, 2.45 SS-2c, 2.41 MDA, 2.18 4C-T-2, 2.07 DOET, 1.95 MEM, 1.90 DOI, 1.89 DOB, 1.87 DOM, 1.79 2C-T-2, 1.17 2C-B-fly, 1.08 Aleph-2; **0.00:** TMA-2, MDMA, mescaline, salvinorin A; **ND:** ibogaine, morphine, THC

**5ht1d:** 3.97 DMT, 3.81 2C-B-fly, 3.74 5-MeO-MIPT, 3.71 2C-B, 3.70 LSD, 3.66 6-F-DMT, 3.54 2C-E, 3.48 5-MeO-DMT, 3.40 psilocin, 3.38 5-MeO-TMT, 3.20 TMA, 3.05 RR-2b, 3.00 DOI, 2.89 5-MeO-DIPT, 2.84 2C-T-2, 2.75 DOM, 2.74 EMDT, 2.71 DPT, 2.51 DIPT, 1.79 DOB, 1.34 DOET, 1.02 Aleph-2; **0.00:** 4C-T-2, mescaline, TMA-2, MEM, lisuride, MDMA, salvinorin A, MDA, ibogaine; **ND:** cis-2a, SS-2c, morphine, THC

**5ht2a:** 3.76 2C-E, 3.72 DOET, 3.69 2C-B, 3.54 LSD, 3.44 DOI, 3.42 TMA-2, 3.33 4C-T-2, 3.23 DOB, 3.18 2C-T-2, 2.89 2C-B-fly, 2.89 cis-2a, 2.74 lisuride, 2.58 DMT, 2.47 6-F-DMT, 2.44 5-MeO-MIPT, 2.42 Aleph-2, 2.36 DOM, 2.32 SS-2c, 2.21 MEM, 2.16 ibogaine, 2.14 psilocin, 2.09 DPT, 1.46 RR-2b, 0.98 5-MeO-DMT; **0.00:** TMA, DIPT, MDA, MDMA, mescaline, 5-MeO-TMT, EMDT, 5-MeO-DIPT, salvinorin A; **ND:** morphine, THC

**5ht2c:** 4.00 DOI, 3.42 DMT, 3.38 2C-E, 3.18 2C-B, 3.13 DOET, 3.11 LSD, 3.09 4C-T-2, 3.05 2C-T-2, 3.02 TMA, 2.97 DOB, 2.93 2C-B-fly, 2.58 TMA-2, 2.58 6-F-DMT, 2.52 psilocin, 2.50 Aleph-2, 2.34 cis-2a, 2.31 DPT, 2.17 5-MeO-TMT, 2.15 MDA, 2.03 SS-2c, 1.95 EMDT, 1.75 5-MeO-MIPT, 1.63 RR-2b, 1.55 5-MeO-DMT, 1.47 DOM; **0.00:** 5-MeO-DIPT, mescaline, lisuride, MEM, MDMA, DIPT, ibogaine, salvinorin A; **ND:** morphine, THC

**Alpha2C:** 4.00 mescaline, 3.88 lisuride, 3.60 MDA, 3.53 DMT, 3.44 2C-E, 3.24 6-F-DMT, 3.21 MDMA, 3.12 2C-B, 2.98 TMA, 2.88 DOI, 2.79 DPT, 2.68 DIPT, 2.66 5-MeO-DIPT, 2.56 2C-T-2, 2.29 5-MeO-MIPT, 2.10 DOM, 2.00 DOET, 1.82 DOB, 1.59 2C-B-fly, 1.57 5-MeO-DMT, 1.06 Aleph-2, 1.03 psilocin; **0.00:** 4C-T-2, MEM, TMA-2; **ND:** LSD, cis-2a, RR-2b, SS-2c, 5-MeO-TMT, EMDT, ibogaine, salvinorin A, morphine, THC

**5ht6:** 4.00 6-F-DMT, 4.00 EMDT, 4.00 5-MeO-TMT, 3.75 LSD, 3.25 cis-2a, 3.25 DMT, 2.98 5-MeO-MIPT, 2.82 psilocin, 2.73 5-MeO-DMT, 2.63 2C-B, 2.61 lisuride, 2.52 RR-2b, 2.44 SS-2c, 2.34 DOI, 1.93 2C-E, 1.85 DPT, 1.78 2C-B-fly, 1.64 2C-T-2, 1.16 DOM, 0.85 DOB, 0.72 Aleph-2; **0.00:** 4C-T-2, 5-MeO-DIPT, mescaline, TMA, TMA-2, MDA, DOET, MEM, MDMA, DIPT, salvinorin A; **ND:** ibogaine, morphine, THC

**5ht1b:** 4.00 LSD, 4.00 RR-2b, 3.79 cis-2a, 3.22 SS-2c, 3.22 TMA, 3.11 2C-B, 3.07 6-F-DMT, 3.00 2C-E, 2.61 5-MeO-MIPT, 2.57 DPT, 2.41 5-MeO-DIPT, 2.41 5-MeO-DMT, 2.27 lisuride, 2.19 psilocin, 1.84 2C-T-2, 1.71 DOET, 1.69 2C-B-fly, 1.62 DOB, 0.90 Aleph-2; **0.00:** 4C-T-2, MDMA, mescaline, DMT, DIPT, TMA-2, DOM, MDA, DOI, MEM, ibogaine, salvinorin A; **ND:** 5-MeO-TMT, EMDT, morphine, THC

**5ht1e:** 3.28 DMT, 3.16 mescaline, 3.15 TMA, 3.05 2C-B, 3.03 psilocin, 2.74 6-F-DMT, 2.66 DOI, 2.62 LSD, 2.60 2C-E, 2.52 5-MeO-TMT, 2.49 EMDT, 2.16 2C-T-2, 2.16 RR-2b, 2.13 DPT, 2.07 cis-2a, 1.91 2C-B-fly, 1.83 lisuride, 1.77 4C-T-2, 1.72 5-MeO-DMT, 1.61 DOET, 1.55 5-MeO-MIPT, 1.52 DOM, 1.44 DOB, 1.16 SS-2c, 0.71 Aleph-2; **0.00:** 5-MeO-DIPT, TMA-2, MDA, MEM, MDMA, DIPT, salvinorin A; **ND:** ibogaine, morphine, THC

**Alpha2A:** 3.79 DOI, 3.36 TMA, 3.22 lisuride, 2.94 MDMA, 2.93 LSD, 2.92 mescaline, 2.85 5-MeO-MIPT, 2.84 DPT, 2.75 DMT, 2.74 MDA, 2.71 2C-E, 2.64 2C-B, 2.30 DOM, 2.05 DOET, 1.92 2C-T-2, 1.79 2C-B-fly, 1.36 psilocin, 1.00 5-MeO-DMT, 0.96 DOB, 0.44 Aleph-2; **0.00:** 4C-T-2, DIPT, 5-MeO-DIPT, MEM, TMA-2, 6-F-DMT; **ND:** cis-2a, RR-2b, SS-2c, 5-MeO-TMT, EMDT, ibogaine, salvinorin A, morphine, THC

**Alpha2B:** 3.78 lisuride, 3.53 DMT, 3.13 DOI, 3.12 MDA, 3.09 MDMA, 2.99 6-F-DMT, 2.97 DPT, 2.91 2C-E, 2.62 DIPT, 2.40 DOET, 2.40 5-MeO-DIPT, 2.31 2C-B, 2.13 DOM, 1.86 5-MeO-MIPT, 1.79 2C-T-2, 1.57 psilocin, 1.41 DOB, 1.16 2C-B-fly, 0.86 5-MeO-DMT, 0.74 Aleph-2; **0.00:** 4C-T-2, mescaline, TMA, MEM, TMA-2; **ND:** LSD, cis-2a, RR-2b, SS-2c, 5-MeO-TMT, EMDT, ibogaine, salvinorin A, morphine, THC

**Imidazoline1:** 4.00 MDMA, 3.53 DIPT, 3.44 mescaline, 3.24 5-MeO-DIPT, 3.13 DMT, 2.97 DPT, 2.79 4C-T-2, 2.45 6-F-DMT, 2.15 5-MeO-MIPT, 1.80 2C-B, 1.77 psilocin, 1.70 Aleph-2, 1.46 2C-T-2, 1.39 DOB, 0.88 5-MeO-DMT; **0.00:** MEM, 2C-E, MDA, lisuride, DOI, 2C-B-fly, DOM, TMA, DOET; **ND:** TMA-2, LSD, cis-2a, RR-2b, SS-2c, 5-MeO-TMT, EMDT, ibogaine, salvinorin A, morphine, THC

**5ht5a:** 3.64 LSD, 3.20 RR-2b, 3.16 DMT, 3.15 cis-2a, 2.99 lisuride, 2.83 psilocin, 2.56 4C-T-2, 2.43 6-F-DMT, 2.17 SS-2c, 2.11 5-MeO-MIPT, 1.86 DPT, 1.84 5-MeO-DMT, 1.76 5-MeO-TMT, 1.54 EMDT, 0.87 DOB; **0.00:** MDMA, 5-MeO-DIPT, 2C-B, MDA, mescaline, 2C-B-fly, DOI, TMA, DOET, TMA-2, 2C-E, 2C-T-2, Aleph-2, MEM, DOM, DIPT, salvinorin A; **ND:** ibogaine, morphine, THC

**D3:** 3.46 cis-2a, 3.16 LSD, 2.82 SS-2c, 2.67 psilocin, 2.66 4C-T-2, 2.56 DIPT, 2.47 6-F-DMT, 2.40 RR-2b, 2.37 DPT, 2.27 2C-E, 1.70 5-MeO-MIPT, 1.68 DOB, 1.58 5-MeO-DMT, 1.51 2C-T-2, 1.47 ibogaine, 1.41 Aleph-2, 1.34 lisuride, 1.28 2C-B; **0.00:** MDMA, mescaline, MEM, DMT, TMA, DOET, TMA-2, DOM, MDA, DOI, salvinorin A, 5-MeO-TMT, EMDT, 5-MeO-DIPT; **ND:** 2C-B-fly, morphine, THC

**Sigma1:** 3.95 TMA, 3.05 4C-T-2, 2.90 DPT, 2.83 DIPT, 2.55 ibogaine, 2.23 DMT, 2.15 5-MeO-DIPT, 2.10 MEM, 1.83 Aleph-2, 1.73 DOI, 1.57 6-F-DMT, 1.25 DOB, 1.19 2C-T-2, 1.17 DOET; **0.00:** 2C-B, mescaline, 5-MeO-MIPT, DOM, 2C-B-fly, 5-MeO-DMT, psilocin; **ND:** 2C-E, lisuride, MDA, TMA-2, LSD, cis-2a, MDMA, SS-2c, 5-MeO-TMT, EMDT, RR-2b, salvinorin A, morphine, THC

**SERT:** 3.57 ibogaine, 3.31 DPT, 3.25 6-F-DMT, 2.98 DIPT, 2.83 DOI, 2.72 5-MeO-DIPT, 2.37 DMT, 1.74 psilocin, 1.28 5-MeO-MIPT, 1.08 Aleph-2, 0.97 5-MeO-DMT, 0.66 DOB; **0.00:** 2C-E, MDA, 2C-B, mescaline, 4C-T-2, 2C-T-2, lisuride, MEM, 2C-B-fly, DOM, TMA, MDMA, TMA-2, LSD, cis-2a, RR-2b, SS-2c, 5-MeO-TMT, EMDT, DOET, salvinorin A; **ND:** morphine, THC

**Beta2:** 3.67 4C-T-2, 3.52 DOI, 3.38 DOM, 2.79 Aleph-2, 2.11 DOB, 1.93 lisuride, 1.82 DOET, 1.70 2C-T-2, 1.05 LSD, 0.70 cis-2a, 0.57 SS-2c; **0.00:** 5-MeO-DMT, DMT, mescaline, DIPT, DPT, 5-MeO-MIPT, 6-F-DMT, 5-MeO-DIPT, MDMA, 2C-B-fly, psilocin, TMA, TMA-2, MDA, MEM, salvinorin A; **ND:** 2C-B, 2C-E, RR-2b, 5-MeO-TMT, ibogaine, EMDT, morphine, THC

**Sigma2:** 4.00 ibogaine, 3.95 TMA, 2.65 DIPT, 2.64 5-MeO-DIPT, 2.13 5-MeO-MIPT, 2.04 DPT, 1.70 DOI, 1.56 6-F-DMT, 1.18 DOET; **0.00:** 2C-T-2, 2C-E, mescaline, 2C-B, DOB, Aleph-2, MEM, 4C-T-2, DOM, DMT, 5-MeO-DMT, 2C-B-fly, psilocin; **ND:** lisuride, MDA, TMA-2, LSD, cis-2a, MDMA, SS-2c, 5-MeO-TMT, EMDT, RR-2b, salvinorin A, morphine, THC

**H1:** 3.74 6-F-DMT, 3.41 DPT, 3.04 TMA-2, 2.53 DIPT, 2.42 DOI, 1.66 cis-2a, 1.41 5-MeO-MIPT, 1.40 LSD, 1.14 RR-2b, 0.63 DOB, 0.20 SS-2c, 0.19 2C-B-fly; **0.00:** psilocin, 2C-B, 5-MeO-DIPT, TMA, 4C-T-2, 2C-E, 2C-T-2, MDMA, mescaline, DOM, EMDT, DOET, salvinorin A, 5-MeO-TMT, MEM, Aleph-2, ibogaine; **ND:** lisuride, DMT, MDA, 5-MeO-DMT, morphine, THC

**D1:** 3.51 DMT, 3.37 psilocin, 2.67 6-F-DMT, 2.38 5-MeO-DMT, 2.34 LSD, 2.00 cis-2a, 1.77 RR-2b, 1.67 DOI, 1.14 SS-2c, 0.26 2C-B-fly; **0.00:** 2C-B, MDA, 5-MeO-MIPT, DIPT, 5-MeO-DIPT, DPT, 4C-T-2, DOB, lisuride, MDMA, mescaline, DOM, TMA, DOET, TMA-2, 2C-E, 2C-T-2, Aleph-2, MEM, 5-MeO-TMT, EMDT, ibogaine, salvinorin A; **ND:** morphine, THC

**D2:** 2.67 cis-2a, 2.65 lisuride, 2.62 6-F-DMT, 2.55 LSD, 1.88 RR-2b, 1.88 2C-E, 1.74 SS-2c, 1.60 2C-B, 1.54 DPT, 1.33 2C-T-2, 0.73 5-MeO-DMT, 0.12 2C-B-fly; **0.00:** psilocin, 5-MeO-MIPT, 5-MeO-DIPT, DMT, 4C-T-2, DIPT, MDMA, DOI, mescaline, DOM, TMA, DOET, TMA-2, DOB, MDA, Aleph-2, MEM, 5-MeO-TMT, EMDT, ibogaine, salvinorin A; **ND:** morphine, THC

**D4:** 2.90 lisuride, 2.49 cis-2a, 2.39 LSD, 2.25 6-F-DMT, 2.20 DPT, 2.08 RR-2b, 1.62 SS-2c, 1.29 5-MeO-MIPT, 0.78 5-MeO-DMT; **0.00:** psilocin, MDMA, DMT, 2C-B, DIPT, 5-MeO-DIPT, mescaline, 4C-T-2, DOB, MDA, DOI, 2C-B-fly, DOM, TMA, DOET, TMA-2, 2C-E, 2C-T-2, Aleph-2, MEM, 5-MeO-TMT, EMDT, salvinorin A; **ND:** ibogaine, morphine, THC

**M3:** 3.07 MDMA, 2.51 DOI, 2.22 2C-B, 2.22 ibogaine, 1.99 2C-E, 1.94 2C-T-2, 1.53 DOB, 1.42 2C-B-fly, 0.92 Aleph-2; **0.00:** DMT, lisuride, 5-MeO-DMT, 5-MeO-MIPT, DIPT, psilocin, DPT, 4C-T-2, 6-F-DMT, mescaline, MEM, salvinorin A, 5-MeO-DIPT; **ND:** MDA, DOM, TMA, LSD, cis-2a, RR-2b, SS-2c, 5-MeO-TMT, EMDT, DOET, TMA-2, morphine, THC

**Alpha1A:** 3.17 6-F-DMT, 2.70 DMT, 2.56 DPT, 2.09 lisuride, 1.64 cis-2a, 1.56 DOM, 1.54 LSD, 0.67 SS-2c, 0.59 RR-2b; **0.00:** psilocin, MDMA, mescaline, 5-MeO-MIPT, DIPT, 5-MeO-DIPT, 5-MeO-DMT, 4C-T-2, DOB, MDA, DOI, 2C-B-fly, 2C-B, TMA, DOET, TMA-2, 2C-E, 2C-T-2, Aleph-2, MEM, salvinorin A; **ND:** 5-MeO-TMT, ibogaine, EMDT, morphine, THC

**KOR:** 4.00 salvinorin A, 2.88 ibogaine, 2.21 morphine; **0.00:** MDA, 2C-B, DMT, MDMA, mescaline, DOB, 2C-T-2, Aleph-2, MEM, 5-MeO-MIPT, DIPT, psilocin, 5-MeO-DMT, 2C-E, LSD, lisuride, DOI, 2C-B-fly, DOM, TMA, DOET, DPT, 4C-T-2, cis-2a, RR-2b, SS-2c, 5-MeO-TMT, EMDT, 5-MeO-DIPT; **ND:** TMA-2, 6-F-DMT, THC

**Beta1:** 2.89 DOI, 2.56 lisuride, 1.76 cis-2a, 1.72 SS-2c, 1.40 DOET, 1.39 LSD, 1.21 DOB, 0.81 2C-T-2, 0.37 Aleph-2; **0.00:** 5-MeO-DMT, DMT, mescaline, DOM, DIPT, 5-MeO-MIPT, DPT, 4C-T-2, 6-F-DMT, 5-MeO-DIPT, MDMA, 2C-B-fly, 2C-E, TMA, psilocin, TMA-2, ibogaine, MDA, MEM, salvinorin A; **ND:** 2C-B, 5-MeO-TMT, RR-2b, EMDT, morphine, THC

**MOR:** 4.00 morphine, 2.67 ibogaine; **0.00:** lisuride, mescaline, 2C-B, TMA, MDMA, DPT, DOB, 2C-T-2, Aleph-2, MEM, 5-MeO-MIPT, DIPT, psilocin, DOET, 2C-E, LSD, cis-2a, DOI, 2C-B-fly, DOM, EMDT, 5-MeO-DIPT, salvinorin A, 4C-T-2, SS-2c, RR-2b, 5-MeO-TMT; **ND:** MDA, DMT, 5-MeO-DMT, TMA-2, 6-F-DMT, THC

**M5:** 2.54 MDMA, 2.32 DOI, 2.16 2C-E, 1.60 2C-T-2, 1.01 2C-B-fly, 0.30 Aleph-2; **0.00:** 2C-B, DMT, DOB, MDA, DOET, 5-MeO-DMT, 5-MeO-MIPT, DIPT, 5-MeO-DIPT, DPT, 4C-T-2, 6-F-DMT, lisuride, MEM, mescaline, DOM, TMA, TMA-2, salvinorin A; **ND:** psilocin, LSD, RR-2b, cis-2a, 5-MeO-TMT, EMDT, ibogaine, SS-2c, morphine, THC

**Alpha1B:** 2.95 DMT, 2.86 DPT, 1.08 lisuride, 0.65 LSD; **0.00:** MDMA, 2C-B, psilocin, mescaline, DOB, 2C-T-2, Aleph-2, MEM, 5-MeO-MIPT, DIPT, 5-MeO-DIPT, 5-MeO-DMT, 4C-T-2, 6-F-DMT, MDA, DOI, 2C-B-fly, DOM, TMA, DOET, TMA-2, 2C-E, cis-2a, RR-2b, SS-2c, salvinorin A; **ND:** 5-MeO-TMT, ibogaine, EMDT, morphine, THC

**CB1:** 4.00 THC; **0.00:** MDA, MDMA, mescaline, 2C-B, DMT, psilocin, 5-MeO-DMT, 2C-E, 2C-T-2, Aleph-2, MEM, 2C-B-fly, DIPT, 5-MeO-DIPT, DPT, 4C-T-2, DOB, lisuride, DOI, TMA, DOM, 6-F-DMT, DOET; **ND:** 5-MeO-MIPT, LSD, TMA-2, RR-2b, SS-2c, 5-MeO-TMT, EMDT, ibogaine, salvinorin A, morphine, cis-2a

**M4:** 2.90 DOI, 2.43 MDMA, 1.24 2C-B-fly; **0.00:** DOB, 2C-B, lisuride, psilocin, DMT, 2C-E, 2C-T-2, Aleph-2, 5-MeO-DMT, 5-MeO-MIPT, DIPT, 5-MeO-DIPT, DPT, 4C-T-2, 6-F-DMT, mescaline, MEM, salvinorin A; **ND:** DOM, MDA, DOET, TMA, LSD, cis-2a, RR-2b, SS-2c, 5-MeO-TMT, EMDT, ibogaine, TMA-2, morphine, THC

**DAT:** 3.02 ibogaine, 2.33 DPT; **0.00:** DOB, MDA, 2C-B, DMT, MDMA, mescaline, 2C-E, 2C-T-2, Aleph-2, MEM, 5-MeO-MIPT, DIPT, psilocin, 5-MeO-DMT, 4C-T-2, 6-F-DMT, lisuride, DOI, 2C-B-fly, DOM, TMA, DOET, TMA-2, LSD, cis-2a, RR-2b, SS-2c, 5-MeO-TMT, EMDT, 5-MeO-DIPT, salvinorin A; **ND:** morphine, THC

**D5:** 2.05 LSD, 1.61 6-F-DMT, 1.59 lisuride, 1.53 RR-2b, 1.36 cis-2a, 1.00 SS-2c; **0.00:** psilocin, 5-MeO-DMT, 2C-B, DMT, MDMA, mescaline, 5-MeO-MIPT, DIPT, 5-MeO-DIPT, DPT, 4C-T-2, DOB, MDA, DOI, 2C-B-fly, DOM, TMA, DOET, TMA-2, 2C-E, 2C-T-2, Aleph-2, MEM, 5-MeO-TMT, EMDT, salvinorin A; **ND:** ibogaine, morphine, THC

**CB2:** 3.78 THC; **0.00:** MDA, DOI, mescaline, 2C-B, DMT, psilocin, 5-MeO-DMT, 2C-E, 2C-T-2, Aleph-2, MEM, 5-MeO-MIPT, DIPT, 5-MeO-DIPT, DPT, 4C-T-2, DOB, lisuride, DOET, 2C-B-fly, DOM, TMA, 6-F-DMT; **ND:** MDMA, LSD, TMA-2, RR-2b, SS-2c, 5-MeO-TMT, EMDT, ibogaine, salvinorin A, morphine, cis-2a

**Ca+Channel:** 3.26 MDMA; **0.00:** lisuride, DOB, mescaline, 5-MeO-MIPT, DMT, psilocin, 5-MeO-DMT, 2C-E, 2C-T-2, Aleph-2, MEM, 4C-T-2, DIPT, 5-MeO-DIPT, DPT, 6-F-DMT; **ND:** 2C-B, MDA, DOI, 2C-B-fly, DOM, TMA, DOET, TMA-2, LSD, cis-2a, RR-2b, SS-2c, 5-MeO-TMT, EMDT, ibogaine, salvinorin A, morphine, THC

**M1:** 2.23 DOI, 1.96 ibogaine, 1.15 2C-B-fly; **0.00:** lisuride, DOB, DMT, 2C-B, 5-MeO-DMT, 2C-E, 2C-T-2, Aleph-2, MEM, 5-MeO-MIPT, DIPT, psilocin, DPT, 4C-T-2, 6-F-DMT, mescaline, MDMA, salvinorin A, DOM, 5-MeO-DIPT; **ND:** MDA, TMA, LSD, cis-2a, RR-2b, SS-2c, 5-MeO-TMT, EMDT, DOET, TMA-2, morphine, THC

**NMDA:** 3.01 ibogaine; **0.00:** 2C-T-2, DOB, mescaline, 2C-B, TMA, MDMA, DPT, 2C-E, 6-F-DMT, Aleph-2, MEM, 5-MeO-MIPT, DIPT, DOET, 5-MeO-DIPT, 4C-T-2, DOM, DOI; **ND:** lisuride, 2C-B-fly, MDA, DMT, 5-MeO-DMT, TMA-2, LSD, cis-2a, RR-2b, SS-2c, 5-MeO-TMT, EMDT, psilocin, salvinorin A, morphine, THC

**M2:** 2.36 DOI, 1.72 ibogaine, 0.65 2C-B-fly; **0.00:** MDA, DOB, DMT, 2C-B, 5-MeO-DMT, 2C-E, 2C-T-2, Aleph-2, MEM, 5-MeO-MIPT, DIPT, psilocin, DPT, 4C-T-2, 6-F-DMT, lisuride, MDMA, mescaline, DOM, TMA, DOET, TMA-2, 5-MeO-DIPT, salvinorin A; **ND:** LSD, cis-2a, 5-MeO-TMT, EMDT, RR-2b, SS-2c, morphine, THC

**H2:** 2.44 6-F-DMT, 1.42 lisuride; **0.00:** MDMA, mescaline, 2C-B, DMT, psilocin, 5-MeO-DMT, 2C-E, 2C-T-2, Aleph-2, MEM, 5-MeO-MIPT, DIPT, 5-MeO-DIPT, DPT, 4C-T-2, DOB, MDA, DOI, 2C-B-fly, DOM, TMA, DOET, TMA-2, 5-MeO-TMT, EMDT; **ND:** RR-2b, SS-2c, LSD, cis-2a, ibogaine, salvinorin A, morphine, THC

**NET:** 1.97 5-MeO-TMT, 0.82 5-MeO-DMT; **0.00:** MDMA, MDA, DOB, DMT, psilocin, mescaline, 2C-E, 2C-T-2, Aleph-2, MEM, 2C-B, DIPT, 5-MeO-DIPT, DPT, 4C-T-2, 6-F-DMT, lisuride, DOI, 2C-B-fly, DOM, TMA, DOET, TMA-2, LSD, cis-2a, RR-2b, SS-2c, 5-MeO-MIPT, EMDT; **ND:** ibogaine, salvinorin A, morphine, THC

**DOR:** 0.72 morphine; **0.00:** 2C-T-2, DOI, mescaline, 2C-B, TMA, psilocin, DPT, DOB, LSD, Aleph-2, MEM, 5-MeO-MIPT, DIPT, 5-MeO-DIPT, DOET, 2C-E, 4C-T-2, cis-2a, RR-2b, 2C-B-fly, DOM, EMDT, ibogaine, salvinorin A, 5-MeO-TMT, SS-2c; **ND:** MDMA, lisuride, MDA, DMT, 5-MeO-DMT, TMA-2, 6-F-DMT, THC

### Activity Data

The NIMH-PDSP provided activity data for the twenty-five drugs of Fig. 1, for 5-HT_2A_ and 5-HT_2C_ (Table S3). While most compounds appear to be full agonists at the two receptors, there are a few exceptions. Lower values of activity were reported for psilocin, MDMA, DOM and the three control drugs: 4C-T-2, 6-fluoro-DMT, and lisuride.

## Discussion

Perhaps the most striking result of the NIMH-PDSP assays has been to show that the psychedelics interact with a large number of receptors (forty-two of the forty-nine sites at which most of the drugs were assayed). While the phenylalkylamines tend to be more selective than the tryptamines and ergolines, they generally can not be accurately characterized as selective for 5-HT_2_, as they are so widely described in the literature [1], [4], [6], [7], [14], [15]. Only DOB and MEM come close to fitting that description. Ironically, DOI has been one of the drugs of choice in studies of the molecular pharmacology of psychedelics, and has been widely assumed to be a 5-HT_2_-selective agent [4], [6], [7], [15], [16]. This study has revealed DOI to be one of the least selective of all psychedelics. Some of the literature on DOI may need to be reinterpreted. The same may be true of any studies whose conclusions rely on the assumption that psychedelics are selective. Studies requiring drugs selective for 5-HT_2_ should be conducted with DOB or MEM, and they should not be presented as typical or characteristic of psychedelics.

In addition to showing that psychedelics are not as selective as generally believed, the data presented also shows that they exhibit diverse patterns of receptor interactions. Different drugs emphasize different classes of receptors. 5-HT_2B_ is the best hit for thirteen drugs, and 5-HT_1A_ is the best hit for nine drugs. Five of the top six psychedelic receptors (Table 5) are 5-HT_1_ and 5-HT_2_ receptors. If we acknowledge the pervasiveness of the 5-HT_1_ and 5-HT_2_ receptors, and then look past them, we find that the set of thirty-five drugs emphasize a wide variety of receptors:

**5-HT_1A_ DOET**

**5-HT_1D_ 2C-B-fly**

**5-HT_2_ DOB, 2C-T-2**

**5-HT_2B_ MEM**

**5-HT_5A_ RR-2b**

**5-HT_6_ EMDT**, **5-MeO-TMT**, **6-F-DMT**

**5-HT_7_ DMT**, **5-MeO-MIPT**, **LSD**, **5-MeO-DMT**

**α_2A_ DOI**

**α_2C_ mescaline**, **lisuride**, **MDA**, **2C-E**

**β_2_ DOM**, **2C-B**, **Aleph-2**, **4C-T-2**

**H_1_ TMA-2**, **DPT**

**σ_2_ ibogaine**, **TMA**

**D_1_ psilocin**

**D_3_ cis-2a**, **SS-2c**

**I_1_ MDMA**, **DIPT**, **5-MeO-DIPT**

**κ salvinorin A**

**μ morphine**

**CB_1_ THC**

It should be possible to use this diverse set of drugs as probes into the roles played by the various receptor systems in the human mind. In the papers that follow, this possibility will be explored by synthesizing the NIMH-PDSP data together with the data on the human pharmacology of these drugs.

## Supporting Information

Figure S1 Receptor affinity profiles of psychedelic drugs, ordered by receptor type. The vertical axis is normalized pKi (npKi). Horizontal axis is a list of forty-two receptors, grouped by receptor type. The drugs are ordered to correspond roughly to similarity of structure and receptor affinity profiles. Colors correspond to classes of receptors. It can be seen at a glance that most, but not all of the drugs interact strongly with the serotonin receptors (beige), certain drugs interact strongly with the dopamine receptors (red), others with the adrenergic receptors (green), yet others with the histamine receptors (yellow), etc.
(0.14 MB DOC)

Figure S2 Receptor affinity profiles of psychedelic drugs, ordered by decreasing affinity. The vertical axis is normalized pKi (npKi). Horizontal axis is a list of forty-two receptors, arranged in order of decreasing affinity for each individual drug. The thirty-five drugs are arranged in order of decreasing breadth, based on the Bsq values of Table 3 and Fig. 4. Drugs at the top of the figure have the broadest receptor interactions (least selective), while drugs at the bottom of the figure have the narrowest receptor interactions (most selective). Colors correspond to classes of receptors, and are the same as used in Fig. S1. The black vertical bars represent a 100-fold drop in affinity relative to the receptor with the highest affinity. As a rule of thumb, this is presumed to be the limit of perceptible receptor interaction. Receptors to the right of the black bar should be imperceptible, while receptors to the left of the black bar should be perceptible, increasingly so the further left they are.
(0.18 MB DOC)

Figure S3 Receptor affinities at forty-two receptors across thirty-five drugs, ordered by decreasing breadth of receptor. The vertical axis is normalized pKi (npKi). Horizontal axis is a list of thirty-five drugs, ordered by decreasing affinity at the receptor. The forty-two receptors are arranged in order of decreasing breadth, based on the Bsq values of Table 5 and Fig. 5. Receptors at the top of the figure have the broadest interactions with the thirty-five drugs, while receptors at the bottom of the figure have the narrowest interactions with the thirty-five drugs. The black vertical bars represent a 100-fold drop in affinity relative to the receptor with the highest affinity at each drug. As a rule of thumb, this is presumed to be the limit of perceptible receptor interaction. Drugs to the right of the black bar should have imperceptible interactions with the receptor, while drugs to the left of the black bar should have perceptible interactions with the receptor, increasingly so the further left they are.
(0.20 MB DOC)

Table S1 Receptor affinity data for ibogaine. Table S1 reports receptor affinity data for ibogaine collected from the literature. The columns identify the receptor, the species from which the receptor was used, the tissue from which the receptor was used, the radioligand used in determining affinity, the Ki value in nanomoles or the IC50 value in nanomoles, and the literature reference from which the data was obtained.
(0.31 MB DOC)

Table S2 Raw affinity (Ki) data for thirty-five drugs at sixty-seven sites. The table has been divided into three sections. The first section displays forty-two sites at which most compounds were assayed and at least one “hit” (Ki<10,000 nm) was found (5ht1a, 5ht1b, 5ht1d, 5ht1e, 5ht2a, 5ht2b, 5ht2c, 5ht5a, 5ht6, 5ht7, D1, D2, D3, D4, D5, Alpha1A, Alpha1B, Alpha2A, Alpha2B, Alpha2C, Beta1, Beta2, SERT, DAT, NET, Imidazoline1, Sigma1, Sigma2, DOR, KOR, MOR, M1, M2, M3, M4, M5, H1, H2, CB1, CB2, Ca+Channel, NMDA/MK801). The second section displays seven sites at which most compounds were assayed, but at which there were no hits (5ht3, H3, H4, V1, V2, V3, GabaA). The third section displays the remaining eighteen sites, at which only a few compounds were assayed, and no hits were found (GabaB, mGluR1a, mGluR2, mGluR4, mGluR5, mGluR6, mGluR8, A2B2, A2B4, A3B2, A3B4, A4B2, A4B2**, A4B4, BZP (a1), EP3, MDR 1, PCP). Missing Ki values are indicated by “ND” meaning that no data is available, or by “PH” meaning that the primary assay “hit” (>50% inhibition), but the secondary assay was not performed. For the first section of the table, an extra row and column labeled “ND/PH” provides a count of missing Ki data in the two categories, for each compound and for each receptor.
(0.05 MB XLS)

Table S3 Activity data for twenty-five drugs at 5-HT2A and 5-HT2C. GF62 is the cell line that expresses the 5-HT2A receptor, and INI is the cell line that expresses the 5-HT2C receptor. The “EC50 nM” columns express the concentration that gives half of the maximal activity for that drug. The maximal activity is displayed in the “Emax±SEM” column, and represent Ca++ mobilization relative to 5-HT which should give an Emax value of 100%. Data for the drugs should produce lower Emax values. For a compound that gives, for example, 53% Emax, the EC50 is the concentration where 26.5% response occurs. Emax values above 100%±SEM are an artifact caused by extrapolation by the graphpad program when it doesn't have points at the top end to define the asymptote. The data represent the mean ± variance of computer-derived estimates from single experiments done in quadruplicate. Thus, the four observations are averaged and a single estimate with error is provided.
(0.13 MB DOC)

Table S4 Affinity (Ki) data transformed into pKi values for thirty-five drugs at forty-two sites. Table S4 presents the raw Ki data transformed into pKi values. Higher affinities produce lower Ki values, thus it is valuable to calculate: pKi = −log10(Ki). Higher affinities have higher pKi values, and each unit of pKi value corresponds to one order of magnitude of Ki value. ND means the data is not available, and UM means that Ki was measured as >10,000 nm. Generally, the highest Ki value generated by NIMH-PDSP is 10,000, which produces a pKi value of −4 (although a value of 10,450 was reported for 5-MeO-TMT). For non-PDSP data gathered from the literature, some values greater than 10,000 are reported (i.e. 12,500, 14,142, 22,486, 39,409 and 70,000 for ibogaine).
(0.04 MB XLS)

Table S5 Thirty-five drugs arranged in order of decreasing breadth at selected groups of receptors. The thirty-five drugs are arranged in order of decreasing breadth at groups of receptors, based on the breadth index Bsq. The drugs with the broadest receptor interactions within the group are found at the tops of the columns, and the drugs with the least receptor interactions are found at the bottoms of the columns. Some columns list the maximum npKi value for a group of receptors (e.g. 5-HT2max). In this example, the column lists the highest npKi value of the three 5-HT2 receptors (5-HT2A, 5-HT2B, and 5-HT2C). An entry of “ND” indicates that the statistic could not be calculated because some data is missing. For example, to calculate Bsq for 5-HT2, or npKi for 5-HT2max, we need npKi values for 5-HT2A, 5-HT2B, and 5-HT2C. If any one of the three values is missing, the statistics will be reported as ND. Values of 0.00 correspond to Ki values of >10,000. The same data is also presented in Table S6 Receptors in a group are listed in the column heading, or are represented with the following abbreviations: • 5-HT - 5-HT1A, 5-HT1B, 5-HT1D, 5-HT1E, 5-HT2A, 5-HT2B, 5-HT2C, 5-HT5A, 5-HT6, 5-HT7 • 5-HT2 - 5-HT2A, 5-HT2B, 5-HT2C • 5-HT2max - maximum of 5-HT2A, 5-HT2B, 5-HT2C • 5-HT2A/Cmax - maximum of 5-HT2A, 5-HT2C • 5-HT1 - 5-HT1A, 5-HT1B, 5-HT1D, 5-HT1E • 5-HT1max - maximum of 5-HT1A, 5-HT1B, 5-HT1D, 5-HT1E • Dmax - maximum of D1, D2, D3, D4, D5 • Adrenergic - α1A, α1B, α2A, α2B, α2C, β1, β2 • AdrenergicMax - maximum of α1A, α1B, α2A, α2B, α2C, β1, β2 • α1max - maximum of α1A, α1B • α2max - maximum of α2A, α2B, α2C • βmax - maximum of β1, β2 • Hmax - maximum of H1, H2 • σmax - maximum of σ1, σ 2 • Mmax - maximum of M1, M2, M3, M4, M5 • TransportersMax - maximum of SERT, DAT, NET • OpioidMax - maximum of DOR, KOR, MOR
(0.61 MB DOC)

Table S6 Table of npKi, Bsq, or npKimax values at individual or groups of receptors for thirty-five drugs. Table S6 presents the normalized pKi data (npKi) for thirty five drugs at forty-two receptors, transporters and ion channels. In addition, it presents the three breadth statistics (B, Bsq, Bexp) for each drug across all forty-two sites, the breadth statistic Bsq for sixteen groups of related sites, and the maximum npKi value for thirteen groups of related sites. A useful way to work with the table is to choose a column, and sort the data on that column (click “Data, “Sort,” select “Header row,” choose the column from the “Sort by” drop down and select “Descending,” finally clicking “OK”). Missing values are reported as “ND.” Values of 0.0000 correspond to Ki values of >10,000. Receptors in a group are listed in the column headings using the following abbreviations: • B - sum of npKi values across forty-two receptors • Bsq - square root of sum of squares of npKi values across forty-two receptors • Bexp - log of sum of exponents of npKi values across forty-two receptors • 5ht - 5-HT1A, 5-HT1B, 5-HT1D, 5-HT1E, 5-HT2A, 5-HT2B, 5-HT2C, 5-HT5A, 5-HT6, 5-HT7 • 5ht1 - 5-HT1A, 5-HT1B, 5-HT1D, 5-HT1E • 5ht1max - maximum of 5-HT1A, 5-HT1B, 5-HT1D, 5-HT1E • 5ht2 - 5-HT2A, 5-HT2B, 5-HT2C • 5ht2max - maximum of 5-HT2A, 5-HT2B, 5-HT2C • 5ht2[ac] - 5-HT2A, 5-HT2C • 5ht2[ac]max - maximum of 5-HT2A, 5-HT2C • 5ht[67] - 5-HT6, 5-HT7 • D[1–5] - D1, D2, D3, D4, D5 • D[1–5]max - maximum of D1, D2, D3, D4, D5 • ∧[AB] - α1A, α1B, α2A, α2B, α2C, β1, β2 • ∧[AB]max - maximum of α1A, α1B, α2A, α2B, α2C, β1, β2 • Alpha1 - α1A, α1B • Alpha1max - maximum of α1A, α1B • Alpha2 - α2A, α2B, α2C • Alpha2max - maximum of α2A, α2B, α2C • Beta - β1, β2 • BetaMax - maximum of β1, β2 • M[1–5] - M1, M2, M3, M4, M5 • M[1–5]max - maximum of M1, M2, M3, M4, M5 • Sigma - σ1, σ 2 • SigmaMax - maximum of σ1, σ 2 • H[12] - H1, H2 • H[1–2]max - maximum of H1, H2 • T$ - SERT, DAT, NET • T$max - maximum of SERT, DAT, NET • [DKM]OR - DOR, KOR, MOR • [DKM]ORmax - maximum of DOR, KOR, MOR • CB[12] - CB1, CB2
(0.05 MB XLS)

Table S7 Thirty-five drugs arranged in order of decreasing proportional interaction at selected groups of receptors. The thirty-five drugs are arranged in order of decreasing proportional interaction at groups of receptors, based on the proportional breadth index Bp. The drugs with the greatest proportional interactions are found at the tops of the columns, and the drugs with the least proportional interactions are found at the bottoms of the columns. An entry of “ND” indicates that the statistic could not be calculated because some data is missing. The same data is also presented in Table S8. Receptors in a group are listed in the column heading, or are represented with the following abbreviations: • 5-HT - 5-HT1A, 5-HT1B, 5-HT1D, 5-HT1E, 5-HT2A, 5-HT2B, 5-HT2C, 5-HT5A, 5-HT6, 5-HT7 • 5-HT1 - 5-HT1A, 5-HT1B, 5-HT1D, 5-HT1E • Adrenergic - α1A, α1B, α2A, α2B, α2C, β1, β2
(0.37 MB DOC)

Table S8 Table of Bp values at individual or groups of receptors for thirty-five drugs. Table S8 presents the proportional breadth statistic Bp for each drug at each of forty-two sites, and for sixteen groups of related sites. A useful way to work with the table is to choose a column, and sort the data on that column (click “Data, “Sort,” select “Header row,” choose the column from the “Sort by” drop down and select “Descending,” finally clicking “OK”). Missing values are reported as “ND.” Receptors in a group are listed in the column headings using the following abbreviations: • Bsq - square root of sum of squares of npKi values across forty-two receptors • 5ht - 5-HT1A, 5-HT1B, 5-HT1D, 5-HT1E, 5-HT2A, 5-HT2B, 5-HT2C, 5-HT5A, 5-HT6, 5-HT7 • 5ht1 - 5-HT1A, 5-HT1B, 5-HT1D, 5-HT1E • 5ht2 - 5-HT2A, 5-HT2B, 5-HT2C • 5ht2[ac] - 5-HT2A, 5-HT2C • 5ht[67] - 5-HT6, 5-HT7 • D[1–5] - D1, D2, D3, D4, D5 • ∧[AB] - α1A, α1B, α2A, α2B, α2C, β1, β2 • Alpha1 - α1A, α1B • Alpha2 - α2A, α2B, α2C • Beta - β1, β2 • M[1–5] - M1, M2, M3, M4, M5 • Sigma - σ1, σ 2 • H[12] - H1, H2 • T$ - SERT, DAT, NET • [DKM]OR - DOR, KOR, MOR • CB[12] - CB1, CB2
(0.04 MB XLS)
