# Correction: Psychedelics and the Human Receptorome

**DOI:** 10.1371/annotation/e580a864-cf13-40c2-9bd9-b9687a6f0fe4

**Published:** 2010-03-04

**Authors:** Thomas S. Ray

Figure 4 is a duplicate of Figure 3. Please view the correct Figure 4 here: 

**Figure:**
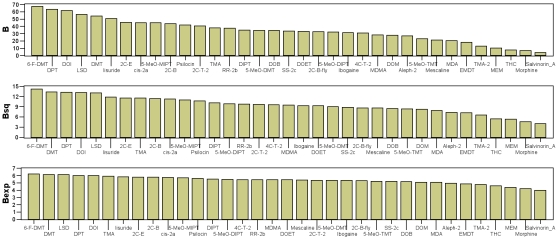


The titles and legends for both figures are correct as they appear. 

