# Supplementary material for: Psychedelics and the Human Receptorome
Source: PLoS One. 2010 Feb 2;5(2):e9019. doi: 10.1371/journal.pone.0009019 (PMC2814854; doi:10.1371/journal.pone.0009019)
Supplement: Table S1 — Receptor affinity data for ibogaine. Table S1 reports receptor affinity data for ibogaine collected from the literature. The columns identify the receptor, the species from which the receptor was used, the tissue from which the receptor was used, the radioligand used in determining affinity, the Ki value in nanomoles or the IC50 value in nanomoles, and the literature reference from which the data was obtained. (0.31 MB DOC) [file pone.0009019.s004.doc]

| Ibogaine | | | | | | |
| --- | --- | --- | --- | --- | --- | --- |
| Receptor | Species | Tissue | Radioligand | Ki(nM) | IC50(nM) | Ref |
| 5ht2a | rat | cloned | ketanserin | >10,000 |  | PDSP web; [1] |
|  |  |  | ketanserin | 12,500 |  | [2] |
|  |  | Gf-6 cells | ketanserin | 16,000 |  | [3] |
|  |  |  |  | **14,142** |  | **average** |
| 5ht2c | rat | cloned | mesulergine | >10,000 |  | PDSP web; [1] |
| 5ht1a | human | cloned | 8-oh-dpat | >10,000 |  | PDSP web; [1] |
| 5ht1b | rat | striatum | 5-HT | >100,000 |  | [4] |
| 5ht1d | bovine | caudate | 5-HT | >100,000 |  | [4] |
| D1 | human | cloned | SCH23390 | >10,000 |  | PDSP web; [1] |
| D2 | human | cloned | YM 09191-2 | >10,000 |  | PDSP web; [1] |
| D3 | calf | caudate | 7-OH-DPAT | 70000±1700 |  | [3] |
| D4 | human | cloned | spiperone |  | >100,000 | [5] |
| Beta1 | rat | cortex | dihydro-alprenalol HCL | >100,000 |  | [4] |
| SERT | human | occipital cortex | [125I]RTI-55 | 548.7±29.7 |  | [6] |
| DAT | human |  | [125I]RTI-121 | 1980±460 |  | [6] |
| Sigma1 | guinea pig | brain | (+)-pentazocine | 8554±1134 |  | [7] |
|  | guinea pig | brain | (+)-pentazocine | 9310±630 |  | Mach et al.1995 |
|  | calf | caudate | (+)-pentazocine | 2500±600 |  | [3] |
|  |  |  |  | **5839** |  | **average** |
| Sigma2 | rat | liver | DTG + dextrallorphan | 201±23 |  | [7] |
|  | rat | liver | DTG | 90.4±10.1 |  | [8] |
|  | guinea pig | brain | DTG | 250±39 |  | [8] |
|  | calf | hippocampus | DTG | 400±36 |  | [3] |
|  |  |  |  | **206** |  | **average** |
| M1 |  |  | pirenzepine | 31,600 |  | [2] |
|  | calf | cortex | pirenzepine | 16000±1000 |  | [3] |
|  | rat | forebrain | pirenzepine |  | 7600±700 | [5] |
|  |  |  |  | **22,486** |  | **average** |
| M2 |  |  | N-methylacoblamine | 50,100 |  | [2] |
|  | calf | cortex | QBN | 31000±3400 |  | [3] |
|  | rat | forebrain | AF-DX384 |  | 5900±1400 | [5] |
|  |  |  |  | **39,409** |  | **average** |
| M3 |  |  | N-methylacoblamine | 12,500 |  | [2] |
| H1 |  |  | pyrilamine |  | >10,000 | [5] |
| KOR | bovine | cortex | U69,593 | 2080±230 |  | [4] |
|  |  |  | U69,593 | 3162 |  | [2] |
|  | calf | cortex | U69,593 | 3770±810 |  | [9] |
|  | calf | cortex | U69,593 | 2200±100 |  | [3] |
|  | rat | forebrain | U69,593 |  | 16000±2100 | [5] |
|  | rat | forebrain | U69,593 |  | 29800±8300 | [10] |
|  | mouse | forebrain | U69,593 |  | 13800±600 | [10] |
|  | guinea pig | forebrain | U69,593 |  | 21000±1100 | [10] |
|  |  |  |  | **2717** |  | **average** |
| MOR | bovine | cortex | carfentanil | >100,000 |  | [4] |
|  | calf | cortex | DAGO | 11040±660 |  | [9] |
|  | calf | cortex | DAGO | 2000±150 |  | [3] |
|  | rat | forebrain | DAGO |  | 26000±7000 | [5] |
|  | rat | thalamic membraines |  | 3760±223 |  | Pablo et al. 1998 |
|  |  |  |  | **4362** |  | **average** |
| DOR | bovine | cortex | DPDPE | >100,000 |  | [4] |
| NMDA | rat | forebrain | MK-801 | 1010±100 |  | [11] |
|  | bovine | cortex | MK-801 | 3100±300 |  | [3] |
|  | rat | forebrain | CGS-19755 |  | >100,000 | [5] |
|  | rat | forebrain | MK-801 | 1110±30 |  | [10] |
|  | rat | hippocampus | MK-801 | 1190±90 |  | [12] |
|  | rat | cortex | MK-801 | 1810±10 |  | [12] |
|  | rat | striatum | MK-801 | 1970±80 |  | [12] |
|  | rat | Thalamus + hypothalamus | MK-801 | 1980±10 |  | [12] |
|  | rat | spinal cord | MK-801 | 2220±230 |  | [12] |
|  | rat | midbrain | MK-801 | 2460±200 |  | [12] |
|  | rat | cerebellum | MK-801 | 3310±160 |  | [12] |
|  | rat | basal forebrain | MK-801 | 3900±230 |  | [12] |
|  | rat | forebrain | dizocilpine |  | 3200±500 | [13] |
|  | rat |  | MK-801 |  | 5200±240 | [14] |
|  |  |  |  | **2001** |  | **average** |
| GABA-A | bovine | cortex | muscimol | >100,000 |  | [4] |
| 5HT3 | mouse | NG108-15 | GR65630 | >10,000 |  | PDSP web; [1] |
| GABA B | calf | cortex | GABA | >100,000 |  | [3] |
| PCP | rat | forebrain | TCP |  | 50500±100000 | [5] |

Reference List

1. Toll L, Berzetei-Gurske IP, Polgar WE, Brandt SR, Adapa ID et al. (1998) Standard binding and functional assays related to medications development division testing for potential cocaine and opiate narcotic treatment medications. NIDA Research Monograph 178: 440-66.

2. Repke DB, Artis DR, Nelson JT, Wong EHF (1994) Abbreviated Ibogaine Congeners. Synthesis and Reactions of Tropan-3-yl-2- and -3-indoles. Investigation of an Unusual Isomerization of 2-Substituted Indoles Using Computational and Spectroscopic Techniques. Journal of Organic Chemistry 59: 2164.

3. Glick SD, Maisonneuve IM, Szumlinski KK (2000) 18-Methoxycoronaridine (18-MC) and ibogaine: comparison of antiaddictive efficacy, toxicity, and mechanisms of action. Annals of the New York Academy of Sciences 914: 369-86.

4. Deecher DC, Teitler M, Soderlund DM, Bornmann WG, Kuehne ME et al. (1992) Mechanisms of action of ibogaine and harmaline congeners based on radioligand binding studies. Brain Research 571: 242-7.

5. Sweetnam PM, Lancaster J, Snowman A, Collins JL, Perschke S et al. (1995) Receptor binding profile suggests multiple mechanisms of action are responsible for ibogaine's putative anti-addictive activity. Psychopharmacology (Berl) 118: 369-76.

6. Mash DC, Staley JK, Baumann MH, Rothman RB, Hearn WL (1995) Identification of a primary metabolite of ibogaine that targets serotonin transporters and elevates serotonin. Life Sciences 57: L45-L50.

7. Bowen WD, Vilner BJ, Williams W, Bertha CM, Kuehne ME et al. (1995) Ibogaine and its congeners are sigma 2 receptor-selective ligands with moderate affinity. European Journal of Pharmacology 279: R1-R3.

8. Mach RH, Smith CR, Childers SR (1995) Ibogaine possesses a selective affinity for sigma 2 receptors. Life Sci 57: L57-L62.

9. Pearl SM, Herrick-Davis K, Teitler M, Glick SD (1995) Radioligand-binding study of noribogaine, a likely metabolite of ibogaine. Brain Research 675: 342-4.

10. Layer RT, Skolnick P, Bertha CM, Bandarage UK, Kuehne ME et al. (1996) Structurally modified ibogaine analogs exhibit differing affinities for NMDA receptors. European Journal of Pharmacology 309: 159-65.

11. Popik P, Layer RT, Skolnick P (1994) The putative anti-addictive drug ibogaine is a competitive inhibitor of [3H]MK-801 binding to the NMDA receptor complex. Psychopharmacology (Berl) 114: 672-4.

12. Popik P, Layer RT, Fossom LH, Benveniste M, Geter-Douglass B et al. (1995) NMDA antagonist properties of the putative antiaddictive drug, ibogaine. The Journal of Pharmacology and Experimental Therapeutics 275: 753-60.

13. Chen K, Kokate TG, Donevan SD, Carroll FI, Rogawski MA (1996) Ibogaine block of the NMDA receptor: in vitro and in vivo studies. Neuropharmacology 35: 423-31.

14. Staley JK, Ouyang Q, Pablo J, Hearn WL, Flynn DD et al. (1996) Pharmacological screen for activities of 12-hydroxyibogamine: a primary metabolite of the indole alkaloid ibogaine. Psychopharmacology (Berl) 127: 10-8.
