# Supplementary material for: Psychedelics and the Human Receptorome
Source: PLoS One. 2010 Feb 2;5(2):e9019. doi: 10.1371/journal.pone.0009019 (PMC2814854; doi:10.1371/journal.pone.0009019)
Supplement: Table S3 — Activity data for twenty-five drugs at 5-HT2A and 5-HT2C. GF62 is the cell line that expresses the 5-HT2A receptor, and INI is the cell line that expresses the 5-HT2C receptor. The “EC50 nM” columns express the concentration that gives half of the maximal activity for that drug. The maximal activity is displayed in the “Emax±SEM” column, and represent Ca++ mobilization relative to 5-HT which should give an Emax value of 100%. Data for the drugs should produce lower Emax values. For a compound that gives, for example, 53% Emax, the EC50 is the concentration where 26.5% response occurs. Emax values above 100%±SEM are an artifact caused by extrapolation by the graphpad program when it doesn't have points at the top end to define the asymptote. The data represent the mean ± variance of computer-derived estimates from single experiments done in quadruplicate. Thus, the four observations are averaged and a single estimate with error is provided. (0.13 MB DOC) [file pone.0009019.s006.doc]

|  | GF62 - 5HT2A | | INI - 5HT2C | |
| --- | --- | --- | --- | --- |
|  | EC_50_ nM | E_max_±SEM | EC_50_ nM | E_max_±SEM |
|  |  | (%) |  | (%) |
| (±)-MDA | 1443 | 84 ± 3.4 | 328 | 79 ± 14 |
| (±)-MDA | 1767 | 99.09 ± 1.722 | 98.24 | 118.1 ± 13.81 |
| S-(+)-MDA | >10,000 | NS | 3853 | 104.4 ± 7.354 |
| R-(-)-MDA | 333.5 | 85.31 ± 2.09 | 18.51 | 91.2 ± 5.6 |
| Mescaline | 118 | 102 ± 5 | 30 | 22 ± 4.8 |
| Mescaline | 208.4 | 107 ± 2.6 | 114.1 | 60.74 ± 15.95 |
| Mescaline | 87.6 | 102.9 ± 3.6 | 20 | 94.9 ± 8.1 |
| DIPT | 344.2 | 89 ± 1.575 | 311.4 | 143 ± 24.46 |
| DIPT |  |  | 166.5 | 80.99 ± 1.644 |
| DIPT | 1411 | 117 ± 2.6 | 1999 | 107.3 ± 2.6 |
| 5-MeO-DIPT | 215.2 | 113.4 ± 3.8 | 392.5 | 174.1 ± 27 |
| 5-MeO-DIPT |  |  | 73.55 | 100.3 ± 1.528 |
| (±)-TMA | 41.25 | 96.21 ± 4.6 | 47.37 | 92.62 ± 6.97 |
| 2C-B | 1.888 | 95.48 ± 8.5 | 0.0311 | 116 ± 9.124 |
| 2C-B | 1.927 | 99.45 ± 4.7 | 0.2636 | 104.1 ± 8.659 |
| 2C-E | 2.894 | 86.9 ± 5 | 0.642 | 106.3 ± 4.5 |
| 2C-E | 3.162 | 85.35 ± 4.35 | 0.2331 | 99.83 ± 5.108 |
| 2C-E | 2.5 | 77.73 ± 2.8 | 1.076 | 102.1 ± 2.793 |
| 2C-T-2 | 0.354 | 107.2 ± 8.6 | 0.0233 | 88.29 ± 11.13 |
| 2C-T-2 | 0.9455 | 128.4 ± 7.3 | 0.0449 | 107.1 ± 11.57 |
| 2C-T-2 | 1.215 | 97.95 ± 4.1 | 0.2 | 86.98 ± 3.19 |
| 2C-B-fly | 1.103 | 104 ± 6 | 0.1488 | 108.4 ± 3.97 |
| 2C-B-fly | 0.2553 | 99.05 ± 6.6 | 0.0615 | 108.7 ± 7.761 |
| 2C-B-fly | 0.029 | 97.84 ± 3.9 | 0.089 | 99.92 ± 4.675 |
| (±)-DOB | 0.52 | 89 ± 3.6 | 0.314 | 95 ± 3.9 |
| (±)-DOB | 0.9267 | 96.88 ± 4.28 | 0.2465 | 111.7 ± 8.324 |
| S-(+)-DOB | 0.66 | 93.53 ± 3.69 | 0.063 | 105.7 ± 5.95 |
| R-(-)-DOB | 0.018 | 129.7 ± 7.44 | 0.0158 | 89.95 ± 6.4 |
| (±)-Aleph-2 | 0.4886 | 104 ± 6.3 | 0.0912 | 114.2 ± 3.687 |
| (±)-Aleph-2 |  |  | 0.1411 | 104.5 ± 6.692 |
| (±)-Aleph-2 | 0.8975 | 108.2 ± 7.0 | 0.401 | 108 ± 3.319 |
| (±)-DOM | 7.527 | 132 ± 3 | 2.673 | 113.2 ± 4.3 |
| (±)-DOM | 9.125 | 103 ± 6.8 | 0.5164 | 107.2 ± 7.5 |
| (±)-DOM | 4.391 | 114.8 ± 5.64 | 0.2345 | 100.8 ± 3.131 |
| (±)-DOM | 2.649 | 103.8 ± 5.73 | 1.614 | 119 ± 5.5 |
| R-(-)-DOM | 14.82 | 96.96 ± 4.107 | 15.61 | 75.9 ± 3.616 |
| S-(+)-DOM | 370.9 | 71.88 ± 1.628 | 160.1 | 75.47 ± 2.725 |
| (±)-DOET | 2.395 | 112.2 ± 6.5 | 9.178 | 81.7 ± 5.45 |
| S-(+)-DOET | 13.55 | 91.52 ± 2.517 | 10.35 | 79.43 ± 5.512 |
| R-(-)-DOET | 0.9645 | 117 ± 4.72 | 4.611 | 104.5 ± 5.482 |
| (±)-DOI | 1.006 | 111 ± 2.5 | 0.4304 | 109.7 ± 4.9 |
| (±)-DOI | 0.4182 | 105 ± 6.5 | 0.14 | 105.6 ± 7.771 |
| S-(+)-DOI | 2.847 | 85.93 ± 3.12 | 1.352 | 95.3 ± 3.491 |
| R-(-)-DOI | 0.1809 | 101.3 ± 4.012 | 0.098 | 95.94 ± 2.55 |
| Psilocin | 21 | 94 ± 3.5 | 19 | 96.5 ± 3.75 |
| Psilocin | 25.51 | 72 ± 1.598 | 1.952 | 124 ± 8.3 |
| Psilocin |  |  | 5.62 | 101.3 ± 3.6 |
| Psilocin | 161.5 | 50.55 ± 2.029 | 21.77 | 93.29 ± 6.180 |
| 5-MeO-MIPT | 20.22 | 101.5 ± 1.84 | 7.517 | 123 ± 3.6 |
| 5-MeO-MIPT |  |  | 2.784 | 79.45 ± 5.479 |
| 5-MeO-MIPT | 285.4 | 94.4 ± 2.2 | 221.3 | 95.35 ± 3.6 |
| 5-MeO-DMT | 49.57 | 94.6 ± 2.9 | 4.227 | 102.6 ± 8.7 |
| 5-MeO-DMT | 56.93 | 90 ± 1.76 | 2.646 | 98.51 ± 3.663 |
| 5-MeO-DMT | 183 | 97 ± 3.5 | 23.55 | 100.4 ± 5.97 |
| DPT | 56.35 | 97 ± 4.6 | 21.75 | 118 ± 16 |
| DPT |  |  | 85.93 | 105.1 ± 3.406 |
| DPT | 372 | 91.9 ± 4.3 | 381 | 94.38 ± 2.6 |
| 6-fluoro-DMT | 41.3 | 74.15 ± 2.4 | 1.252 | 131 ± 4.9 |
| 6-fluoro-DMT |  |  | 5.816 | 105 ± 2.148 |
| DMT | 181 | 84 ± 3.2 | 80 | 86 ± 6.5 |
| DMT | 439.9 | 95.08 ± 3.945 | 28.91 | 120.3 ± 12.56 |
| lisuride | 343 | 73.13 ± 2.8 | 23614 | 1475 ± 91301 |
| lisuride |  |  | 203.1 | 78.66 ± 4.952 |
| (±)-MEM | 47.5 | 98.8 ± 1.95 | 32.78 | 129.1 ± 7.337 |
| (±)-MEM |  |  | 29.85 | 98.03 ± 1.945 |
| (±)-MEM | 176.8 | 87.59 ± 4.463 | 247.6 | 98.59 ± 5.25 |
| (±)-4C-T-2 | 13.06 | 77.5 ± 2.222 | 7.333 | 121 ± 6.7 |
| (±)-4C-T-2 |  |  | 13.22 | 85.55 ± 3.884 |
| (±)-MDMA | 12484 | 484.3 ± 629 | 831 | 142 ± 7.7 |
| (±)-MDMA | 8284 | 70.41 ± 14.3 | 4299 | 249.3 ± 3639 |
| S-(+)-MDMA | NS |  | 121.4 | 15.86 ± 2.632 |
| R-(-)-MDMA | 126.4 | 102.5 ± 4.242 | 9.858 | 111.2 ± 1.934 |
