# Supplementary material for: Psychedelics and the Human Receptorome
Source: PLoS One. 2010 Feb 2;5(2):e9019. doi: 10.1371/journal.pone.0009019 (PMC2814854; doi:10.1371/journal.pone.0009019)
Supplement: Table S5 — Thirty-five drugs arranged in order of decreasing breadth at selected groups of receptors. The thirty-five drugs are arranged in order of decreasing breadth at groups of receptors, based on the breadth index Bsq. The drugs with the broadest receptor interactions within the group are found at the tops of the columns, and the drugs with the least receptor interactions are found at the bottoms of the columns. Some columns list the maximum npKi value for a group of receptors (e.g. 5-HT2max). In this example, the column lists the highest npKi value of the three 5-HT2 receptors (5-HT2A, 5-HT2B, and 5-HT2C). An entry of “ND” indicates that the statistic could not be calculated because some data is missing. For example, to calculate Bsq for 5-HT2, or npKi for 5-HT2max, we need npKi values for 5-HT2A, 5-HT2B, and 5-HT2C. If any one of the three values is missing, the statistics will be reported as ND. Values of 0.00 correspond to Ki values of >10,000. The same data is also presented in Table S6 Receptors in a group are listed in the column heading, or are represented with the following abbreviations: • 5-HT - 5-HT1A, 5-HT1B, 5-HT1D, 5-HT1E, 5-HT2A, 5-HT2B, 5-HT2C, 5-HT5A, 5-HT6, 5-HT7 • 5-HT2 - 5-HT2A, 5-HT2B, 5-HT2C • 5-HT2max - maximum of 5-HT2A, 5-HT2B, 5-HT2C • 5-HT2A/Cmax - maximum of 5-HT2A, 5-HT2C • 5-HT1 - 5-HT1A, 5-HT1B, 5-HT1D, 5-HT1E • 5-HT1max - maximum of 5-HT1A, 5-HT1B, 5-HT1D, 5-HT1E • Dmax - maximum of D1, D2, D3, D4, D5 • Adrenergic - α1A, α1B, α2A, α2B, α2C, β1, β2 • AdrenergicMax - maximum of α1A, α1B, α2A, α2B, α2C, β1, β2 • α1max - maximum of α1A, α1B • α2max - maximum of α2A, α2B, α2C • βmax - maximum of β1, β2 • Hmax - maximum of H1, H2 • σmax - maximum of σ1, σ 2 • Mmax - maximum of M1, M2, M3, M4, M5 • TransportersMax - maximum of SERT, DAT, NET • OpioidMax - maximum of DOR, KOR, MOR (0.61 MB DOC) [file pone.0009019.s008.doc]

| 5-HT | |  | 5-HT_2_ | |  | 5-HT_2_max | |  | 5-HT_2A_, 5-HT_2C_ | |  | 5-HT_2A/C_max | |
| --- | --- | --- | --- | --- | --- | --- | --- | --- | --- | --- | --- | --- | --- |
| B_sq_ | Drug |  | B_sq_ | Drug |  | npK_i_ | Drug |  | B_sq_ | Drug |  | npK_i_ | Drug |
| 11.13 | LSD |  | 6.45 | 2C-E |  | 4.00 | 2C-E |  | 5.28 | DOI |  | 4.00 | DOI |
| 10.14 | 6-F-DMT |  | 6.30 | 2C-B |  | 4.00 | 2C-B |  | 5.06 | 2C-E |  | 3.76 | 2C-E |
| 9.83 | DMT |  | 6.14 | DOI |  | 4.00 | DOI |  | 4.87 | 2C-B |  | 3.72 | DOET |
| 9.74 | 2C-B |  | 6.11 | DOET |  | 4.00 | 4C-T-2 |  | 4.86 | DOET |  | 3.69 | 2C-B |
| 9.47 | 2C-E |  | 6.05 | 4C-T-2 |  | 4.00 | 2C-T-2 |  | 4.71 | LSD |  | 3.54 | LSD |
| 9.32 | 5-MeO-MIPT |  | 5.95 | 2C-T-2 |  | 4.00 | DOB |  | 4.54 | 4C-T-2 |  | 3.42 | TMA-2 |
| 9.20 | Psilocin |  | 5.94 | DOB |  | 4.00 | TMA-2 |  | 4.40 | 2C-T-2 |  | 3.42 | DMT |
| 9.06 | TMA |  | 5.86 | TMA-2 |  | 4.00 | 2C-B-fly |  | 4.39 | DOB |  | 3.33 | 4C-T-2 |
| 8.68 | DPT |  | 5.80 | DMT |  | 4.00 | Aleph-2 |  | 4.29 | TMA-2 |  | 3.23 | DOB |
| 8.68 | RR-2b |  | 5.74 | 2C-B-fly |  | 4.00 | Psilocin |  | 4.28 | DMT |  | 3.18 | 2C-T-2 |
| 8.26 | DOI |  | 5.64 | LSD |  | 4.00 | TMA |  | 4.11 | 2C-B-fly |  | 3.02 | TMA |
| 8.07 | 5-MeO-DMT |  | 5.31 | 6-F-DMT |  | 4.00 | DOM |  | 3.72 | cis-2a |  | 2.93 | 2C-B-fly |
| 8.06 | DOET |  | 5.30 | Aleph-2 |  | 4.00 | MEM |  | 3.57 | 6-F-DMT |  | 2.89 | cis-2a |
| 7.99 | lisuride |  | 5.19 | Psilocin |  | 4.00 | MDA |  | 3.48 | Aleph-2 |  | 2.74 | lisuride |
| 7.89 | 2C-T-2 |  | 5.01 | TMA |  | 3.97 | Mescaline |  | 3.31 | Psilocin |  | 2.58 | 6-F-DMT |
| 7.85 | 2C-B-fly |  | 4.97 | DPT |  | 3.93 | 6-F-DMT |  | 3.11 | DPT |  | 2.52 | Psilocin |
| 7.43 | 4C-T-2 |  | 4.87 | DOM |  | 3.91 | DMT |  | 3.08 | SS-2c |  | 2.50 | Aleph-2 |
| 7.39 | 5-MeO-DIPT |  | 4.60 | cis-2a |  | 3.91 | 5-MeO-DIPT |  | 3.02 | TMA |  | 2.44 | 5-MeO-MIPT |
| 7.04 | DOB |  | 4.57 | MEM |  | 3.88 | DPT |  | 3.00 | 5-MeO-MIPT |  | 2.36 | DOM |
| 6.39 | DIPT |  | 4.54 | MDA |  | 3.72 | DOET |  | 2.78 | DOM |  | 2.32 | SS-2c |
| 6.38 | DOM |  | 4.48 | 5-MeO-MIPT |  | 3.64 | MDMA |  | 2.74 | lisuride |  | 2.31 | DPT |
| 6.23 | Mescaline |  | 4.08 | lisuride |  | 3.54 | LSD |  | 2.21 | MEM |  | 2.21 | MEM |
| 5.86 | TMA-2 |  | 3.97 | Mescaline |  | 3.48 | DIPT |  | 2.19 | RR-2b |  | 2.17 | 5-MeO-TMT |
| 5.76 | Aleph-2 |  | 3.91 | 5-MeO-DIPT |  | 3.32 | 5-MeO-MIPT |  | 2.17 | 5-MeO-TMT |  | 2.16 | Ibogaine |
| 5.67 | MDA |  | 3.77 | SS-2c |  | 3.01 | lisuride |  | 2.16 | Ibogaine |  | 2.15 | MDA |
| 4.97 | MEM |  | 3.64 | MDMA |  | 2.89 | cis-2a |  | 2.15 | MDA |  | 1.95 | EMDT |
| 3.64 | MDMA |  | 3.48 | DIPT |  | 2.32 | SS-2c |  | 1.95 | EMDT |  | 1.63 | RR-2b |
| 0.00 | Salvinorin A |  | 2.84 | RR-2b |  | 1.81 | RR-2b |  | 1.83 | 5-MeO-DMT |  | 1.55 | 5-MeO-DMT |
| ND | cis-2a |  | 1.96 | 5-MeO-DMT |  | 1.55 | 5-MeO-DMT |  | 0.00 | Mescaline |  | 0.00 | Mescaline |
| ND | Ibogaine |  | 0.00 | Salvinorin A |  | 0.00 | Salvinorin A |  | 0.00 | 5-MeO-DIPT |  | 0.00 | 5-MeO-DIPT |
| ND | 5-MeO-TMT |  | ND | Ibogaine |  | ND | Ibogaine |  | 0.00 | MDMA |  | 0.00 | MDMA |
| ND | SS-2c |  | ND | 5-MeO-TMT |  | ND | 5-MeO-TMT |  | 0.00 | DIPT |  | 0.00 | DIPT |
| ND | EMDT |  | ND | EMDT |  | ND | EMDT |  | 0.00 | Salvinorin A |  | 0.00 | Salvinorin A |
| ND | THC |  | ND | THC |  | ND | THC |  | ND | THC |  | ND | THC |
| ND | Morphine |  | ND | Morphine |  | ND | Morphine |  | ND | Morphine |  | ND | Morphine |

| 5-HT_1_ | |  | 5-HT_1_max | |  | 5-HT_6_, 5-HT_7_ | |  | D_1_, D_2_, D_3_, D_4_, D_5_ | |  | Dmax | |
| --- | --- | --- | --- | --- | --- | --- | --- | --- | --- | --- | --- | --- | --- |
| B_sq_ | Drug |  | npK_i_ | Drug |  | B_sq_ | Drug |  | B_sq_ | Drug |  | npK_i_ | Drug |
| 7.10 | LSD |  | 4.00 | LSD |  | 5.52 | 6-F-DMT |  | 5.65 | LSD |  | 3.51 | DMT |
| 6.54 | RR-2b |  | 4.00 | RR-2b |  | 5.39 | 5-MeO-TMT |  | 5.58 | cis-2a |  | 3.46 | cis-2a |
| 6.52 | TMA |  | 4.00 | 5-MeO-MIPT |  | 5.32 | LSD |  | 5.27 | 6-F-DMT |  | 3.37 | Psilocin |
| 6.35 | 2C-B |  | 4.00 | 5-MeO-DMT |  | 5.16 | DMT |  | 4.45 | lisuride |  | 3.16 | LSD |
| 6.26 | 5-MeO-MIPT |  | 4.00 | DPT |  | 4.84 | EMDT |  | 4.37 | RR-2b |  | 2.90 | lisuride |
| 6.19 | 6-F-DMT |  | 4.00 | 5-MeO-DIPT |  | 4.82 | 5-MeO-MIPT |  | 4.30 | Psilocin |  | 2.82 | SS-2c |
| 6.07 | 5-MeO-DMT |  | 4.00 | lisuride |  | 4.63 | cis-2a |  | 3.99 | SS-2c |  | 2.67 | 6-F-DMT |
| 6.06 | 2C-E |  | 4.00 | DOET |  | 4.59 | 5-MeO-DMT |  | 3.58 | DPT |  | 2.66 | 4C-T-2 |
| 5.87 | DPT |  | 4.00 | DIPT |  | 3.98 | Psilocin |  | 3.51 | DMT |  | 2.56 | DIPT |
| 5.81 | Psilocin |  | 4.00 | cis-2a |  | 3.84 | 2C-B |  | 3.05 | 5-MeO-DMT |  | 2.40 | RR-2b |
| 5.49 | 5-MeO-DIPT |  | 4.00 | SS-2c |  | 3.80 | TMA |  | 2.95 | 2C-E |  | 2.38 | 5-MeO-DMT |
| 5.15 | DMT |  | 3.97 | DMT |  | 3.77 | RR-2b |  | 2.66 | 4C-T-2 |  | 2.37 | DPT |
| 4.95 | lisuride |  | 3.81 | 2C-B-fly |  | 3.72 | lisuride |  | 2.56 | DIPT |  | 2.27 | 2C-E |
| 4.92 | 2C-B-fly |  | 3.71 | 2C-B |  | 3.56 | DPT |  | 2.13 | 5-MeO-MIPT |  | 1.70 | 5-MeO-MIPT |
| 4.83 | DOET |  | 3.66 | 6-F-DMT |  | 3.46 | SS-2c |  | 2.05 | 2C-B |  | 1.68 | DOB |
| 4.79 | Mescaline |  | 3.61 | Mescaline |  | 3.37 | 2C-E |  | 2.02 | 2C-T-2 |  | 1.67 | DOI |
| 4.72 | DIPT |  | 3.54 | 2C-E |  | 3.03 | 5-MeO-DIPT |  | 1.68 | DOB |  | 1.60 | 2C-B |
| 4.63 | DOI |  | 3.45 | TMA |  | 3.01 | DOI |  | 1.67 | DOI |  | 1.51 | 2C-T-2 |
| 4.58 | 2C-T-2 |  | 3.40 | Psilocin |  | 2.55 | DIPT |  | 1.41 | Aleph-2 |  | 1.41 | Aleph-2 |
| 3.48 | DOM |  | 3.00 | DOI |  | 2.43 | 2C-T-2 |  | 0.00 | 5-MeO-TMT |  | 0.00 | 5-MeO-TMT |
| 3.05 | DOB |  | 2.84 | 2C-T-2 |  | 2.41 | MDA |  | 0.00 | EMDT |  | 0.00 | EMDT |
| 2.70 | 4C-T-2 |  | 2.75 | DOM |  | 2.20 | DOM |  | 0.00 | TMA |  | 0.00 | TMA |
| 2.38 | MDA |  | 2.38 | MDA |  | 2.18 | 4C-T-2 |  | 0.00 | 5-MeO-DIPT |  | 0.00 | 5-MeO-DIPT |
| 1.82 | Aleph-2 |  | 2.04 | 4C-T-2 |  | 2.13 | 2C-B-fly |  | 0.00 | MDA |  | 0.00 | MDA |
| 0.00 | TMA-2 |  | 1.79 | DOB |  | 2.07 | DOET |  | 0.00 | DOM |  | 0.00 | DOM |
| 0.00 | MEM |  | 1.02 | Aleph-2 |  | 2.07 | DOB |  | 0.00 | DOET |  | 0.00 | DOET |
| 0.00 | MDMA |  | 0.00 | TMA-2 |  | 1.95 | MEM |  | 0.00 | MEM |  | 0.00 | MEM |
| 0.00 | Salvinorin A |  | 0.00 | MEM |  | 1.30 | Aleph-2 |  | 0.00 | Mescaline |  | 0.00 | Mescaline |
| ND | cis-2a |  | 0.00 | MDMA |  | 0.00 | Mescaline |  | 0.00 | TMA-2 |  | 0.00 | TMA-2 |
| ND | SS-2c |  | 0.00 | Salvinorin A |  | 0.00 | TMA-2 |  | 0.00 | MDMA |  | 0.00 | MDMA |
| ND | 5-MeO-TMT |  | ND | 5-MeO-TMT |  | 0.00 | MDMA |  | 0.00 | Salvinorin A |  | 0.00 | Salvinorin A |
| ND | Ibogaine |  | ND | Ibogaine |  | 0.00 | Salvinorin_A |  | ND | 2C-B-fly |  | ND | 2C-B-fly |
| ND | EMDT |  | ND | EMDT |  | ND | THC |  | ND | Ibogaine |  | ND | Ibogaine |
| ND | THC |  | ND | THC |  | ND | Morphine |  | ND | THC |  | ND | THC |
| ND | Morphine |  | ND | Morphine |  | ND | Ibogaine |  | ND | Morphine |  | ND | Morphine |

| Adrenergic | |  | AdrenergicMax | |  | α_1A_, α_1B_ | |  | α_1_max | |
| --- | --- | --- | --- | --- | --- | --- | --- | --- | --- | --- |
| B_sq_ | Drug |  | npK_i_ | Drug |  | B_sq_ | Drug |  | npK_i_ | Drug |
| 7.45 | lisuride |  | 4.00 | Mescaline |  | 4.00 | DMT |  | 3.17 | 6-F-DMT |
| 7.30 | DOI |  | 3.88 | lisuride |  | 3.83 | DPT |  | 2.95 | DMT |
| 6.96 | DMT |  | 3.79 | DOI |  | 3.17 | 6-F-DMT |  | 2.86 | DPT |
| 6.28 | DPT |  | 3.67 | 4C-T-2 |  | 2.35 | lisuride |  | 2.09 | lisuride |
| 5.50 | MDA |  | 3.60 | MDA |  | 1.67 | LSD |  | 1.64 | cis-2a |
| 5.43 | 6-F-DMT |  | 3.53 | DMT |  | 1.64 | cis-2a |  | 1.56 | DOM |
| 5.34 | MDMA |  | 3.38 | DOM |  | 1.56 | DOM |  | 1.54 | LSD |
| 5.30 | DOM |  | 3.36 | TMA |  | 0.67 | SS-2c |  | 0.67 | SS-2c |
| 4.95 | Mescaline |  | 3.24 | 6-F-DMT |  | 0.59 | RR-2b |  | 0.59 | RR-2b |
| 4.49 | TMA |  | 3.21 | MDMA |  | 0.00 | Mescaline |  | 0.00 | Mescaline |
| 4.38 | DOET |  | 2.97 | DPT |  | 0.00 | DOI |  | 0.00 | DOI |
| 4.12 | 2C-T-2 |  | 2.85 | 5-MeO-MIPT |  | 0.00 | 4C-T-2 |  | 0.00 | 4C-T-2 |
| 4.10 | 5-MeO-MIPT |  | 2.79 | Aleph-2 |  | 0.00 | MDA |  | 0.00 | MDA |
| 3.75 | DIPT |  | 2.68 | DIPT |  | 0.00 | TMA |  | 0.00 | TMA |
| 3.67 | 4C-T-2 |  | 2.66 | 5-MeO-DIPT |  | 0.00 | MDMA |  | 0.00 | MDMA |
| 3.59 | 5-MeO-DIPT |  | 2.56 | 2C-T-2 |  | 0.00 | 5-MeO-MIPT |  | 0.00 | 5-MeO-MIPT |
| 3.48 | DOB |  | 2.40 | DOET |  | 0.00 | Aleph-2 |  | 0.00 | Aleph-2 |
| 3.13 | Aleph-2 |  | 2.11 | DOB |  | 0.00 | DIPT |  | 0.00 | DIPT |
| 2.66 | 2C-B-fly |  | 1.79 | 2C-B-fly |  | 0.00 | 5-MeO-DIPT |  | 0.00 | 5-MeO-DIPT |
| 2.32 | Psilocin |  | 1.57 | 5-MeO-DMT |  | 0.00 | 2C-T-2 |  | 0.00 | 2C-T-2 |
| 2.05 | 5-MeO-DMT |  | 1.57 | Psilocin |  | 0.00 | DOET |  | 0.00 | DOET |
| 0.00 | MEM |  | 0.00 | MEM |  | 0.00 | DOB |  | 0.00 | DOB |
| 0.00 | TMA-2 |  | 0.00 | TMA-2 |  | 0.00 | 2C-B-fly |  | 0.00 | 2C-B-fly |
| ND | cis-2a |  | ND | cis-2a |  | 0.00 | 5-MeO-DMT |  | 0.00 | 5-MeO-DMT |
| ND | LSD |  | ND | LSD |  | 0.00 | Psilocin |  | 0.00 | Psilocin |
| ND | SS-2c |  | ND | SS-2c |  | 0.00 | MEM |  | 0.00 | MEM |
| ND | RR-2b |  | ND | RR-2b |  | 0.00 | TMA-2 |  | 0.00 | TMA-2 |
| ND | 2C-E |  | ND | 2C-E |  | 0.00 | 2C-E |  | 0.00 | 2C-E |
| ND | 2C-B |  | ND | 2C-B |  | 0.00 | 2C-B |  | 0.00 | 2C-B |
| ND | 5-MeO-TMT |  | ND | 5-MeO-TMT |  | 0.00 | Salvinorin A |  | 0.00 | Salvinorin A |
| ND | EMDT |  | ND | EMDT |  | ND | 5-MeO-TMT |  | ND | 5-MeO-TMT |
| ND | Salvinorin A |  | ND | Salvinorin A |  | ND | EMDT |  | ND | EMDT |
| ND | Ibogaine |  | ND | Ibogaine |  | ND | Ibogaine |  | ND | Ibogaine |
| ND | THC |  | ND | THC |  | ND | THC |  | ND | THC |
| ND | Morphine |  | ND | Morphine |  | ND | Morphine |  | ND | Morphine |

| α_2A_, α_2B_, α_2C_ | |  | α_2_max | |  | β_1_, β_2_ | |  | βmax | |
| --- | --- | --- | --- | --- | --- | --- | --- | --- | --- | --- |
| B_sq_ | Drug |  | npK_i_ | Drug |  | B_sq_ | Drug |  | npK_i_ | Drug |
| 6.30 | lisuride |  | 4.00 | Mescaline |  | 4.55 | DOI |  | 3.67 | 4C-T-2 |
| 5.70 | DOI |  | 3.88 | lisuride |  | 3.67 | 4C-T-2 |  | 3.52 | DOI |
| 5.70 | DMT |  | 3.79 | DOI |  | 3.38 | DOM |  | 3.38 | DOM |
| 5.50 | MDA |  | 3.60 | MDA |  | 3.21 | lisuride |  | 2.79 | Aleph-2 |
| 5.34 | MDMA |  | 3.53 | DMT |  | 2.81 | Aleph-2 |  | 2.56 | lisuride |
| 5.26 | 2C-E |  | 3.44 | 2C-E |  | 2.43 | DOB |  | 2.11 | DOB |
| 4.97 | DPT |  | 3.36 | TMA |  | 2.29 | DOET |  | 1.82 | DOET |
| 4.95 | Mescaline |  | 3.24 | 6-F-DMT |  | 1.90 | cis-2a |  | 1.76 | cis-2a |
| 4.69 | 2C-B |  | 3.21 | MDMA |  | 1.89 | 2C-T-2 |  | 1.72 | SS-2c |
| 4.49 | TMA |  | 3.12 | 2C-B |  | 1.82 | SS-2c |  | 1.70 | 2C-T-2 |
| 4.41 | 6-F-DMT |  | 2.97 | DPT |  | 1.74 | LSD |  | 1.39 | LSD |
| 4.10 | 5-MeO-MIPT |  | 2.85 | 5-MeO-MIPT |  | 0.00 | Mescaline |  | 0.00 | Mescaline |
| 3.78 | DOM |  | 2.68 | DIPT |  | 0.00 | MDA |  | 0.00 | MDA |
| 3.75 | DIPT |  | 2.66 | 5-MeO-DIPT |  | 0.00 | DMT |  | 0.00 | DMT |
| 3.74 | DOET |  | 2.56 | 2C-T-2 |  | 0.00 | TMA |  | 0.00 | TMA |
| 3.66 | 2C-T-2 |  | 2.40 | DOET |  | 0.00 | 6-F-DMT |  | 0.00 | 6-F-DMT |
| 3.59 | 5-MeO-DIPT |  | 2.30 | DOM |  | 0.00 | MDMA |  | 0.00 | MDMA |
| 2.66 | 2C-B-fly |  | 1.82 | DOB |  | 0.00 | DPT |  | 0.00 | DPT |
| 2.49 | DOB |  | 1.79 | 2C-B-fly |  | 0.00 | 5-MeO-MIPT |  | 0.00 | 5-MeO-MIPT |
| 2.32 | Psilocin |  | 1.57 | 5-MeO-DMT |  | 0.00 | DIPT |  | 0.00 | DIPT |
| 2.05 | 5-MeO-DMT |  | 1.57 | Psilocin |  | 0.00 | 5-MeO-DIPT |  | 0.00 | 5-MeO-DIPT |
| 1.37 | Aleph-2 |  | 1.06 | Aleph-2 |  | 0.00 | 2C-B-fly |  | 0.00 | 2C-B-fly |
| 0.00 | 4C-T-2 |  | 0.00 | 4C-T-2 |  | 0.00 | 5-MeO-DMT |  | 0.00 | 5-MeO-DMT |
| 0.00 | MEM |  | 0.00 | MEM |  | 0.00 | Psilocin |  | 0.00 | Psilocin |
| 0.00 | TMA-2 |  | 0.00 | TMA-2 |  | 0.00 | MEM |  | 0.00 | MEM |
| ND | cis-2a |  | ND | cis-2a |  | 0.00 | TMA-2 |  | 0.00 | TMA-2 |
| ND | LSD |  | ND | LSD |  | 0.00 | Salvinorin A |  | 0.00 | Salvinorin A |
| ND | SS-2c |  | ND | SS-2c |  | ND | 2C-E |  | ND | 2C-E |
| ND | RR-2b |  | ND | RR-2b |  | ND | 2C-B |  | ND | 2C-B |
| ND | Salvinorin A |  | ND | Salvinorin A |  | ND | RR-2b |  | ND | RR-2b |
| ND | 5-MeO-TMT |  | ND | 5-MeO-TMT |  | ND | 5-MeO-TMT |  | ND | 5-MeO-TMT |
| ND | EMDT |  | ND | EMDT |  | ND | EMDT |  | ND | EMDT |
| ND | Ibogaine |  | ND | Ibogaine |  | ND | Ibogaine |  | ND | Ibogaine |
| ND | THC |  | ND | THC |  | ND | THC |  | ND | THC |
| ND | Morphine |  | ND | Morphine |  | ND | Morphine |  | ND | Morphine |

| H_1_, H_2_ | |  | Hmax | |  | σ_1_, σ_2_ | |  | σmax | |
| --- | --- | --- | --- | --- | --- | --- | --- | --- | --- | --- |
| B_sq_ | Drug |  | npK_i_ | Drug |  | B_sq_ | Drug |  | npK_i_ | Drug |
| 4.47 | 6-F-DMT |  | 3.74 | 6-F-DMT |  | 5.58 | TMA |  | 4.00 | Ibogaine |
| 3.41 | DPT |  | 3.41 | DPT |  | 4.74 | Ibogaine |  | 3.95 | TMA |
| 3.04 | TMA-2 |  | 3.04 | TMA-2 |  | 3.87 | DIPT |  | 3.05 | 4C-T-2 |
| 2.53 | DIPT |  | 2.53 | DIPT |  | 3.55 | DPT |  | 2.90 | DPT |
| 2.42 | DOI |  | 2.42 | DOI |  | 3.41 | 5-MeO-DIPT |  | 2.83 | DIPT |
| 1.41 | 5-MeO-MIPT |  | 1.41 | 5-MeO-MIPT |  | 3.05 | 4C-T-2 |  | 2.64 | 5-MeO-DIPT |
| 0.63 | DOB |  | 0.63 | DOB |  | 2.42 | DOI |  | 2.23 | DMT |
| 0.19 | 2C-B-fly |  | 0.19 | 2C-B-fly |  | 2.23 | DMT |  | 2.13 | 5-MeO-MIPT |
| 0.00 | 4C-T-2 |  | 0.00 | 4C-T-2 |  | 2.21 | 6-F-DMT |  | 2.10 | MEM |
| 0.00 | DOM |  | 0.00 | DOM |  | 2.13 | 5-MeO-MIPT |  | 1.83 | Aleph-2 |
| 0.00 | Aleph-2 |  | 0.00 | Aleph-2 |  | 2.10 | MEM |  | 1.73 | DOI |
| 0.00 | DOET |  | 0.00 | DOET |  | 1.83 | Aleph-2 |  | 1.57 | 6-F-DMT |
| 0.00 | 2C-T-2 |  | 0.00 | 2C-T-2 |  | 1.66 | DOET |  | 1.25 | DOB |
| 0.00 | Mescaline |  | 0.00 | Mescaline |  | 1.25 | DOB |  | 1.19 | 2C-T-2 |
| 0.00 | TMA |  | 0.00 | TMA |  | 1.19 | 2C-T-2 |  | 1.18 | DOET |
| 0.00 | MDMA |  | 0.00 | MDMA |  | 0.00 | 2C-B-fly |  | 0.00 | 2C-B-fly |
| 0.00 | 5-MeO-DIPT |  | 0.00 | 5-MeO-DIPT |  | 0.00 | DOM |  | 0.00 | DOM |
| 0.00 | Psilocin |  | 0.00 | Psilocin |  | 0.00 | Mescaline |  | 0.00 | Mescaline |
| 0.00 | MEM |  | 0.00 | MEM |  | 0.00 | Psilocin |  | 0.00 | Psilocin |
| 0.00 | 2C-E |  | 0.00 | 2C-E |  | 0.00 | 2C-B |  | 0.00 | 2C-B |
| 0.00 | 2C-B |  | 0.00 | 2C-B |  | 0.00 | 5-MeO-DMT |  | 0.00 | 5-MeO-DMT |
| 0.00 | 5-MeO-TMT |  | 0.00 | 5-MeO-TMT |  | ND | TMA-2 |  | ND | TMA-2 |
| 0.00 | EMDT |  | 0.00 | EMDT |  | ND | MDMA |  | ND | MDMA |
| ND | lisuride |  | ND | lisuride |  | ND | 2C-E |  | ND | 2C-E |
| ND | cis-2a |  | ND | cis-2a |  | ND | 5-MeO-TMT |  | ND | 5-MeO-TMT |
| ND | SS-2c |  | ND | SS-2c |  | ND | EMDT |  | ND | EMDT |
| ND | LSD |  | ND | LSD |  | ND | lisuride |  | ND | lisuride |
| ND | MDA |  | ND | MDA |  | ND | cis-2a |  | ND | cis-2a |
| ND | DMT |  | ND | DMT |  | ND | SS-2c |  | ND | SS-2c |
| ND | 5-MeO-DMT |  | ND | 5-MeO-DMT |  | ND | LSD |  | ND | LSD |
| ND | Salvinorin A |  | ND | Salvinorin A |  | ND | MDA |  | ND | MDA |
| ND | RR-2b |  | ND | RR-2b |  | ND | Salvinorin A |  | ND | Salvinorin A |
| ND | Ibogaine |  | ND | Ibogaine |  | ND | RR-2b |  | ND | RR-2b |
| ND | THC |  | ND | THC |  | ND | THC |  | ND | THC |
| ND | Morphine |  | ND | Morphine |  | ND | Morphine |  | ND | Morphine |

| M_1_, M_2_, M_3_, M_4_, M_5_ | |  | Mmax | |  | SERT, DAT, NET | |  | TransporterMax | |
| --- | --- | --- | --- | --- | --- | --- | --- | --- | --- | --- |
| B_sq_ | Drug |  | npK_i_ | Drug |  | B_sq_ | Drug |  | npK_i_ | Drug |
| 5.53 | DOI |  | 3.07 | MDMA |  | 4.04 | DPT |  | 3.31 | DPT |
| 4.67 | MDMA |  | 2.90 | DOI |  | 3.25 | 6-F-DMT |  | 3.25 | 6-F-DMT |
| 2.94 | 2C-E |  | 2.22 | 2C-B |  | 2.98 | DIPT |  | 2.98 | DIPT |
| 2.51 | 2C-B-fly |  | 2.16 | 2C-E |  | 2.83 | DOI |  | 2.83 | DOI |
| 2.51 | 2C-T-2 |  | 1.94 | 2C-T-2 |  | 2.72 | 5-MeO-DIPT |  | 2.72 | 5-MeO-DIPT |
| 2.22 | 2C-B |  | 1.53 | DOB |  | 2.37 | DMT |  | 2.37 | DMT |
| 1.53 | DOB |  | 1.42 | 2C-B-fly |  | 1.97 | 5-MeO-TMT |  | 1.97 | 5-MeO-TMT |
| 0.97 | Aleph-2 |  | 0.92 | Aleph-2 |  | 1.74 | Psilocin |  | 1.74 | Psilocin |
| 0.00 | 4C-T-2 |  | 0.00 | 4C-T-2 |  | 1.28 | 5-MeO-MIPT |  | 1.28 | 5-MeO-MIPT |
| 0.00 | DPT |  | 0.00 | DPT |  | 1.27 | 5-MeO-DMT |  | 1.08 | Aleph-2 |
| 0.00 | DIPT |  | 0.00 | DIPT |  | 1.08 | Aleph-2 |  | 0.97 | 5-MeO-DMT |
| 0.00 | 5-MeO-DIPT |  | 0.00 | 5-MeO-DIPT |  | 0.66 | DOB |  | 0.66 | DOB |
| 0.00 | DMT |  | 0.00 | DMT |  | 0.00 | MDMA |  | 0.00 | MDMA |
| 0.00 | 5-MeO-MIPT |  | 0.00 | 5-MeO-MIPT |  | 0.00 | 2C-B |  | 0.00 | 2C-B |
| 0.00 | MEM |  | 0.00 | MEM |  | 0.00 | 2C-E |  | 0.00 | 2C-E |
| 0.00 | 6-F-DMT |  | 0.00 | 6-F-DMT |  | 0.00 | 2C-T-2 |  | 0.00 | 2C-T-2 |
| 0.00 | Mescaline |  | 0.00 | Mescaline |  | 0.00 | 2C-B-fly |  | 0.00 | 2C-B-fly |
| 0.00 | 5-MeO-DMT |  | 0.00 | 5-MeO-DMT |  | 0.00 | 4C-T-2 |  | 0.00 | 4C-T-2 |
| 0.00 | lisuride |  | 0.00 | lisuride |  | 0.00 | MEM |  | 0.00 | MEM |
| 0.00 | Salvinorin A |  | 0.00 | Salvinorin A |  | 0.00 | Mescaline |  | 0.00 | Mescaline |
| ND | Ibogaine |  | ND | Ibogaine |  | 0.00 | lisuride |  | 0.00 | lisuride |
| ND | TMA |  | ND | TMA |  | 0.00 | TMA |  | 0.00 | TMA |
| ND | DOET |  | ND | DOET |  | 0.00 | DOET |  | 0.00 | DOET |
| ND | DOM |  | ND | DOM |  | 0.00 | DOM |  | 0.00 | DOM |
| ND | Psilocin |  | ND | Psilocin |  | 0.00 | TMA-2 |  | 0.00 | TMA-2 |
| ND | TMA-2 |  | ND | TMA-2 |  | 0.00 | EMDT |  | 0.00 | EMDT |
| ND | 5-MeO-TMT |  | ND | 5-MeO-TMT |  | 0.00 | cis-2a |  | 0.00 | cis-2a |
| ND | EMDT |  | ND | EMDT |  | 0.00 | SS-2c |  | 0.00 | SS-2c |
| ND | cis-2a |  | ND | cis-2a |  | 0.00 | LSD |  | 0.00 | LSD |
| ND | SS-2c |  | ND | SS-2c |  | 0.00 | MDA |  | 0.00 | MDA |
| ND | LSD |  | ND | LSD |  | 0.00 | RR-2b |  | 0.00 | RR-2b |
| ND | MDA |  | ND | MDA |  | ND | Salvinorin A |  | ND | Salvinorin A |
| ND | RR-2b |  | ND | RR-2b |  | ND | Ibogaine |  | ND | Ibogaine |
| ND | THC |  | ND | THC |  | ND | THC |  | ND | THC |
| ND | Morphine |  | ND | Morphine |  | ND | Morphine |  | ND | Morphine |

| DOR, KOR, MOR | |  | OpioidMax | |  | CB_1_, CB_2_ | |
| --- | --- | --- | --- | --- | --- | --- | --- |
| B_sq_ | Drug |  | npK_i_ | Drug |  | B_sq_ | Drug |
| 4.63 | Morphine |  | 4.00 | Morphine |  | 5.50 | THC |
| 4.00 | Salvinorin A |  | 4.00 | Salvinorin A |  | 0.00 | 2C-B-fly |
| 3.93 | Ibogaine |  | 2.88 | Ibogaine |  | 0.00 | DPT |
| 0.00 | DPT |  | 0.00 | DPT |  | 0.00 | DIPT |
| 0.00 | DIPT |  | 0.00 | DIPT |  | 0.00 | DOI |
| 0.00 | DOI |  | 0.00 | DOI |  | 0.00 | 5-MeO-DIPT |
| 0.00 | 5-MeO-DIPT |  | 0.00 | 5-MeO-DIPT |  | 0.00 | Psilocin |
| 0.00 | 5-MeO-TMT |  | 0.00 | 5-MeO-TMT |  | 0.00 | Aleph-2 |
| 0.00 | Psilocin |  | 0.00 | Psilocin |  | 0.00 | DOB |
| 0.00 | 5-MeO-MIPT |  | 0.00 | 5-MeO-MIPT |  | 0.00 | 2C-B |
| 0.00 | Aleph-2 |  | 0.00 | Aleph-2 |  | 0.00 | 2C-E |
| 0.00 | DOB |  | 0.00 | DOB |  | 0.00 | 2C-T-2 |
| 0.00 | 2C-B |  | 0.00 | 2C-B |  | 0.00 | 4C-T-2 |
| 0.00 | 2C-E |  | 0.00 | 2C-E |  | 0.00 | MEM |
| 0.00 | 2C-T-2 |  | 0.00 | 2C-T-2 |  | 0.00 | Mescaline |
| 0.00 | 2C-B-fly |  | 0.00 | 2C-B-fly |  | 0.00 | TMA |
| 0.00 | 4C-T-2 |  | 0.00 | 4C-T-2 |  | 0.00 | DOET |
| 0.00 | MEM |  | 0.00 | MEM |  | 0.00 | DOM |
| 0.00 | Mescaline |  | 0.00 | Mescaline |  | 0.00 | 6-F-DMT |
| 0.00 | TMA |  | 0.00 | TMA |  | 0.00 | DMT |
| 0.00 | DOET |  | 0.00 | DOET |  | 0.00 | 5-MeO-DMT |
| 0.00 | DOM |  | 0.00 | DOM |  | 0.00 | lisuride |
| 0.00 | EMDT |  | 0.00 | EMDT |  | 0.00 | MDA |
| 0.00 | cis-2a |  | 0.00 | cis-2a |  | ND | Morphine |
| 0.00 | SS-2c |  | 0.00 | SS-2c |  | ND | Salvinorin A |
| 0.00 | LSD |  | 0.00 | LSD |  | ND | Ibogaine |
| 0.00 | RR-2b |  | 0.00 | RR-2b |  | ND | 5-MeO-TMT |
| ND | 6-F-DMT |  | ND | 6-F-DMT |  | ND | 5-MeO-MIPT |
| ND | DMT |  | ND | DMT |  | ND | EMDT |
| ND | 5-MeO-DMT |  | ND | 5-MeO-DMT |  | ND | cis-2a |
| ND | MDMA |  | ND | MDMA |  | ND | SS-2c |
| ND | lisuride |  | ND | lisuride |  | ND | LSD |
| ND | TMA-2 |  | ND | TMA-2 |  | ND | RR-2b |
| ND | MDA |  | ND | MDA |  | ND | MDMA |
| ND | THC |  | ND | THC |  | ND | TMA-2 |
