# Supplementary material for: Psychedelics and the Human Receptorome
Source: PLoS One. 2010 Feb 2;5(2):e9019. doi: 10.1371/journal.pone.0009019 (PMC2814854; doi:10.1371/journal.pone.0009019)
Supplement: Table S7 — Thirty-five drugs arranged in order of decreasing proportional interaction at selected groups of receptors. The thirty-five drugs are arranged in order of decreasing proportional interaction at groups of receptors, based on the proportional breadth index Bp. The drugs with the greatest proportional interactions are found at the tops of the columns, and the drugs with the least proportional interactions are found at the bottoms of the columns. An entry of “ND” indicates that the statistic could not be calculated because some data is missing. The same data is also presented in Table S8. Receptors in a group are listed in the column heading, or are represented with the following abbreviations: • 5-HT - 5-HT1A, 5-HT1B, 5-HT1D, 5-HT1E, 5-HT2A, 5-HT2B, 5-HT2C, 5-HT5A, 5-HT6, 5-HT7 • 5-HT1 - 5-HT1A, 5-HT1B, 5-HT1D, 5-HT1E • Adrenergic - α1A, α1B, α2A, α2B, α2C, β1, β2 (0.37 MB DOC) [file pone.0009019.s010.doc]

| 5-HT | |  | 5-HT_1_ | |  | 5-HT_2A_, 5-HT_2C_ | |  | 5-HT_2A_, 5-HT_2B_, 5-HT_2C_ | |  | 5-HT_6_, 5-HT_7_ | |
| --- | --- | --- | --- | --- | --- | --- | --- | --- | --- | --- | --- | --- | --- |
| B_p_ | Drug |  | B_p_ | Drug |  | B_p_ | Drug |  | B_p_ | Drug |  | B_p_ | Drug |
| 0.848 | MEM |  | 0.455 | 5-MeO-DMT |  | 0.421 | TMA-2 |  | 0.788 | TMA-2 |  | 0.449 | EMDT |
| 0.820 | 2C-B-fly |  | 0.446 | RR-2b |  | 0.272 | DOET |  | 0.717 | MEM |  | 0.411 | 5-MeO-TMT |
| 0.804 | 5-MeO-DMT |  | 0.324 | 5-MeO-MIPT |  | 0.271 | DOB |  | 0.528 | Aleph-2 |  | 0.260 | 5-MeO-DMT |
| 0.788 | TMA-2 |  | 0.322 | 2C-B-fly |  | 0.228 | Aleph-2 |  | 0.495 | DOB |  | 0.192 | 5-MeO-MIPT |
| 0.784 | RR-2b |  | 0.318 | TMA |  | 0.225 | 2C-B-fly |  | 0.438 | 2C-B-fly |  | 0.168 | cis-2a |
| 0.747 | DOET |  | 0.312 | 2C-B |  | 0.222 | 4C-T-2 |  | 0.430 | DOET |  | 0.164 | LSD |
| 0.738 | Psilocin |  | 0.311 | 5-MeO-DIPT |  | 0.208 | 2C-T-2 |  | 0.395 | 4C-T-2 |  | 0.153 | SS-2c |
| 0.734 | 2C-B |  | 0.306 | Mescaline |  | 0.190 | 2C-E |  | 0.380 | 2C-T-2 |  | 0.151 | 6-F-DMT |
| 0.719 | LSD |  | 0.295 | Psilocin |  | 0.184 | 2C-B |  | 0.345 | DOM |  | 0.149 | DMT |
| 0.718 | 5-MeO-MIPT |  | 0.293 | LSD |  | 0.168 | MEM |  | 0.331 | MDA |  | 0.148 | RR-2b |
| 0.696 | DOB |  | 0.273 | 2C-E |  | 0.160 | DOI |  | 0.309 | 2C-E |  | 0.138 | Psilocin |
| 0.668 | 2C-T-2 |  | 0.268 | DOET |  | 0.129 | LSD |  | 0.307 | 2C-B |  | 0.131 | MEM |
| 0.666 | 2C-E |  | 0.225 | 2C-T-2 |  | 0.122 | SS-2c |  | 0.235 | Psilocin |  | 0.114 | 2C-B |
| 0.622 | Aleph-2 |  | 0.214 | DIPT |  | 0.112 | DOM |  | 0.216 | DOI |  | 0.108 | TMA |
| 0.615 | TMA |  | 0.195 | DPT |  | 0.108 | cis-2a |  | 0.210 | Mescaline |  | 0.098 | lisuride |
| 0.595 | 4C-T-2 |  | 0.190 | 6-F-DMT |  | 0.103 | DMT |  | 0.189 | DMT |  | 0.095 | 5-MeO-DIPT |
| 0.591 | DOM |  | 0.176 | DOM |  | 0.095 | Psilocin |  | 0.188 | TMA |  | 0.093 | MDA |
| 0.563 | 5-MeO-DIPT |  | 0.174 | lisuride |  | 0.074 | 5-MeO-MIPT |  | 0.185 | LSD |  | 0.085 | 2C-E |
| 0.544 | DMT |  | 0.149 | DMT |  | 0.074 | MDA |  | 0.182 | SS-2c |  | 0.072 | DPT |
| 0.516 | Mescaline |  | 0.130 | DOB |  | 0.073 | EMDT |  | 0.166 | cis-2a |  | 0.070 | DOM |
| 0.515 | MDA |  | 0.123 | DOI |  | 0.068 | TMA |  | 0.166 | 5-MeO-MIPT |  | 0.063 | 2C-T-2 |
| 0.510 | 6-F-DMT |  | 0.091 | MDA |  | 0.067 | 5-MeO-TMT |  | 0.158 | 5-MeO-DIPT |  | 0.062 | DIPT |
| 0.453 | lisuride |  | 0.078 | 4C-T-2 |  | 0.063 | 6-F-DMT |  | 0.147 | MDMA |  | 0.060 | 2C-B-fly |
| 0.426 | DPT |  | 0.062 | Aleph-2 |  | 0.055 | DPT |  | 0.140 | 6-F-DMT |  | 0.060 | DOB |
| 0.392 | DIPT |  | 0.000 | MEM |  | 0.054 | Ibogaine |  | 0.140 | DPT |  | 0.052 | DOI |
| 0.391 | DOI |  | 0.000 | TMA-2 |  | 0.053 | lisuride |  | 0.118 | lisuride |  | 0.051 | 4C-T-2 |
| 0.147 | MDMA |  | 0.000 | MDMA |  | 0.050 | RR-2b |  | 0.116 | DIPT |  | 0.049 | DOET |
| 0.000 | Salvinorin A |  | 0.000 | Salvinorin A |  | 0.041 | 5-MeO-DMT |  | 0.084 | RR-2b |  | 0.032 | Aleph-2 |
| ND | 5-MeO-TMT |  | ND | 5-MeO-TMT |  | 0.000 | 5-MeO-DIPT |  | 0.047 | 5-MeO-DMT |  | 0.000 | TMA-2 |
| ND | SS-2c |  | ND | SS-2c |  | 0.000 | Mescaline |  | 0.000 | Salvinorin A |  | 0.000 | Mescaline |
| ND | EMDT |  | ND | EMDT |  | 0.000 | DIPT |  | ND | EMDT |  | 0.000 | MDMA |
| ND | THC |  | ND | THC |  | 0.000 | MDMA |  | ND | 5-MeO-TMT |  | 0.000 | Salvinorin_A |
| ND | Morphine |  | ND | Morphine |  | 0.000 | Salvinorin A |  | ND | Ibogaine |  | ND | THC |
| ND | Ibogaine |  | ND | Ibogaine |  | ND | THC |  | ND | THC |  | ND | Morphine |
| ND | cis-2a |  | ND | cis-2a |  | ND | Morphine |  | ND | Morphine |  | ND | Ibogaine |

| Adrenergic | |  | α_1A_, α_1B_ | |  | α_2A_, α_2B_, α_2C_ | |  | β_1_, β_2_ | |
| --- | --- | --- | --- | --- | --- | --- | --- | --- | --- | --- |
| B_p_ | Drug |  | B_p_ | Drug |  | B_p_ | Drug |  | B_p_ | Drug |
| 0.485 | MDA |  | 0.090 | DMT |  | 0.485 | MDA |  | 0.166 | DOM |
| 0.409 | DOM |  | 0.083 | DPT |  | 0.326 | Mescaline |  | 0.149 | Aleph-2 |
| 0.393 | lisuride |  | 0.050 | 6-F-DMT |  | 0.316 | MDMA |  | 0.145 | 4C-T-2 |
| 0.326 | Mescaline |  | 0.039 | lisuride |  | 0.281 | lisuride |  | 0.119 | DOI |
| 0.316 | MDMA |  | 0.035 | DOM |  | 0.207 | DOM |  | 0.083 | DOB |
| 0.305 | DOI |  | 0.021 | cis-2a |  | 0.206 | 2C-E |  | 0.073 | lisuride |
| 0.273 | DMT |  | 0.016 | LSD |  | 0.186 | DOI |  | 0.061 | DOET |
| 0.223 | DPT |  | 0.006 | SS-2c |  | 0.183 | DMT |  | 0.042 | SS-2c |
| 0.221 | DOET |  | 0.004 | RR-2b |  | 0.170 | 2C-B |  | 0.038 | 2C-T-2 |
| 0.184 | Aleph-2 |  | 0.000 | MDA |  | 0.161 | DOET |  | 0.028 | cis-2a |
| 0.182 | 2C-T-2 |  | 0.000 | Mescaline |  | 0.151 | TMA |  | 0.018 | LSD |
| 0.170 | DOB |  | 0.000 | MDMA |  | 0.144 | 2C-T-2 |  | 0.000 | MDA |
| 0.151 | TMA |  | 0.000 | DOI |  | 0.140 | DPT |  | 0.000 | Mescaline |
| 0.146 | 6-F-DMT |  | 0.000 | DOET |  | 0.139 | 5-MeO-MIPT |  | 0.000 | MDMA |
| 0.145 | 4C-T-2 |  | 0.000 | Aleph-2 |  | 0.135 | DIPT |  | 0.000 | DMT |
| 0.139 | 5-MeO-MIPT |  | 0.000 | 2C-T-2 |  | 0.133 | 5-MeO-DIPT |  | 0.000 | TMA |
| 0.135 | DIPT |  | 0.000 | DOB |  | 0.096 | 6-F-DMT |  | 0.000 | DPT |
| 0.133 | 5-MeO-DIPT |  | 0.000 | TMA |  | 0.094 | 2C-B-fly |  | 0.000 | 5-MeO-MIPT |
| 0.094 | 2C-B-fly |  | 0.000 | 4C-T-2 |  | 0.087 | DOB |  | 0.000 | DIPT |
| 0.052 | 5-MeO-DMT |  | 0.000 | 5-MeO-MIPT |  | 0.052 | 5-MeO-DMT |  | 0.000 | 5-MeO-DIPT |
| 0.047 | Psilocin |  | 0.000 | DIPT |  | 0.047 | Psilocin |  | 0.000 | 6-F-DMT |
| 0.000 | MEM |  | 0.000 | 5-MeO-DIPT |  | 0.035 | Aleph-2 |  | 0.000 | 2C-B-fly |
| 0.000 | TMA-2 |  | 0.000 | 2C-B-fly |  | 0.000 | 4C-T-2 |  | 0.000 | 5-MeO-DMT |
| ND | EMDT |  | 0.000 | 5-MeO-DMT |  | 0.000 | MEM |  | 0.000 | Psilocin |
| ND | 5-MeO-TMT |  | 0.000 | Psilocin |  | 0.000 | TMA-2 |  | 0.000 | MEM |
| ND | RR-2b |  | 0.000 | MEM |  | ND | cis-2a |  | 0.000 | TMA-2 |
| ND | cis-2a |  | 0.000 | TMA-2 |  | ND | LSD |  | 0.000 | Salvinorin A |
| ND | LSD |  | 0.000 | 2C-B |  | ND | SS-2c |  | ND | 2C-E |
| ND | SS-2c |  | 0.000 | 2C-E |  | ND | RR-2b |  | ND | 2C-B |
| ND | 2C-B |  | 0.000 | Salvinorin A |  | ND | Salvinorin A |  | ND | RR-2b |
| ND | 2C-E |  | ND | EMDT |  | ND | EMDT |  | ND | EMDT |
| ND | Salvinorin A |  | ND | 5-MeO-TMT |  | ND | 5-MeO-TMT |  | ND | 5-MeO-TMT |
| ND | Ibogaine |  | ND | Ibogaine |  | ND | Ibogaine |  | ND | Ibogaine |
| ND | THC |  | ND | THC |  | ND | THC |  | ND | THC |
| ND | Morphine |  | ND | Morphine |  | ND | Morphine |  | ND | Morphine |

| D_1_, D_2_, D_3_, D_4_, D_5_ | |  | M_1_, M_2_, M_3_, M_4_, M_5_ | |  | H_1_, H_2_ | |  | σ_1_, σ_2_ | |
| --- | --- | --- | --- | --- | --- | --- | --- | --- | --- | --- |
| B_p_ | Drug |  | B_p_ | Drug |  | B_p_ | Drug |  | B_p_ | Drug |
| 0.244 | cis-2a |  | 0.242 | MDMA |  | 0.212 | TMA-2 |  | 0.257 | Ibogaine |
| 0.203 | SS-2c |  | 0.175 | DOI |  | 0.099 | 6-F-DMT |  | 0.234 | TMA |
| 0.199 | RR-2b |  | 0.084 | 2C-B-fly |  | 0.066 | DPT |  | 0.152 | MEM |
| 0.185 | LSD |  | 0.068 | 2C-T-2 |  | 0.061 | DIPT |  | 0.144 | DIPT |
| 0.161 | Psilocin |  | 0.064 | 2C-E |  | 0.033 | DOI |  | 0.120 | 5-MeO-DIPT |
| 0.140 | lisuride |  | 0.038 | 2C-B |  | 0.016 | 5-MeO-MIPT |  | 0.100 | 4C-T-2 |
| 0.138 | 6-F-DMT |  | 0.033 | DOB |  | 0.006 | DOB |  | 0.071 | DPT |
| 0.115 | 5-MeO-DMT |  | 0.018 | Aleph-2 |  | 0.001 | 2C-B-fly |  | 0.063 | Aleph-2 |
| 0.076 | 4C-T-2 |  | 0.000 | lisuride |  | 0.000 | MDMA |  | 0.037 | 5-MeO-MIPT |
| 0.072 | DPT |  | 0.000 | 6-F-DMT |  | 0.000 | 2C-T-2 |  | 0.034 | DOI |
| 0.069 | DMT |  | 0.000 | 5-MeO-DMT |  | 0.000 | 2C-E |  | 0.032 | DOET |
| 0.064 | 2C-E |  | 0.000 | 4C-T-2 |  | 0.000 | 2C-B |  | 0.028 | DMT |
| 0.063 | DIPT |  | 0.000 | DPT |  | 0.000 | Aleph-2 |  | 0.024 | 6-F-DMT |
| 0.044 | 2C-T-2 |  | 0.000 | DMT |  | 0.000 | 4C-T-2 |  | 0.022 | DOB |
| 0.040 | DOB |  | 0.000 | DIPT |  | 0.000 | Mescaline |  | 0.015 | 2C-T-2 |
| 0.038 | 5-MeO-MIPT |  | 0.000 | 5-MeO-MIPT |  | 0.000 | 5-MeO-DIPT |  | 0.000 | 2C-B-fly |
| 0.037 | Aleph-2 |  | 0.000 | Mescaline |  | 0.000 | MEM |  | 0.000 | 2C-B |
| 0.033 | 2C-B |  | 0.000 | 5-MeO-DIPT |  | 0.000 | Psilocin |  | 0.000 | Mescaline |
| 0.016 | DOI |  | 0.000 | MEM |  | 0.000 | DOM |  | 0.000 | Psilocin |
| 0.000 | DOM |  | 0.000 | Salvinorin A |  | 0.000 | DOET |  | 0.000 | DOM |
| 0.000 | DOET |  | ND | cis-2a |  | 0.000 | TMA |  | 0.000 | 5-MeO-DMT |
| 0.000 | MDA |  | ND | SS-2c |  | 0.000 | EMDT |  | ND | TMA-2 |
| 0.000 | Mescaline |  | ND | RR-2b |  | 0.000 | 5-MeO-TMT |  | ND | MDMA |
| 0.000 | MDMA |  | ND | LSD |  | ND | lisuride |  | ND | 2C-E |
| 0.000 | TMA |  | ND | Psilocin |  | ND | 5-MeO-DMT |  | ND | EMDT |
| 0.000 | 5-MeO-DIPT |  | ND | DOM |  | ND | DMT |  | ND | 5-MeO-TMT |
| 0.000 | MEM |  | ND | DOET |  | ND | Salvinorin A |  | ND | lisuride |
| 0.000 | TMA-2 |  | ND | MDA |  | ND | cis-2a |  | ND | Salvinorin A |
| 0.000 | Salvinorin A |  | ND | TMA |  | ND | SS-2c |  | ND | cis-2a |
| 0.000 | EMDT |  | ND | TMA-2 |  | ND | RR-2b |  | ND | SS-2c |
| 0.000 | 5-MeO-TMT |  | ND | EMDT |  | ND | LSD |  | ND | RR-2b |
| ND | 2C-B-fly |  | ND | 5-MeO-TMT |  | ND | MDA |  | ND | LSD |
| ND | Ibogaine |  | ND | Ibogaine |  | ND | Ibogaine |  | ND | MDA |
| ND | THC |  | ND | THC |  | ND | THC |  | ND | THC |
| ND | Morphine |  | ND | Morphine |  | ND | Morphine |  | ND | Morphine |

| SERT, NET, DAT | |  | DOR, KOR, MOR | |  | CB_1_, CB_2_ | |
| --- | --- | --- | --- | --- | --- | --- | --- |
| B_p_ | Drug |  | B_p_ | Drug |  | B_p_ | Drug |
| 0.092 | DPT |  | 1.000 | Salvinorin A |  | 1.000 | THC |
| 0.085 | DIPT |  | 1.000 | Morphine |  | 0.000 | 2C-B-fly |
| 0.076 | 5-MeO-DIPT |  | 0.177 | Ibogaine |  | 0.000 | DPT |
| 0.055 | 5-MeO-TMT |  | 0.000 | DPT |  | 0.000 | DIPT |
| 0.052 | 6-F-DMT |  | 0.000 | DIPT |  | 0.000 | 5-MeO-DIPT |
| 0.046 | DOI |  | 0.000 | 5-MeO-DIPT |  | 0.000 | DOI |
| 0.032 | DMT |  | 0.000 | 5-MeO-TMT |  | 0.000 | Psilocin |
| 0.026 | Psilocin |  | 0.000 | DOI |  | 0.000 | Aleph-2 |
| 0.022 | Aleph-2 |  | 0.000 | Psilocin |  | 0.000 | DOB |
| 0.020 | 5-MeO-DMT |  | 0.000 | Aleph-2 |  | 0.000 | TMA |
| 0.014 | 5-MeO-MIPT |  | 0.000 | 5-MeO-MIPT |  | 0.000 | MEM |
| 0.006 | DOB |  | 0.000 | DOB |  | 0.000 | 4C-T-2 |
| 0.000 | TMA |  | 0.000 | TMA |  | 0.000 | DOET |
| 0.000 | MEM |  | 0.000 | MEM |  | 0.000 | 2C-T-2 |
| 0.000 | 4C-T-2 |  | 0.000 | 4C-T-2 |  | 0.000 | 2C-B |
| 0.000 | DOET |  | 0.000 | DOET |  | 0.000 | Mescaline |
| 0.000 | 2C-T-2 |  | 0.000 | 2C-T-2 |  | 0.000 | DOM |
| 0.000 | 2C-B-fly |  | 0.000 | 2C-B-fly |  | 0.000 | 2C-E |
| 0.000 | 2C-B |  | 0.000 | 2C-B |  | 0.000 | 6-F-DMT |
| 0.000 | Mescaline |  | 0.000 | Mescaline |  | 0.000 | DMT |
| 0.000 | DOM |  | 0.000 | DOM |  | 0.000 | 5-MeO-DMT |
| 0.000 | TMA-2 |  | 0.000 | 2C-E |  | 0.000 | lisuride |
| 0.000 | MDMA |  | 0.000 | EMDT |  | 0.000 | MDA |
| 0.000 | 2C-E |  | 0.000 | cis-2a |  | ND | Salvinorin A |
| 0.000 | EMDT |  | 0.000 | SS-2c |  | ND | Morphine |
| 0.000 | lisuride |  | 0.000 | RR-2b |  | ND | Ibogaine |
| 0.000 | cis-2a |  | 0.000 | LSD |  | ND | 5-MeO-TMT |
| 0.000 | SS-2c |  | ND | 6-F-DMT |  | ND | 5-MeO-MIPT |
| 0.000 | RR-2b |  | ND | DMT |  | ND | EMDT |
| 0.000 | LSD |  | ND | 5-MeO-DMT |  | ND | cis-2a |
| 0.000 | MDA |  | ND | TMA-2 |  | ND | SS-2c |
| ND | Ibogaine |  | ND | MDMA |  | ND | RR-2b |
| ND | Salvinorin A |  | ND | lisuride |  | ND | LSD |
| ND | THC |  | ND | MDA |  | ND | TMA-2 |
| ND | Morphine |  | ND | THC |  | ND | MDMA |
