# Supplementary material for: Psychedelics and the Human Receptorome
Source: PLoS One. 2010 Feb 2;5(2):e9019. doi: 10.1371/journal.pone.0009019 (PMC2814854; doi:10.1371/journal.pone.0009019)
Supplement: Supplementary file 8 [file pone.0009019.s001.doc]

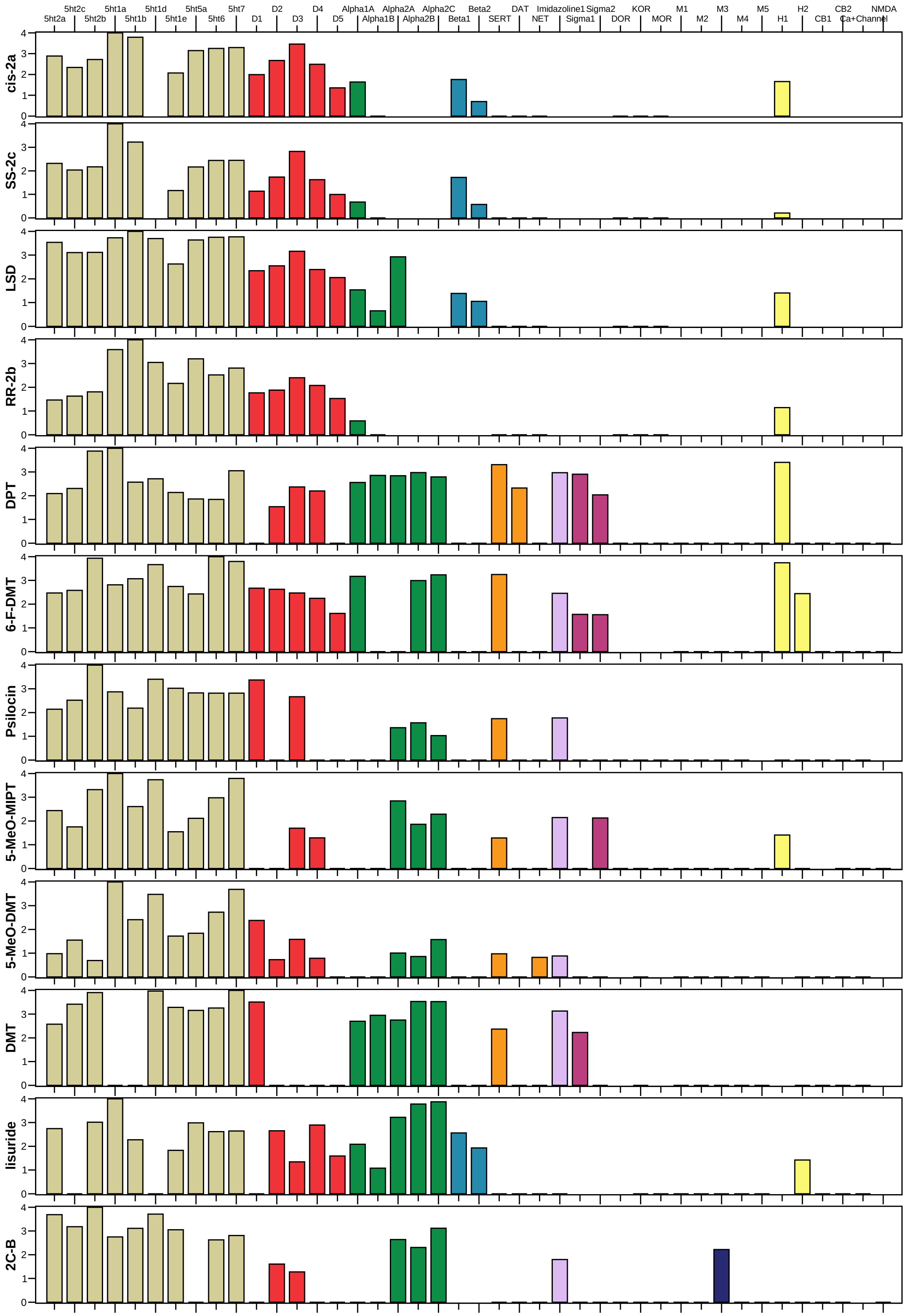
5ht2a 5ht2c 5ht2b 5ht1a 5ht1b 5ht1d 5ht1e 5ht5a 5ht6 5ht7 D1 D2 D3 D4 D5 Alpha1A Alpha1B Alpha2A Alpha2B Alpha2C Beta1 Beta2 SERT DAT NET Imidazoline1 Sigma1 Sigma2 DOR KOR MOR M1 M2 M3 M4 M5 H1 H2 CB1 CB2 Ca+Channel NMDA
cis-2a
SS-2c
LSD
RR-2b
DPT
6-F-DMT
Psilocin
5-MeO-MIPT
5-MeO-DMT
DMT
lisuride
2C-B
0
1
2
3
4

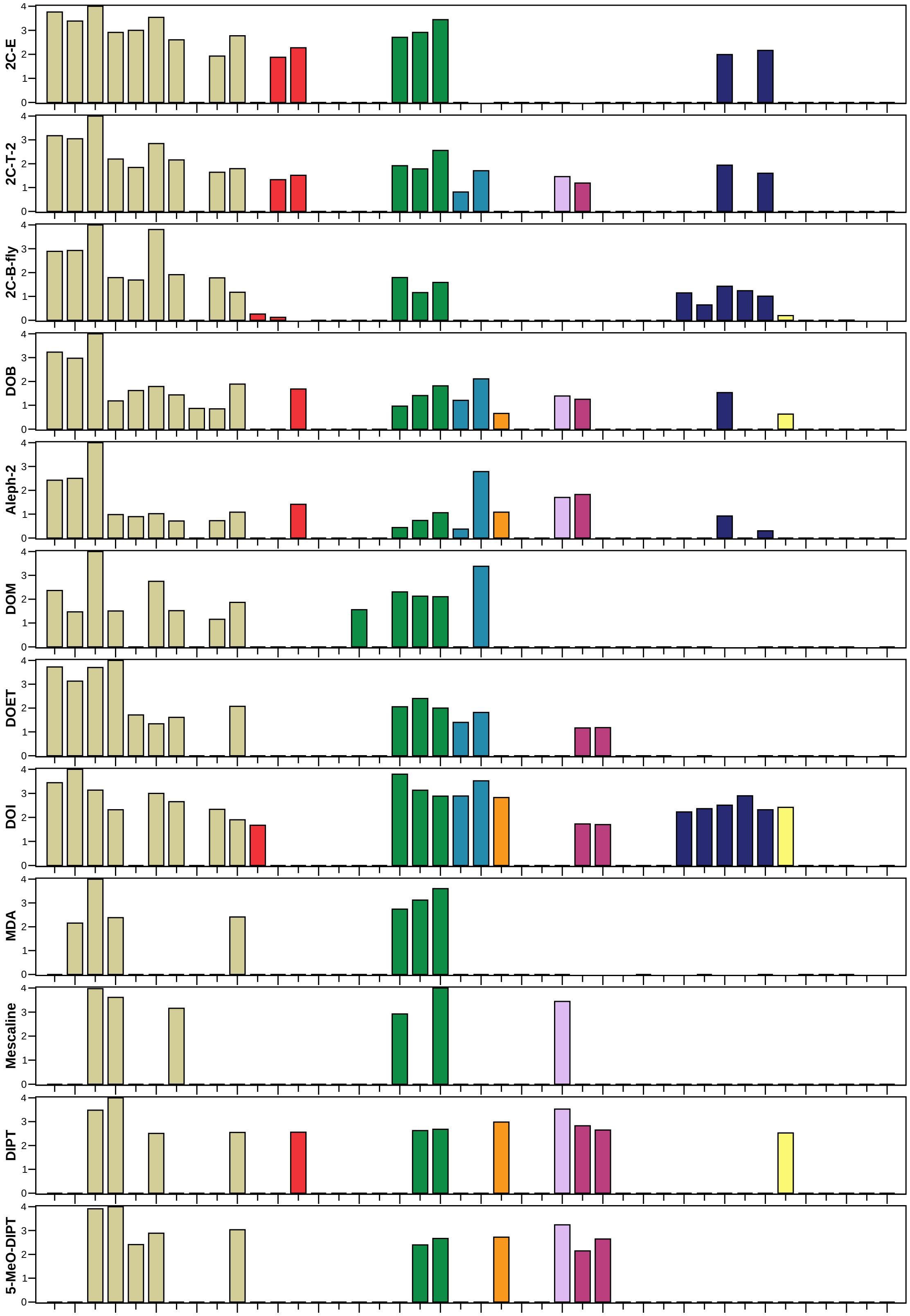
2C-E
2C-T-2
2C-B-fly
DOB
Aleph-2
DOM
DOET
DOI
MDA
Mescaline
DIPT
5-MeO-DIPT
0
1
2
3
4

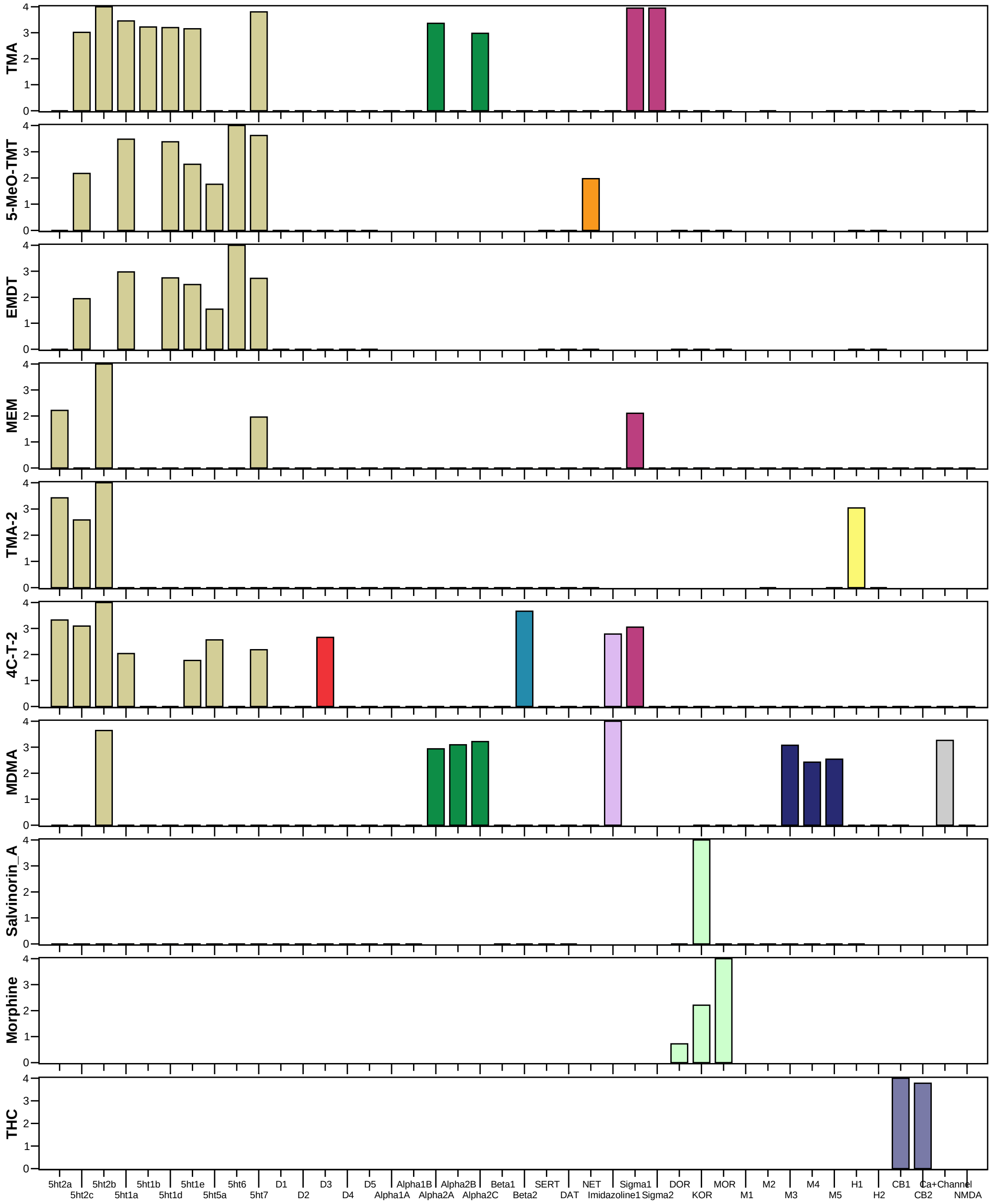

TMA
5-MeO-TMT
EMDT
MEM
TMA-2
4C-T-2
MDMA
Salvinorin_A
Morphine
THC
0
1
2
3
4
5ht2a
5ht2c
5ht2b
5ht1a
5ht1b
5ht1d
5ht1e
5ht5a
5ht6
5ht7
D1
D2
D3
D4
D5
Alpha1A
Alpha1B
Alpha2A
Alpha2B
Alpha2C
Beta1
Beta2
SERT
DAT
NET
Imidazoline1
Sigma1
Sigma2
DOR
KOR
MOR
M1
M2
M3
M4
M5
H1
H2
CB1
CB2
Ca+Channel
NMDA
