# Supplementary material for: Psychedelics and the Human Receptorome
Source: PLoS One. 2010 Feb 2;5(2):e9019. doi: 10.1371/journal.pone.0009019 (PMC2814854; doi:10.1371/journal.pone.0009019)
Supplement: Supplementary file 9 [file pone.0009019.s002.doc]

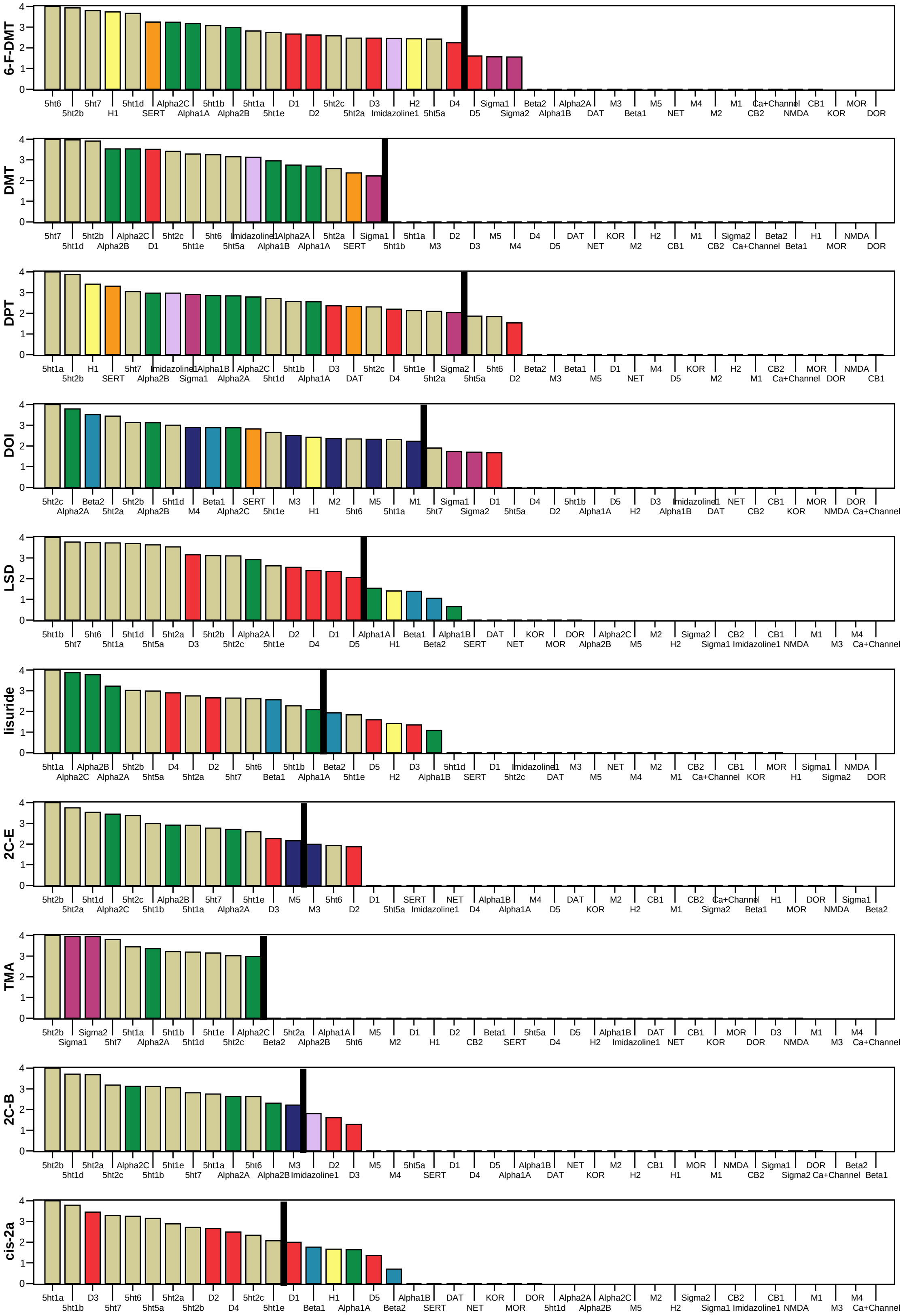
6-F-DMT
DMT
DPT
DOI
LSD
lisuride
2C-E
TMA
2C-B
cis-2a
0 1 2 3 4
5ht6 5ht7 5ht1d Alpha2C 5ht1b 5ht1a D1 5ht2c D3 H2 D4 Sigma1 Beta2 Alpha2A M3 M5 M4 M1 Ca+Channel CB1 MOR
5ht2b H1 SERT Alpha1A Alpha2B 5ht1e D2 5ht2a Imidazoline1 5ht5a D5 Sigma2 Alpha1B DAT Beta1 NET M2 CB2 NMDA KOR DOR
5ht7 5ht2b Alpha2C 5ht2c 5ht6 Imidazoline1 Alpha2A 5ht2a Sigma1 5ht1a D2 M5 D4 DAT KOR H2 M1 Sigma2 Beta2 H1 NMDA
5ht1d Alpha2B D1 5ht1e 5ht5a Alpha1B Alpha1A SERT 5ht1b M3 D3 M4 D5 NET M2 CB1 CB2 Ca+Channel Beta1 MOR DOR
5ht1a H1 5ht7 Imidazoline1 Alpha1B Alpha2C 5ht1b D3 5ht2c 5ht1e Sigma2 5ht6 Beta2 Beta1 D1 M4 KOR H2 CB2 MOR NMDA
5ht2b SERT Alpha2B Sigma1 Alpha2A 5ht1d Alpha1A DAT D4 5ht2a 5ht5a D2 M3 M5 NET D5 M2 M1 Ca+Channel DOR CB1
5ht2c Beta2 5ht2b 5ht1d Beta1 SERT M3 M2 M5 M1 Sigma1 D1 D4 5ht1b D5 D3 Imidazoline1 NET CB1 MOR DOR
Alpha2A 5ht2a Alpha2B M4 Alpha2C 5ht1e H1 5ht6 5ht1a 5ht7 Sigma2 5ht5a D2 Alpha1A H2 Alpha1B DAT CB2 KOR NMDA Ca+Channel
5ht1b 5ht6 5ht1d 5ht2a 5ht2b Alpha2A D2 D1 Alpha1A Beta1 Alpha1B DAT KOR DOR Alpha2C M2 Sigma2 CB2 CB1 M1 M4
5ht7 5ht1a 5ht5a D3 5ht2c 5ht1e D4 D5 H1 Beta2 SERT NET MOR Alpha2B M5 H2 Sigma1 Imidazoline1 NMDA M3 Ca+Channel
5ht1a Alpha2B 5ht2b D4 D2 5ht6 5ht1b Beta2 D5 D3 5ht1d D1 Imidazoline1 M3 NET M2 CB2 CB1 MOR Sigma1 NMDA
Alpha2C Alpha2A 5ht5a 5ht2a 5ht7 Beta1 Alpha1A 5ht1e H2 Alpha1B SERT 5ht2c DAT M5 M4 M1 Ca+Channel KOR H1 Sigma2 DOR
5ht2b 5ht1d 5ht2c Alpha2B 5ht7 5ht1e M5 5ht6 D1 SERT NET Alpha1B M4 DAT M2 CB1 CB2 Ca+Channel H1 DOR Sigma1
5ht2a Alpha2C 5ht1b 5ht1a Alpha2A D3 M3 D2 5ht5a Imidazoline1 D4 Alpha1A D5 KOR H2 M1 Sigma2 Beta1 MOR NMDA Beta2
5ht2b Sigma2 5ht1a 5ht1b 5ht1e Alpha2C 5ht2a Alpha1A M5 D1 D2 Beta1 5ht5a D5 Alpha1B DAT CB1 MOR D3 M1 M4
Sigma1 5ht7 Alpha2A 5ht1d 5ht2c Beta2 Alpha2B 5ht6 M2 H1 CB2 SERT D4 H2 Imidazoline1 NET KOR DOR NMDA M3 Ca+Channel
5ht2b 5ht2a Alpha2C 5ht1e 5ht1a 5ht6 M3 D2 M5 5ht5a D1 D5 Alpha1B NET M2 CB1 MOR NMDA Sigma1 DOR Beta2
5ht1d 5ht2c 5ht1b 5ht7 Alpha2A Alpha2B Imidazoline1 D3 M4 SERT D4 Alpha1A DAT KOR H2 H1 M1 CB2 Sigma2 Ca+Channel Beta1
5ht1a D3 5ht6 5ht2a D2 5ht2c D1 H1 D5 Alpha1B DAT KOR DOR Alpha2A Alpha2C M2 Sigma2 CB2 CB1 M1 M4
5ht1b 5ht7 5ht5a 5ht2b D4 5ht1e Beta1 Alpha1A Beta2 SERT NET MOR 5ht1d Alpha2B M5 H2 Sigma1 Imidazoline1 NMDA M3 Ca+Channel

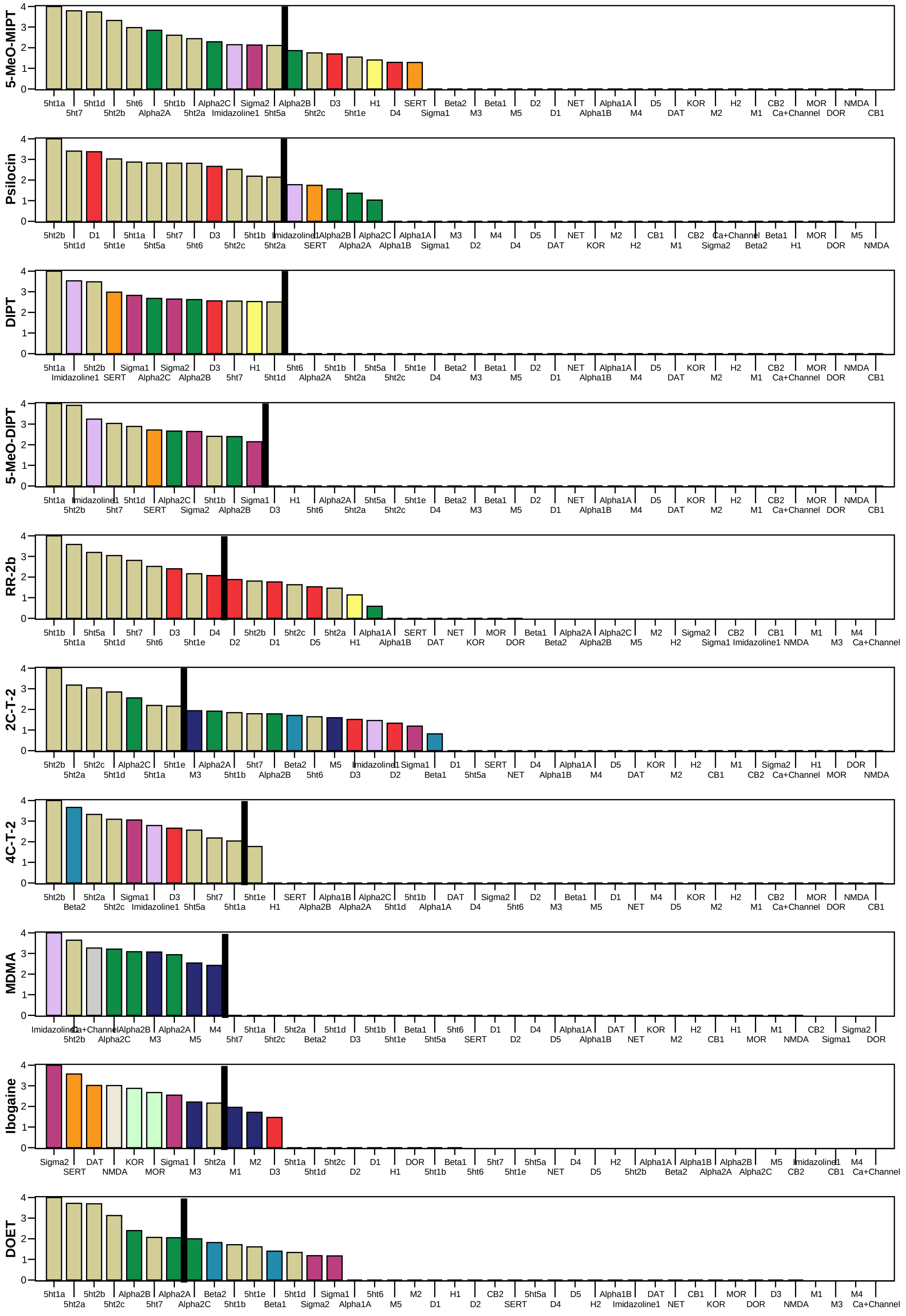
5-MeO-MIPT
Psilocin
DIPT
5-MeO-DIPT
RR-2b
2C-T-2
4C-T-2
MDMA
Ibogaine
DOET

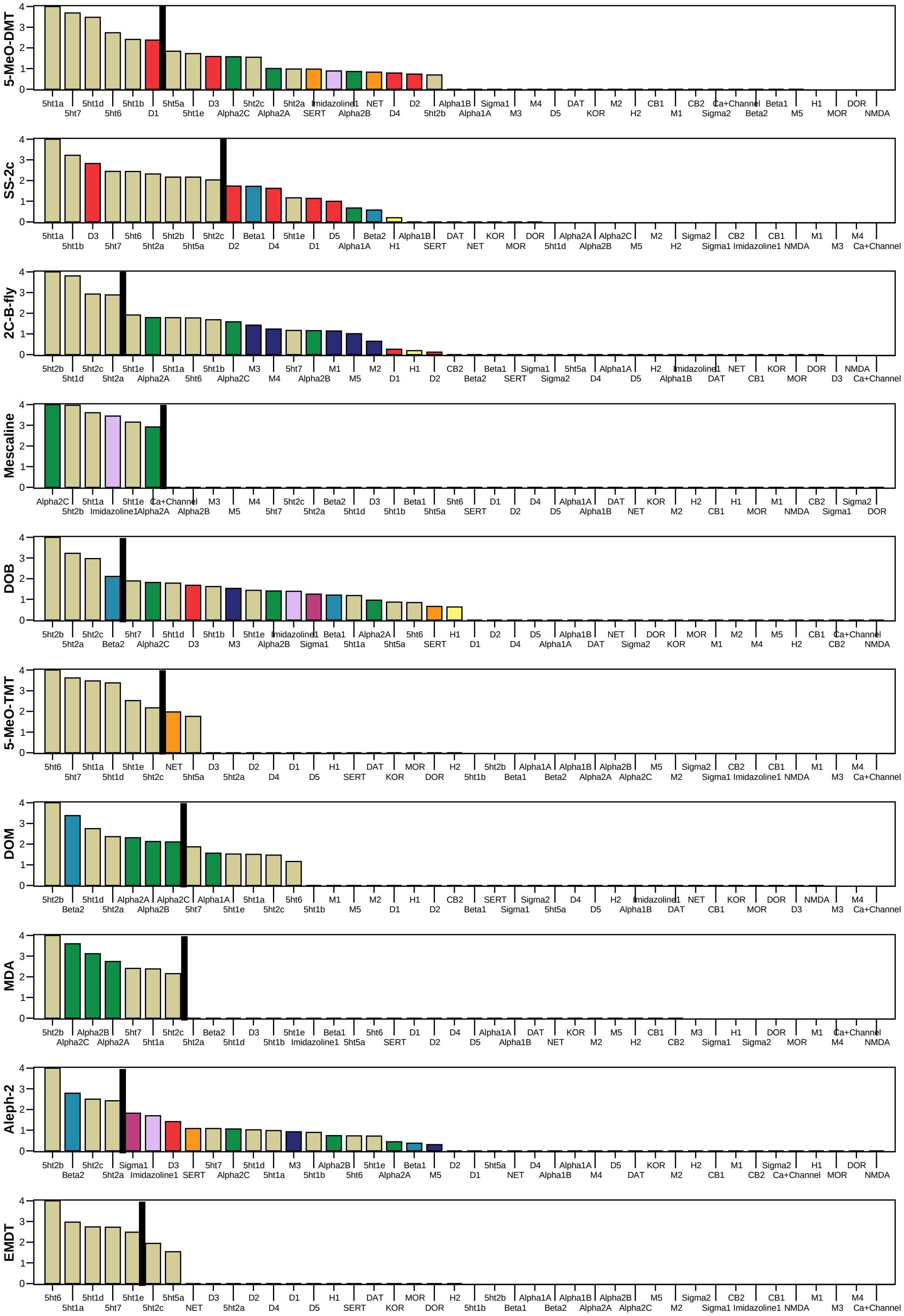
5-MeO-DMT
0 1 2 3 4
5ht1a 5ht7 5ht1d 5ht6 5ht1b D1 5ht5a 5ht1e D3 Alpha2C 5ht2c Alpha2A 5ht2a SERT Imidazoline1 Alpha2B NET D4 D2 5ht2b Alpha1B Alpha1A Sigma1 M3 M4 D5 DAT KOR M2 H2 CB1 M1 CB2 Sigma2 Ca+Channel Beta2 Beta1 M5 H1 MOR DOR NMDA
SS-2c
0 1 2 3 4
5ht1a 5ht1b D3 5ht7 5ht6 5ht2a 5ht2b 5ht5a 5ht2c D2 Beta1 D4 5ht1e D1 D5 Alpha1A Beta2 H1 Alpha1B SERT DAT NET KOR MOR DOR 5ht1d Alpha2A Alpha2B Alpha2C M5 M2 H2 Sigma2 Sigma1 CB2 Imidazoline1 CB1 NMDA H1 M3 M4 Ca+Channel
2C-B-fly
0 1 2 3 4
5ht2b 5ht1d 5ht2c 5ht2a 5ht1e Alpha2A 5ht1a 5ht6 5ht1b Alpha2C M3 M4 5ht7 Alpha2B M1 M5 M2 D1 H1 D2 CB2 Beta2 Beta1 SERT Sigma1 Sigma2 5ht5a D4 Alpha1A D5 H2 Alpha1B Imidazoline1 DAT NET CB1 KOR MOR DOR D3 NMDA Ca+Channel
Mescaline
0 1 2 3 4
Alpha2C 5ht2b 5ht1a Imidazoline1 5ht1e Alpha2A Ca+Channel Alpha2B M3 M5 M4 5ht7 5ht2c 5ht2a Beta2 5ht1d D3 5ht1b Beta1 5ht5a 5ht6 SERT D1 D2 D4 D5 Alpha1A Alpha1B DAT NET KOR M2 H2 CB1 H1 MOR M1 NMDA CB2 Sigma1 Sigma2 DOR
DOB
0 1 2 3 4
5ht2b 5ht2a 5ht2c Beta2 5ht7 Alpha2C 5ht1d D3 5ht1b M3 5ht1e Alpha2B Imidazoline1 Sigma1 Beta1 5ht1a Alpha2A 5ht5a 5ht6 SERT H1 D1 D2 D4 D5 Alpha1A Alpha1B DAT NET Sigma2 DOR KOR MOR M1 M2 M4 M5 H2 CB1 CB2 Ca+Channel NMDA
5-MeO-TMT
0 1 2 3 4
5ht6 5ht7 5ht1a 5ht1d 5ht1e 5ht2c NET 5ht5a D3 5ht2a D2 D4 D1 D5 H1 SERT DAT KOR MOR DOR H2 5ht1b 5ht2b Beta1 Alpha1A Beta2 Alpha1B Alpha2A Alpha2B Alpha2C M5 M2 Sigma2 Sigma1 CB2 Imidazoline1 CB1 NMDA M1 M3 M4 Ca+Channel
DOM
0 1 2 3 4
5ht2b Beta2 5ht1d 5ht2a Alpha2A Alpha2B Alpha2C 5ht7 Alpha1A 5ht1e 5ht1a 5ht2c 5ht6 5ht1b M1 M5 M2 D1 H1 D2 CB2 Beta1 SERT Sigma1 Sigma2 5ht5a D4 D5 H2 Alpha1B Imidazoline1 DAT NET CB1 KOR MOR DOR D3 NMDA M3 M4 Ca+Channel
MDA
0 1 2 3 4
5ht2b Alpha2C Alpha2B Alpha2A 5ht7 5ht1a 5ht2c 5ht2a Beta2 5ht1d D3 5ht1b 5ht1e Imidazoline1 Beta1 5ht5a 5ht6 SERT D1 D2 D4 D5 Alpha1A Alpha1B DAT NET KOR M2 M5 H2 CB1 CB2 M3 Sigma1 H1 Sigma2 DOR MOR M1 M4 Ca+Channel NMDA
Aleph-2
0 1 2 3 4
5ht2b Beta2 5ht2c 5ht2a Sigma1 Imidazoline1 D3 SERT 5ht7 Alpha2C 5ht1d 5ht1a M3 5ht1b Alpha2B 5ht6 5ht1e Alpha2A Beta1 M5 D2 D1 5ht5a NET D4 Alpha1B Alpha1A M4 D5 DAT KOR M2 H2 CB1 M1 CB2 Sigma2 Ca+Channel H1 MOR DOR NMDA
EMDT
0 1 2 3 4
5ht6 5ht1a 5ht1d 5ht7 5ht1e 5ht2c 5ht5a NET D3 5ht2a D2 D4 D1 D5 H1 SERT DAT KOR MOR DOR H2 5ht1b 5ht2b Beta1 Alpha1A Beta2 Alpha1B Alpha2A Alpha2B Alpha2C M5 M2 Sigma2 Sigma1 CB2 Imidazoline1 CB1 NMDA M1 M3 M4 Ca+Channel

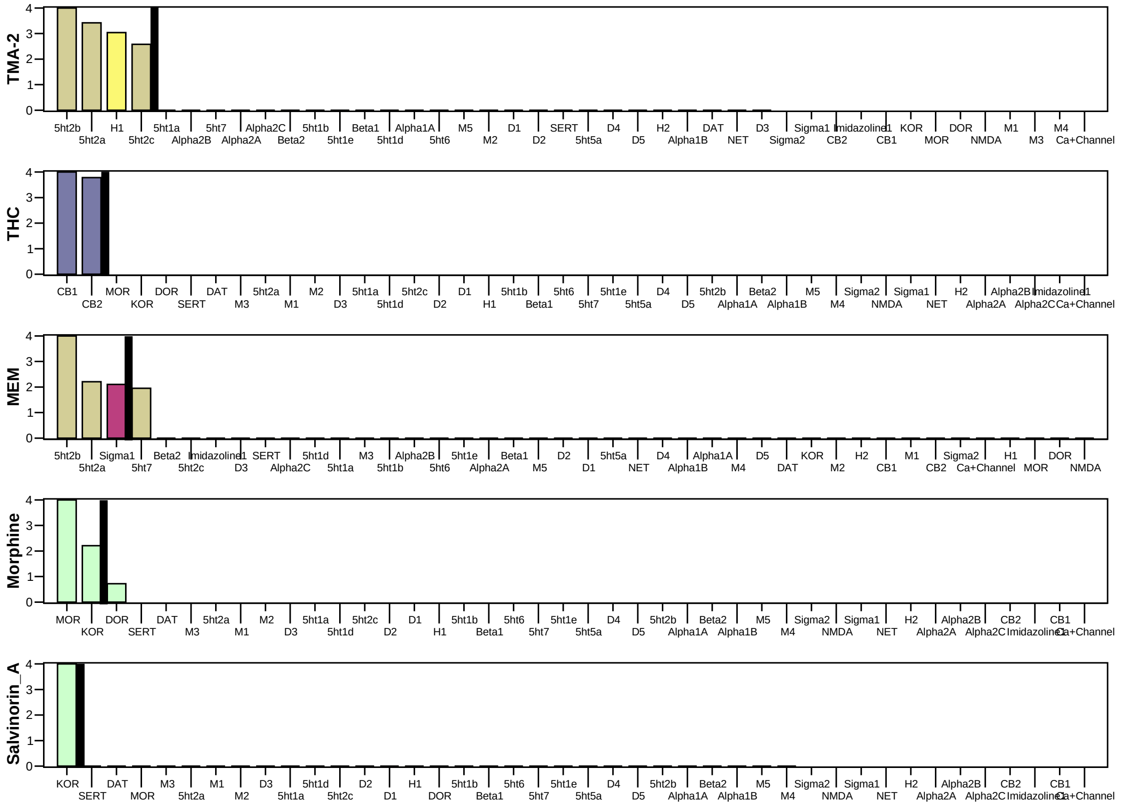
TMA-2
0 1 2 3 4
5ht2b 5ht2a H1 5ht2c 5ht1a Alpha2B 5ht7 Alpha2A Alpha2C Beta2 5ht1b 5ht1e Beta1 5ht1d Alpha1A 5ht6 M5 M2 D1 D2 SERT 5ht5a D4 D5 H2 Alpha1B DAT NET D3 Sigma2 Sigma1 CB2 Imidazoline1 CB1 KOR MOR DOR NMDA M1 M3 M4 Ca+Channel
THC
0 1 2 3 4
CB1 CB2 MOR KOR DOR SERT DAT M3 5ht2a M1 M2 D3 5ht1a 5ht1d 5ht2c D2 D1 H1 5ht1b Beta1 5ht6 5ht7 5ht1e 5ht5a D4 D5 5ht2b Alpha1A Beta2 Alpha1B M5 M4 Sigma2 NMDA Sigma1 NET H2 Alpha2A Alpha2B Alpha2C Imidazoline1 Ca+Channel
MEM
0 1 2 3 4
5ht2b 5ht2a Sigma1 5ht7 Beta2 5ht2c Imidazoline1 D3 SERT Alpha2C 5ht1d 5ht1a M3 5ht1b Alpha2B 5ht6 5ht1e Alpha2A Beta1 M5 D2 D1 5ht5a NET D4 Alpha1B Alpha1A M4 D5 DAT KOR M2 H2 CB1 M1 CB2 Sigma2 Ca+Channel H1 MOR DOR NMDA
Morphine
0 1 2 3 4
MOR KOR DOR SERT DAT M3 5ht2a M1 M2 D3 5ht1a 5ht1d 5ht2c D2 D1 H1 5ht1b Beta1 5ht6 5ht7 5ht1e 5ht5a D4 D5 5ht2b Alpha1A Beta2 Alpha1B M5 M4 Sigma2 NMDA Sigma1 NET H2 Alpha2A Alpha2B Alpha2C CB2 Imidazoline1 CB1 Ca+Channel
Salvinorin_A
0 1 2 3 4
KOR SERT DAT MOR M3 5ht2a M1 M2 D3 5ht1a 5ht1d 5ht2c D2 D1 H1 DOR 5ht1b Beta1 5ht6 5ht7 5ht1e 5ht5a D4 D5 5ht2b Alpha1A Beta2 Alpha1B M5 M4 Sigma2 NMDA Sigma1 NET H2 Alpha2A Alpha2B Alpha2C CB2 Imidazoline1 CB1 Ca+Channel
