# Supplementary material for: Psychedelics and the Human Receptorome
Source: PLoS One. 2010 Feb 2;5(2):e9019. doi: 10.1371/journal.pone.0009019 (PMC2814854; doi:10.1371/journal.pone.0009019)
Supplement: Supplementary file 10 [file pone.0009019.s003.doc]

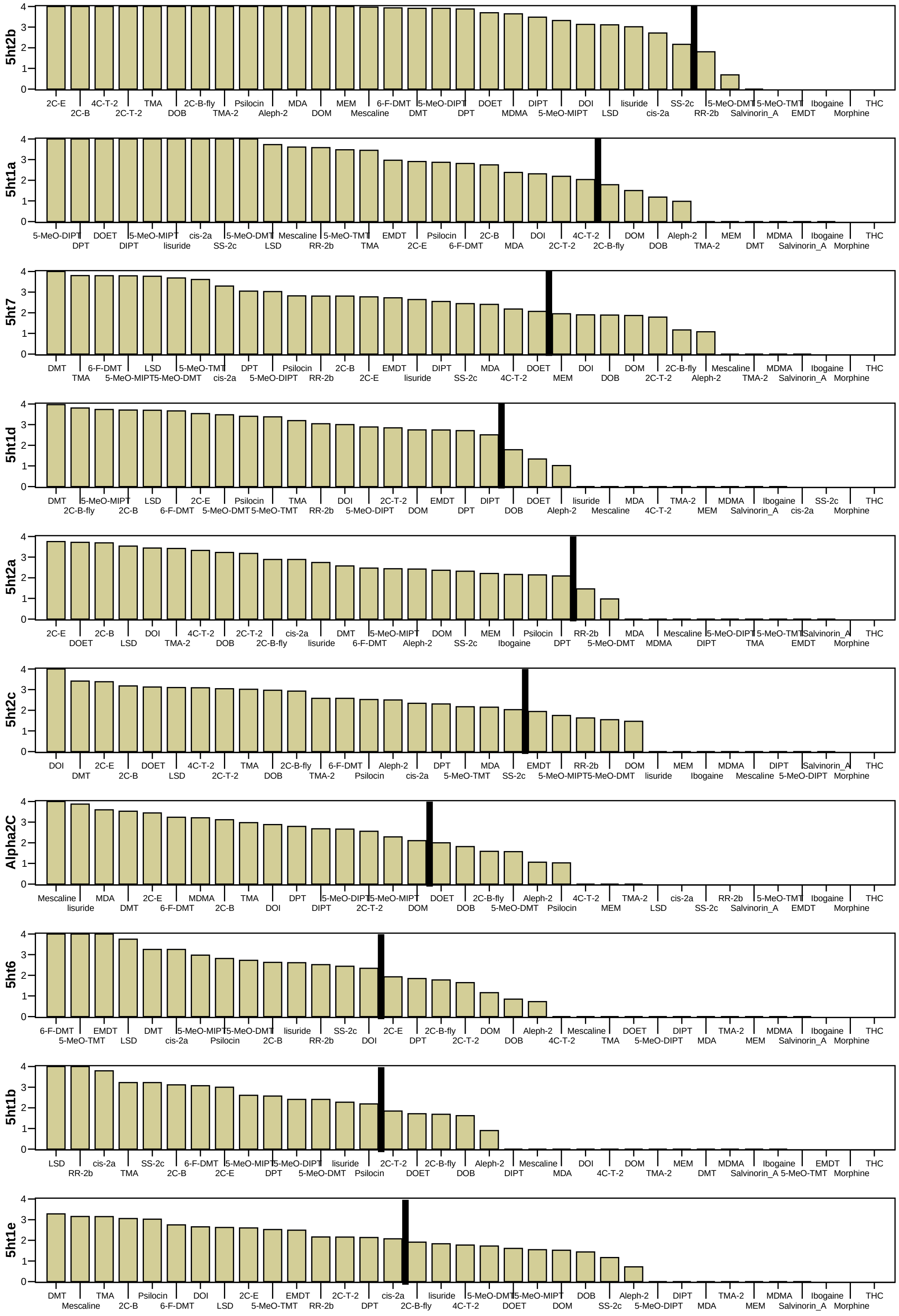
5ht2b
2C-E 4C-T-2 TMA 2C-B-fly Psilocin MDA MEM 6-F-DMT 5-MeO-DIPT DOET DIPT DOI lisuride SS-2c 5-MeO-DMT 5-MeO-TMT Ibogaine THC
2C-B 2C-T-2 DOB TMA-2 Aleph-2 DOM Mescaline DMT DPT MDMA 5-MeO-MIPT LSD cis-2a RR-2b Salvinorin_A EMDT Morphine
5ht1a
5-MeO-DIPT DOET 5-MeO-MIPT cis-2a 5-MeO-DMT Mescaline 5-MeO-TMT EMDT Psilocin 2C-B DOI 4C-T-2 DOM Aleph-2 MEM MDMA Ibogaine THC
DPT DIPT lisuride SS-2c LSD RR-2b TMA 2C-E 6-F-DMT MDA 2C-T-2 2C-B-fly DOB TMA-2 DMT Salvinorin_A Morphine
5ht7
DMT 6-F-DMT LSD 5-MeO-TMT DPT Psilocin 2C-B EMDT DIPT MDA DOET DOI DOM 2C-B-fly Mescaline MDMA Ibogaine THC
TMA 5-MeO-MIPT 5-MeO-DMT cis-2a 5-MeO-DIPT RR-2b 2C-E lisuride SS-2c 4C-T-2 MEM DOB 2C-T-2 Aleph-2 TMA-2 Salvinorin_A Morphine
5ht1d
DMT 5-MeO-MIPT LSD 2C-E Psilocin TMA DOI 2C-T-2 EMDT DIPT DOET lisuride MDA TMA-2 MDMA Ibogaine SS-2c THC
2C-B-fly 2C-B 6-F-DMT 5-MeO-DMT 5-MeO-TMT RR-2b 5-MeO-DIPT DOM DPT DOB Aleph-2 Mescaline 4C-T-2 MEM Salvinorin_A cis-2a Morphine
5ht2a
2C-E 2C-B DOI 4C-T-2 2C-T-2 cis-2a DMT 5-MeO-MIPT DOM MEM Psilocin RR-2b MDA Mescaline 5-MeO-DIPT 5-MeO-TMT Salvinorin_A THC
DOET LSD TMA-2 DOB 2C-B-fly lisuride 6-F-DMT Aleph-2 SS-2c Ibogaine DPT 5-MeO-DMT MDMA DIPT TMA EMDT Morphine
5ht2c
DOI 2C-E DOET 4C-T-2 TMA 2C-B-fly 6-F-DMT Aleph-2 DPT MDA EMDT RR-2b DOM MEM MDMA DIPT Salvinorin_A THC
DMT 2C-B LSD 2C-T-2 DOB TMA-2 Psilocin cis-2a 5-MeO-TMT SS-2c 5-MeO-MIPT 5-MeO-DMT lisuride Ibogaine Mescaline 5-MeO-DIPT Morphine
Alpha2C
Mescaline MDA 2C-E MDMA TMA DPT 5-MeO-DIPT 5-MeO-MIPT DOET 2C-B-fly Aleph-2 4C-T-2 TMA-2 cis-2a RR-2b 5-MeO-TMT Ibogaine THC
lisuride DMT 6-F-DMT 2C-B DOI DIPT 2C-T-2 DOM DOB 5-MeO-DMT Psilocin MEM LSD SS-2c Salvinorin_A EMDT Morphine
5ht6
6-F-DMT EMDT DMT 5-MeO-MIPT 5-MeO-DMT lisuride SS-2c 2C-E 2C-B-fly DOM Aleph-2 Mescaline DOET DIPT TMA-2 MDMA Ibogaine THC
5-MeO-TMT LSD cis-2a Psilocin 2C-B RR-2b DOI DPT 2C-T-2 DOB 4C-T-2 TMA 5-MeO-DIPT MDA MEM Salvinorin_A Morphine
5ht1b
LSD cis-2a SS-2c 6-F-DMT 5-MeO-MIPT 5-MeO-DIPT lisuride 2C-T-2 2C-B-fly Aleph-2 Mescaline DOI DOM MEM MDMA Ibogaine EMDT THC
RR-2b TMA 2C-B 2C-E DPT 5-MeO-DMT Psilocin DOET DOB DIPT MDA 4C-T-2 TMA-2 DMT Salvinorin_A 5-MeO-TMT Morphine
5ht1e
DMT TMA Psilocin DOI 2C-E EMDT 2C-T-2 cis-2a lisuride 5-MeO-DMT 5-MeO-MIPT DOB Aleph-2 DIPT TMA-2 MDMA Ibogaine THC
Mescaline 2C-B 6-F-DMT LSD 5-MeO-TMT RR-2b DPT 2C-B-fly 4C-T-2 DOET DOM SS-2c 5-MeO-DIPT MDA MEM Salvinorin_A Morphine

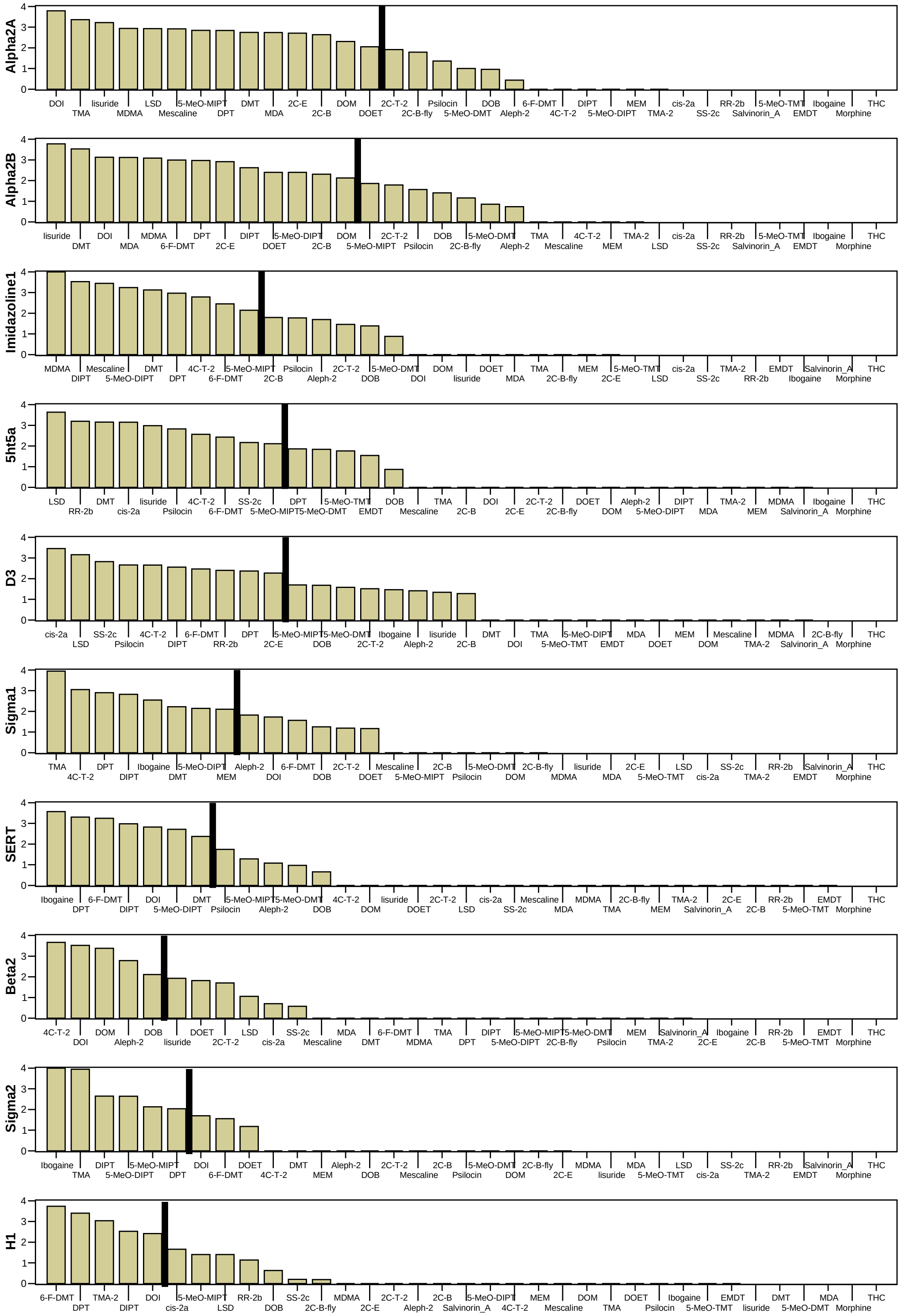
Alpha2A
DOI lisuride LSD 5-MeO-MIPT DMT 2C-E DOM 2C-T-2 Psilocin DOB 6-F-DMT DIPT MEM cis-2a RR-2b 5-MeO-TMT Ibogaine THC
TMA MDMA Mescaline DPT MDA 2C-B DOET 2C-B-fly 5-MeO-DMT Aleph-2 4C-T-2 5-MeO-DIPT TMA-2 SS-2c Salvinorin_A EMDT Morphine
Alpha2B
lisuride DOI MDMA DPT DIPT 5-MeO-DIPT DOM 2C-T-2 DOB 5-MeO-DMT TMA 4C-T-2 TMA-2 cis-2a RR-2b 5-MeO-TMT Ibogaine THC
DMT MDA 6-F-DMT 2C-E DOET 2C-B 5-MeO-MIPT Psilocin 2C-B-fly Aleph-2 Mescaline MEM LSD SS-2c Salvinorin_A EMDT Morphine
Imidazoline1
MDMA Mescaline DMT 4C-T-2 5-MeO-MIPT Psilocin 2C-T-2 5-MeO-DMT DOM DOET TMA MEM 5-MeO-TMT cis-2a TMA-2 EMDT Salvinorin_A THC
DIPT 5-MeO-DIPT DPT 6-F-DMT 2C-B Aleph-2 DOB DOI lisuride MDA 2C-B-fly 2C-E LSD SS-2c RR-2b Ibogaine Morphine
5ht5a
LSD DMT lisuride 4C-T-2 SS-2c DPT 5-MeO-TMT DOB TMA DOI 2C-T-2 DOET Aleph-2 DIPT TMA-2 MDMA Ibogaine THC
RR-2b cis-2a Psilocin 6-F-DMT 5-MeO-MIPT 5-MeO-DMT EMDT Mescaline 2C-B 2C-E 2C-B-fly DOM 5-MeO-DIPT MDA MEM Salvinorin_A Morphine
D3
cis-2a SS-2c 4C-T-2 6-F-DMT DPT 5-MeO-MIPT 5-MeO-DMT Ibogaine lisuride DMT TMA 5-MeO-DIPT MDA MEM Mescaline MDMA 2C-B-fly THC
LSD Psilocin DIPT RR-2b 2C-E DOB 2C-T-2 Aleph-2 2C-B DOI 5-MeO-TMT EMDT DOET DOM TMA-2 Salvinorin_A Morphine
Sigma1
TMA DPT Ibogaine 5-MeO-DIPT Aleph-2 6-F-DMT 2C-T-2 Mescaline 2C-B 5-MeO-DMT 2C-B-fly lisuride 2C-E LSD SS-2c RR-2b Salvinorin_A THC
4C-T-2 DIPT DMT MEM DOI DOB DOET 5-MeO-MIPT Psilocin DOM MDMA MDA 5-MeO-TMT cis-2a TMA-2 EMDT Morphine
SERT
Ibogaine 6-F-DMT DOI DMT 5-MeO-MIPT 5-MeO-DMT 4C-T-2 lisuride 2C-T-2 cis-2a Mescaline MDMA 2C-B-fly TMA-2 2C-E RR-2b EMDT THC
DPT DIPT 5-MeO-DIPT Psilocin Aleph-2 DOB DOM DOET LSD SS-2c MDA TMA MEM Salvinorin_A 2C-B 5-MeO-TMT Morphine
Beta2
4C-T-2 DOM DOB DOET LSD SS-2c MDA 6-F-DMT TMA DIPT 5-MeO-MIPT 5-MeO-DMT MEM Salvinorin_A Ibogaine RR-2b EMDT THC
DOI Aleph-2 lisuride 2C-T-2 cis-2a Mescaline DMT MDMA DPT 5-MeO-DIPT 2C-B-fly Psilocin TMA-2 2C-E 2C-B 5-MeO-TMT Morphine
Sigma2
Ibogaine DIPT 5-MeO-MIPT DOI DOET DMT Aleph-2 2C-T-2 2C-B 5-MeO-DMT 2C-B-fly MDMA MDA LSD SS-2c RR-2b Salvinorin_A THC
TMA 5-MeO-DIPT DPT 6-F-DMT 4C-T-2 MEM DOB Mescaline Psilocin DOM 2C-E lisuride 5-MeO-TMT cis-2a TMA-2 EMDT Morphine
H1
6-F-DMT TMA-2 DOI 5-MeO-MIPT RR-2b SS-2c MDMA 2C-T-2 2C-B 5-MeO-DIPT MEM DOM DOET Ibogaine EMDT DMT MDA THC
DPT DIPT cis-2a LSD DOB 2C-B-fly 2C-E Aleph-2 Salvinorin_A 4C-T-2 Mescaline TMA Psilocin 5-MeO-TMT lisuride 5-MeO-DMT Morphine
0 1 2 3 4

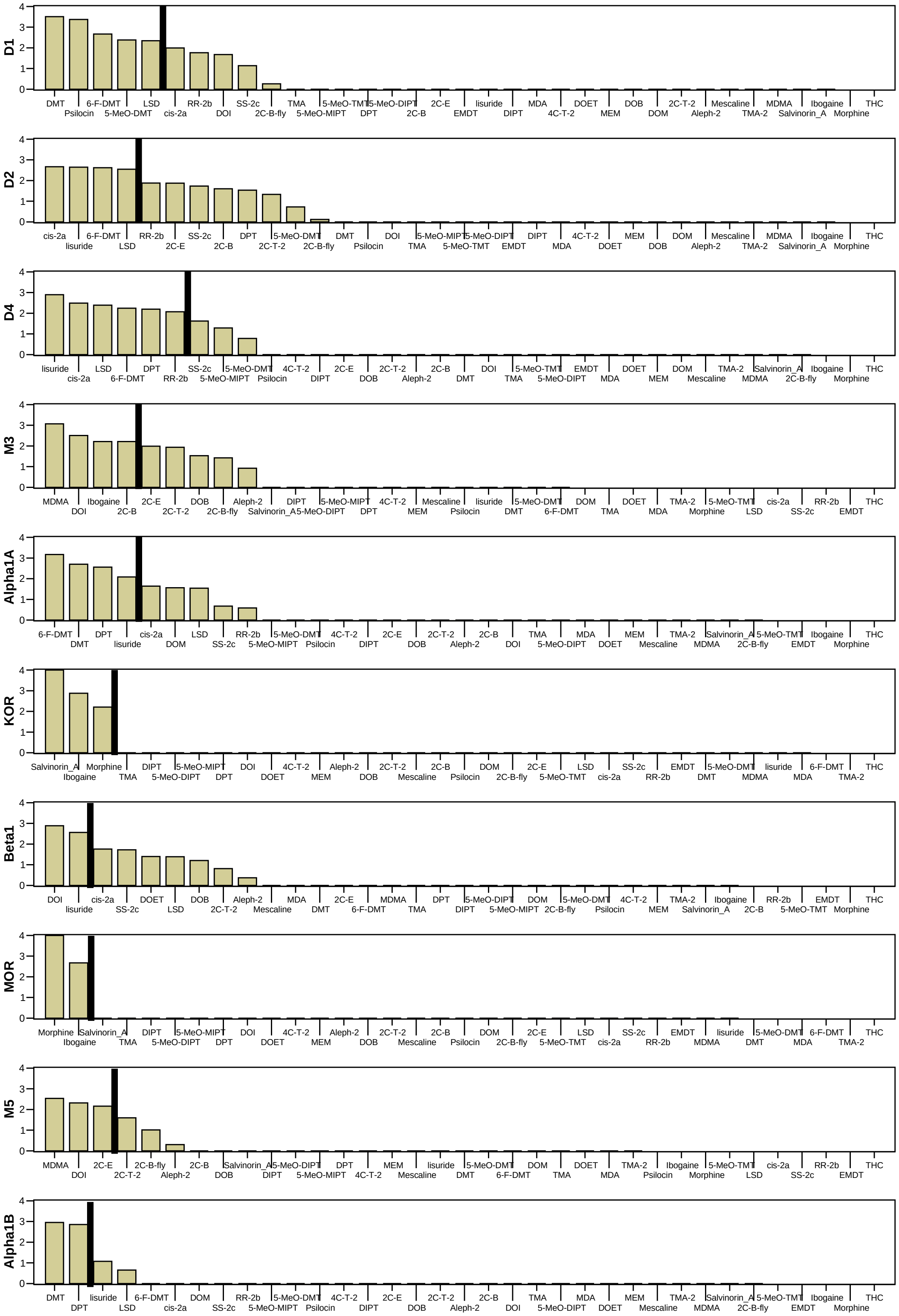
D1
0 1 2 3 4
DMT 6-F-DMT LSD RR-2b SS-2c TMA 5-MeO-TMT 5-MeO-DIPT 2C-E lisuride MDA DOET DOB 2C-T-2 Mescaline MDMA Ibogaine THC
Psilocin 5-MeO-DMT cis-2a DOI 2C-B-fly 5-MeO-MIPT DPT 2C-B EMDT DIPT 4C-T-2 MEM DOM Aleph-2 TMA-2 Salvinorin_A Morphine
D2
0 1 2 3 4
cis-2a 6-F-DMT RR-2b SS-2c DPT 5-MeO-DMT DMT DOI 5-MeO-MIPT 5-MeO-DIPT DIPT 4C-T-2 MEM DOM Mescaline MDMA Ibogaine THC
lisuride LSD 2C-E 2C-B 2C-T-2 2C-B-fly Psilocin TMA 5-MeO-TMT EMDT MDA DOET DOB Aleph-2 TMA-2 Salvinorin_A Morphine
D4
0 1 2 3 4
lisuride LSD DPT SS-2c 5-MeO-DMT 4C-T-2 2C-E 2C-T-2 2C-B DOI 5-MeO-TMT EMDT DOET DOM TMA-2 Salvinorin_A Ibogaine THC
cis-2a 6-F-DMT RR-2b 5-MeO-MIPT Psilocin DIPT DOB Aleph-2 DMT TMA 5-MeO-DIPT MDA MEM Mescaline MDMA 2C-B-fly Morphine
M3
0 1 2 3 4
MDMA Ibogaine 2C-E DOB Aleph-2 DIPT 5-MeO-MIPT 4C-T-2 Mescaline lisuride 5-MeO-DMT DOM DOET TMA-2 5-MeO-TMT cis-2a RR-2b THC
DOI 2C-B 2C-T-2 2C-B-fly Salvinorin_A 5-MeO-DIPT DPT MEM Psilocin DMT 6-F-DMT TMA MDA Morphine LSD SS-2c EMDT
Alpha1A
0 1 2 3 4
6-F-DMT DPT cis-2a LSD RR-2b 5-MeO-DMT 4C-T-2 2C-E 2C-T-2 2C-B TMA MDA MEM TMA-2 Salvinorin_A 5-MeO-TMT Ibogaine THC
DMT lisuride DOM SS-2c 5-MeO-MIPT Psilocin DIPT DOB Aleph-2 DOI 5-MeO-DIPT DOET Mescaline MDMA 2C-B-fly EMDT Morphine
KOR
0 1 2 3 4
Salvinorin_A Morphine DIPT 5-MeO-MIPT DOI 4C-T-2 Aleph-2 2C-T-2 2C-B DOM 2C-E LSD SS-2c EMDT 5-MeO-DMT lisuride 6-F-DMT THC
Ibogaine TMA 5-MeO-DIPT DPT DOET MEM DOB Mescaline Psilocin 2C-B-fly 5-MeO-TMT cis-2a RR-2b DMT MDMA MDA TMA-2
Beta1
0 1 2 3 4
DOI cis-2a DOET DOB Aleph-2 MDA 2C-E MDMA DPT 5-MeO-DIPT DOM 5-MeO-DMT 4C-T-2 TMA-2 Ibogaine RR-2b EMDT THC
lisuride SS-2c LSD 2C-T-2 Mescaline DMT 6-F-DMT TMA DIPT 5-MeO-MIPT 2C-B-fly Psilocin MEM Salvinorin_A 2C-B 5-MeO-TMT Morphine
MOR
0 1 2 3 4
Morphine Salvinorin_A DIPT 5-MeO-MIPT DOI 4C-T-2 Aleph-2 2C-T-2 2C-B DOM 2C-E LSD SS-2c EMDT lisuride 5-MeO-DMT 6-F-DMT THC
Ibogaine TMA 5-MeO-DIPT DPT DOET MEM DOB Mescaline Psilocin 2C-B-fly 5-MeO-TMT cis-2a RR-2b MDMA DMT MDA TMA-2
M5
0 1 2 3 4
MDMA 2C-E 2C-B-fly 2C-B Salvinorin_A 5-MeO-DIPT DPT MEM lisuride 5-MeO-DMT DOM DOET TMA-2 Ibogaine 5-MeO-TMT cis-2a RR-2b THC
DOI 2C-T-2 Aleph-2 DOB DIPT 5-MeO-MIPT 4C-T-2 Mescaline DMT 6-F-DMT TMA MDA Psilocin Morphine LSD SS-2c EMDT
Alpha1B
0 1 2 3 4
DMT lisuride 6-F-DMT DOM RR-2b 5-MeO-DMT 4C-T-2 2C-E 2C-T-2 2C-B TMA MDA MEM TMA-2 Salvinorin_A 5-MeO-TMT Ibogaine THC
DPT LSD cis-2a SS-2c 5-MeO-MIPT Psilocin DIPT DOB Aleph-2 DOI 5-MeO-DIPT DOET Mescaline MDMA 2C-B-fly EMDT Morphine

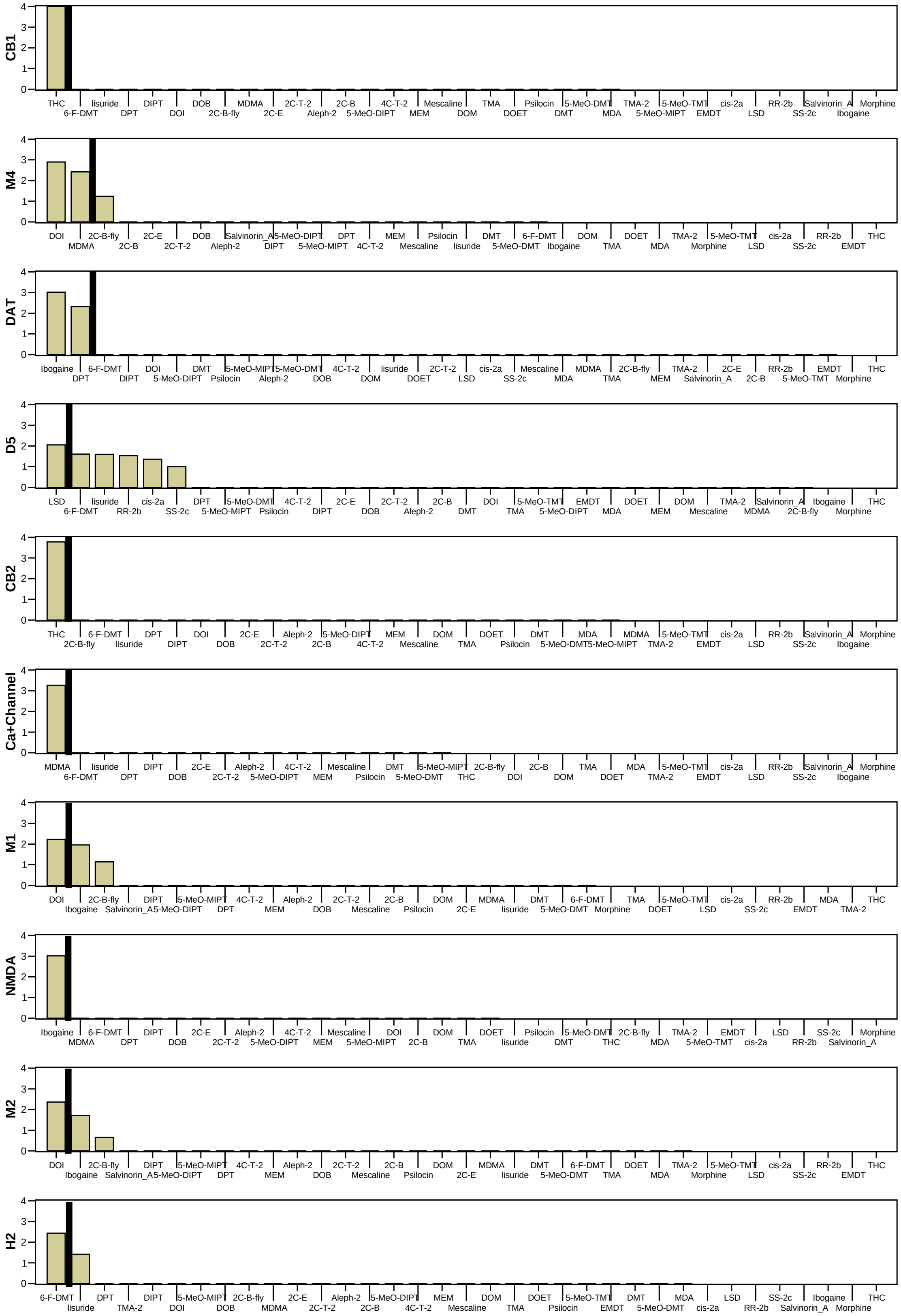
CB1
0 1 2 3 4
THC lisuride DIPT DOB MDMA 2C-T-2 2C-B 4C-T-2 Mescaline TMA Psilocin 5-MeO-DMT TMA-2 5-MeO-TMT cis-2a RR-2b Salvinorin_A Morphine
6-F-DMT DPT DOI 2C-B-fly 2C-E Aleph-2 5-MeO-DIPT MEM DOM DOET DMT MDA 5-MeO-MIPT EMDT LSD SS-2c Ibogaine
M4
0 1 2 3 4
DOI 2C-B-fly 2C-E DOB Salvinorin_A 5-MeO-DIPT DPT MEM Psilocin DMT 6-F-DMT DOM DOET TMA-2 5-MeO-TMT cis-2a RR-2b THC
MDMA 2C-B 2C-T-2 Aleph-2 DIPT 5-MeO-MIPT 4C-T-2 Mescaline lisuride 5-MeO-DMT Ibogaine TMA MDA Morphine LSD SS-2c EMDT
DAT
0 1 2 3 4
Ibogaine 6-F-DMT DOI DMT 5-MeO-MIPT 5-MeO-DMT 4C-T-2 lisuride 2C-T-2 cis-2a Mescaline MDMA 2C-B-fly TMA-2 2C-E RR-2b EMDT THC
DPT DIPT 5-MeO-DIPT Psilocin Aleph-2 DOB DOM DOET LSD SS-2c MDA TMA MEM Salvinorin_A 2C-B 5-MeO-TMT Morphine
D5
0 1 2 3 4
LSD lisuride cis-2a DPT 5-MeO-DMT 4C-T-2 2C-E 2C-T-2 2C-B DOI 5-MeO-TMT EMDT DOET DOM TMA-2 Salvinorin_A Ibogaine THC
6-F-DMT RR-2b SS-2c 5-MeO-MIPT Psilocin DIPT DOB Aleph-2 DMT TMA 5-MeO-DIPT MDA MEM Mescaline MDMA 2C-B-fly Morphine
CB2
0 1 2 3 4
THC 6-F-DMT DPT DOI 2C-E Aleph-2 5-MeO-DIPT MEM DOM DOET DMT MDA MDMA 5-MeO-TMT cis-2a RR-2b Salvinorin_A Morphine
2C-B-fly lisuride DIPT DOB 2C-T-2 2C-B 4C-T-2 Mescaline TMA Psilocin 5-MeO-DMT 5-MeO-MIPT TMA-2 EMDT LSD SS-2c Ibogaine
Ca+Channel
0 1 2 3 4
MDMA lisuride DIPT 2C-E Aleph-2 4C-T-2 Mescaline DMT 5-MeO-MIPT 2C-B-fly 2C-B TMA MDA 5-MeO-TMT cis-2a RR-2b Salvinorin_A Morphine
6-F-DMT DPT DOB 2C-T-2 5-MeO-DIPT MEM Psilocin 5-MeO-DMT THC DOI DOM DOET TMA-2 EMDT LSD SS-2c Ibogaine
M1
0 1 2 3 4
DOI 2C-B-fly DIPT 5-MeO-MIPT 4C-T-2 Aleph-2 2C-T-2 2C-B DOM MDMA DMT 6-F-DMT TMA 5-MeO-TMT cis-2a RR-2b MDA THC
Ibogaine Salvinorin_A 5-MeO-DIPT DPT MEM DOB Mescaline Psilocin 2C-E lisuride 5-MeO-DMT Morphine DOET LSD SS-2c EMDT TMA-2
NMDA
0 1 2 3 4
Ibogaine 6-F-DMT DIPT 2C-E Aleph-2 4C-T-2 Mescaline DOI DOM DOET Psilocin 5-MeO-DMT 2C-B-fly TMA-2 EMDT LSD SS-2c Morphine
MDMA DPT DOB 2C-T-2 5-MeO-DIPT MEM 5-MeO-MIPT 2C-B TMA lisuride DMT THC MDA 5-MeO-TMT cis-2a RR-2b Salvinorin_A
M2
0 1 2 3 4
DOI 2C-B-fly DIPT 5-MeO-MIPT 4C-T-2 Aleph-2 2C-T-2 2C-B DOM MDMA DMT 6-F-DMT DOET TMA-2 5-MeO-TMT cis-2a RR-2b THC
Ibogaine Salvinorin_A 5-MeO-DIPT DPT MEM DOB Mescaline Psilocin 2C-E lisuride 5-MeO-DMT TMA MDA Morphine LSD SS-2c EMDT
H2
0 1 2 3 4
6-F-DMT DPT DIPT 5-MeO-MIPT 2C-B-fly 2C-E Aleph-2 5-MeO-DIPT MEM DOM DOET 5-MeO-TMT DMT MDA LSD SS-2c Ibogaine THC
lisuride TMA-2 DOI DOB MDMA 2C-T-2 2C-B 4C-T-2 Mescaline TMA Psilocin EMDT 5-MeO-DMT cis-2a RR-2b Salvinorin_A Morphine

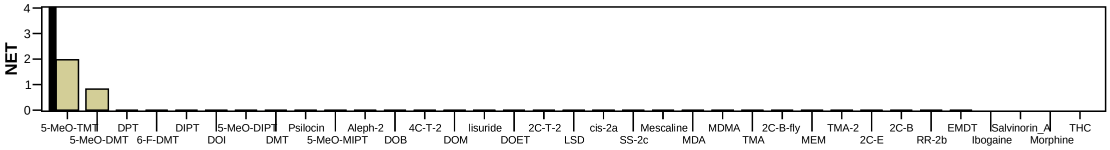
NET
0
1
2
3
4
5-MeO-TMT
5-MeO-DMT
DPT
6-F-DMT
DIPT
DOI
5-MeO-DIPT
DMT
Psilocin
5-MeO-MIPT
Aleph-2
DOB
4C-T-2
DOM
lisuride
DOET
2C-T-2
LSD
cis-2a
SS-2c
Mescaline
MDA
MDMA
TMA
2C-B-fly
MEM
TMA-2
2C-E
2C-B
RR-2b
EMDT
Ibogaine
Salvinorin_A
Morphine
THC

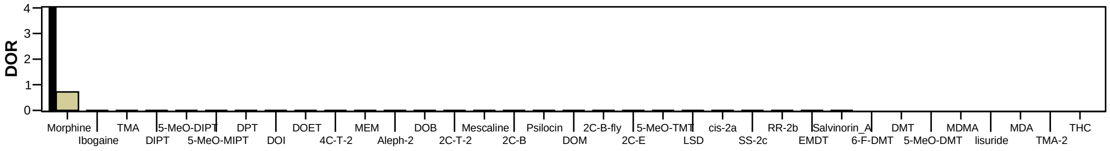
DOR
0
1
2
3
4
Morphine
Ibogaine
TMA
DIPT
5-MeO-DIPT
5-MeO-MIPT
DPT
DOI
DOET
4C-T-2
MEM
Aleph-2
DOB
2C-T-2
Mescaline
2C-B
Psilocin
DOM
2C-B-fly
2C-E
5-MeO-TMT
LSD
cis-2a
SS-2c
RR-2b
EMDT
Salvinorin_A
6-F-DMT
DMT
5-MeO-DMT
MDMA
lisuride
MDA
TMA-2
THC
